# The Suicidal Patient in the Emergency Department Team-Based Learning Activity

**DOI:** 10.21980/J8892X

**Published:** 2023-01-31

**Authors:** Caroline Stoddard Astemborski, Sara Dimeo

**Affiliations:** * Prisma Health Upstate, University of South Carolina School of Medicine Greenville, Department of Emergency Medicine, Greenville, SC; ^ East Valley Hospital, Department of Emergency Medicine, Chandler, AZ

## Abstract

**Audience:**

Emergency medicine resident physicians, (PGY1-4), medical students rotating in the emergency department

**Introduction/Background:**

Emergency physicians have a duty to recognize and provide care for patients who attempt to harm themselves or commit suicide. Mental health-related chief complaints account for 12.5% of emergency department (ED) visits.^1^ Additionally, patients with depressive symptoms who are discharged from the ED are at the highest risk for suicidal thoughts and behaviors.^1^ Therefore, evaluating and screening for suicide and determining appropriate dispositions for this patient population is extremely important. This team-based learning (TBL) activity will help prepare residents and medical students to evaluate, recognize, and disposition this at-risk patient population.

**Educational Objectives:**

By the end of the session, participants will be able to: 1) describe risk factors for suicide; 2) summarize the emergency physician’s role in assessing patients with psychiatric emergencies; 3) assess a patient using a mental status evaluation; 4) identify the criteria for involuntary psychiatric hold placement; 5) develop a safe discharge plan for patients experiencing depression; and 6) Formulate a plan for evaluating a suicidal patient who is acutely intoxicated.

**Educational Methods:**

This team-based learning activity is a classic TBL that includes learner responsible content (LRC), an individual readiness assessment test (iRAT), a multiple choice group readiness assessment test (gRAT) with immediate feedback assessment technique (IF/AT), and a group application exercise (GAE).

**Research Methods:**

A post-TBL survey was provided to each participant. A Likert scale was used for the survey questions to assess the relevance of the session to emergency medicine practice, learner perception of knowledge gained, learner perception of improvement of clinical practice, session engagement, and session delivery.

**Results:**

The post-activity evaluation had a response rate of 33% (11/33). Overall, all the participants “strongly agreed” (Likert 5/5) or “agreed” (Likert 4/5) that the session improved their knowledge of caring for the suicidal patient in the ED with an average score of 4.6/5. All participants “strongly agreed” (Likert 5/5) or “agreed” (Likert 4/5) that the material presented was relevant to their clinical practice in the ED for an average score of 4.6/5. Constructive feedback included requesting learner responsible content (LRC) be sent earlier than one week prior to the activity.

**Discussion:**

Depression and suicidal ideation are common ED complaints. However, it can be difficult to evaluate these patients and select an appropriate disposition because their symptoms can range from benign to life-threatening. The team-based learning (TBL) session allows for discussion of the complexities of the depressed and suicidal patient. Learners found this TBL to be beneficial in providing a diagnostic pathway and treatment algorithm to manage these complex, high-risk patients.

**Topics:**

Suicide, depression, substance abuse, disposition, team-based learning.

## USER GUIDE

**Table t1-jetem-8-1-t1:** 

List of Resources:	
Abstract	1
User Guide	3
Learner Materials	5
iRAT	5
gRAT	7
GAE	10
Instructor Materials	19
RAT Key	20
GAE Key	23
Debrief PowerPoint	38

[Table t1-jetem-8-1-t1] **Learner Audience:**

Medical students, interns, junior residents

**Time Required for Implementation:**

Instructor Preparation: 60–90 minutes

Learner Responsible Content: 30–45 minutes

- Article: 20–30 minutes
- iRAT: 10–15 minutes

In Class Time: 50 minutes

- Introduction: 5 minutes
- gRAT: 10 minutes
- GAE: 25 minutes
- Review: 10 minutes

**Recommended Number of Learners per Instructor:**

This classic TBL can be taught with one instructor, although it may be helpful to have an additional facilitator to circulate the room during the group application exercises to interact and guide the session. Additional facilitators could be chief or senior resident.

**Topics:**

Suicide, depression, substance abuse, disposition, teambased learning.

**Objectives:**

By the end of the session, participants will be able to:

1. Describe risk factors for suicide.
2. Summarize the emergency physician’s role in assessing patients with psychiatric emergencies.
3. Assess a patient using a mental status evaluation.
4. Identify the criteria for involuntary psychiatric hold placement.
5. Develop a safe discharge plan for patients experiencing depression.
6. Formulate a plan for evaluating a suicidal patient who is acutely intoxicated.

### Linked objectives and methods

Case 1 explores symptoms of depression and suicide risk factors (Objectives 1, 2). Cases 2 and 3 examine the components of an appropriate history and physical exam, including conducting a mental status evaluation (Objectives 2, 3). Cases 4A, 4B, and 5 will explore different disposition scenarios (Objective 5). Lastly, complexities with intoxicated patients and legal issues of involuntary and voluntary admission are investigated in cases 3 and 4B (Objectives 4 and 5).

### Recommended pre-reading for instructor

The instructor should review all material included in the TBL. Recommended reading for background information on the case includes:

1. Chang BP, Tezanos K, Gratch I, Cha C. Depressed and suicidal patients in the emergency department: an evidence-based approach. *Emerg Med Pract*. 2019;21(5):1–24.
2. Nazarian DJ. Clinical Policy: Critical Issues in the Diagnosis and Management of the Adult Psychiatric Patient in the Emergency Department. ACEP. Published January 17, 2017. Accessed September 13, 2021. At: https://www.acep.org/patient-care/clinical-policies/Psychiatric-Patient/
3. Betz ME, Boudreaux ED. Managing suicidal patients in the emergency department. *Annals of Emergency Medicine*. 2016;67(2):276–282. doi:10.1016/j.annemergmed.2015.09.001

### Learner Responsible Content (LRC)

Prior to the session, participants should review the following materials:

- Betz ME, Boudreaux ED. Managing suicidal patients in the emergency department. *Annals of Emergency Medicine*. 2016;67(2):276–282. At: doi:10.1016/j.annemergmed.2015.09.001
- Chang BP, Tezanos K, Gratch I, Cha C. Depressed and suicidal patients in the emergency department: an evidence-based approach. *Emerg Med Pract*. 2019;21(5):1–24.

Optional Reading for senior residents:

- Nazarian DJ. Clinical Policy: Critical Issues in the Diagnosis and Management of the Adult Psychiatric Patient in the Emergency Department. ACEP. Published January 17, 2017. Accessed September 13, 2021. AT: https://www.acep.org/patient-care/clinical-policies/Psychiatric-Patient/

### Results and tips for successful implementation

#### Results

The TBL session was conducted in person in fall 2021 at a tertiary academic center. The participants included emergency medicine residents post1-3 and emergency medicine-bound medical students. Groups consisted of five learners per group with a total of thirty-three participants. The session was evaluated using a post-activity evaluation using Likert scale questions. Overall, all the participants “strongly agreed” (Likert 5/5) or “agreed” (Likert 4/5) that the session improved their knowledge of caring for the suicidal patient in the emergency department for an average score of 4.6/5. All participants “strongly agreed” (Likert 5/5) or “agreed” (Likert 4/5) that the material presented was relevant to their clinical practice in the emergency department for an average score of 4.6/5. The learners felt that the LRC reinforced their knowledge of the material presented. The feedback that was included in the written comments was that “the [small group] discussion helped framed the material well.” One participant recommended the LRC be sent out prior to one week ahead for adequate time for preparation. Formative feedback from the faculty was that the activity was successful, and the TBL allowed for group interaction and critical thinking. Recommendations for future sessions include preassigning groups and allowing for more time for the GAE to allow for group discussion. Limitations to the TBL include the sample size and response rate from residents. Additionally, asking residents to evaluate their own educational activity could lead to undue influence on the results due to fear of providing negative feedback to program leaders.

#### Instructions for Implementation

One week prior to the session, the instructor should:

1. Send out the learner responsible content to the learners.
2. Send out the iRAT to the learners. We used Google Forms® to send out the questions.

Prior to the session, the instructor should prepare materials:

1. One gRAT per 3–5 learners per group.
2. One GAE per 3–5 learners per group.
3. Copies of all materials, including the keys for each instructor.

You will need approximately 50 mins to conduct the session.

We suggest the following timeline:

1. Introduce the session. (5 mins)
2. The instructor assigns learners into groups of 3–5. Ideally, groups will be a mix of learner levels. (5 mins)
3. Instructor hands out the gRAT to all groups of 3–5 learners. (5–10 mins)
4. Instructor review results of gRAT. (5 mins)
5. Groups will complete the GAE. Faculty should circulate to be able to answer questions as appropriate. (25 mins)
6. Review answers from GAE using the supplemental Microsoft PowerPoint. (10 mins)
7. Hand out the GAE-Key as a post-activity review guide.

## Supplementary Information











## Figures and Tables

**Figure f2-jetem-8-1-t1:**
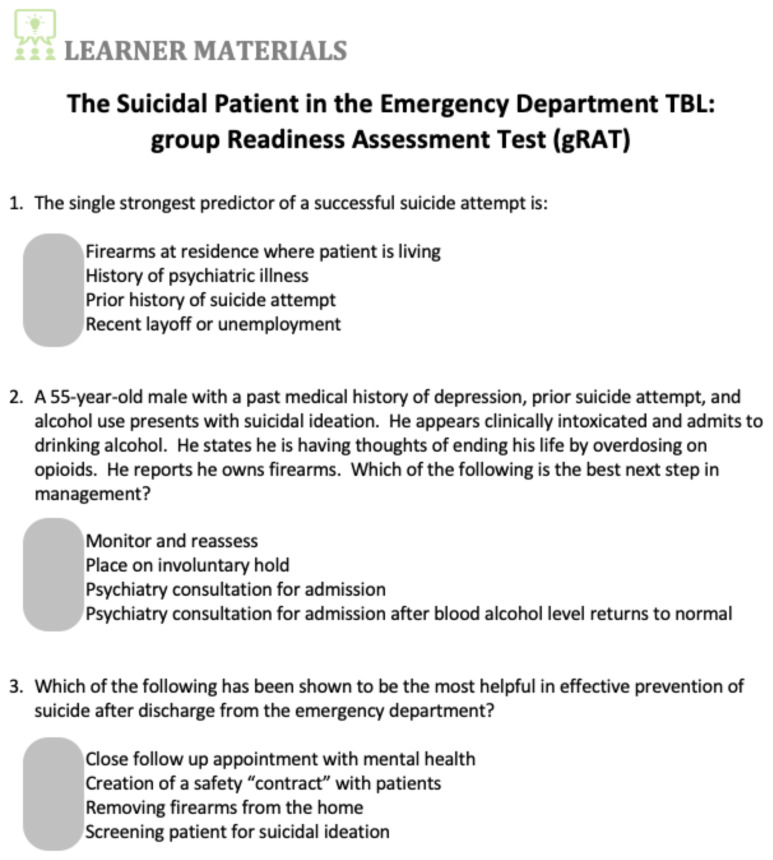


**Table t2-jetem-8-1-t1:** 

Toxidrome Physical Exam Findings:				
Toxidrome	Vital Signs	Pupils	Skin	Mental Status
Opioid				
Cholinergic				
Anticholinergic				
Sedative-Hypnotic				
Hallucinogenic				
Sympathomimetic				

**Table t3-jetem-8-1-t1:** 

Aspects of the Mental Status Evaluation	
Examination Component	Description
General Appearance	
Orientation	
Speech	
Motor Activity	
Affect	
Mood	
Thought Process	
Thought Content	
Perceptual Disturbances	

**Table t4-jetem-8-1-t1:** 

Modified SAD Persons Scale		
Factor		Points
S=	Sex (male)	1
A=	Age (<19 or >45 years)	1
D=	Depression or hopelessness	2
P=	Previous suicide attempts or psychiatric care	1
E=	Excessive alcohol or drug use	1
R=	Rational thinking loss	2
S=	Separated, divorced or widowed	1
O=	Organized or serious attempt	2
N=	No social supports	1
S=	Stated future intent	1

Hockberger RS, Rothstein RJ. Assessment of suicide potential by nonpsychiatrists using the SAD PERSONS score. *The Journal of emergency medicine*. 1988*;* 6(2), 99–107. https://doi.org/10.1016/0736-4679(88)90147-3

**Table t5-jetem-8-1-t1:** 

Toxidrome Physical Exam Findings				
Toxidrome	Vital Signs	Pupils	Skin	Mental Status
Opioid	↓Respiration Rate ↓Blood Pressure (BP)	Constricted	Normal	Depressed
Cholinergic	↓Heart Rate (HR)	Constricted	Diaphoresis	Depressed Confused Seizures
Anticholinergic	↑HR ↑BP ↑Temperature	Dilated	Dry	Depressed Confused Hallucinations Seizures
Sedative-Hypnotic	Normal	Normal	Normal	Depressed
Hallucinogenic	↑HR	Normal Dilated	Normal	Confused Hallucinations
Sympathomimetic	↑HR ↑BP ↑Temperature	Dilated	Diaphoresis	Alert Agitated Seizures

**Table t6-jetem-8-1-t1:** Aspects of the Mental Status Examination

Examination Component	Description
General Appearance	Patient’s general appearance including grooming, clothing, and posture
Orientation	Patient’s full name, full date (day, month, year), and current location
Speech	Note the patient’s speech including volume, rate, fluency, rhythm, tone, and spontaneity
Motor Activity	Patient’s motor behavior including gestures, tics, body movements, and gait
Affect	Patient’s outward projection of their emotional state. Examples include sad, depressed, anxious, agitated, irritable, angry, elated, expansive, labile, inappropriate, incongruent with mood, congruent
Mood	Patient’s stated mood. Present as a direct quote. For example, “I am anxious and have been feeling poorly over the past week.”
Thought Process	Comment on patient’s thinking. Is it logical, tangential, goal directed, flight of ideas, loosely associated?
Thought Content	Comment on suicidal ideation, homicidal ideations, delusion, repetitive themes
Perceptual Disturbances	Comment on auditory, olfactory, visual, and somatosensory hallucinations or delusions

Adapted from Chang BP, Tezanos K, Gratch I, Cha C. Depressed and suicidal patients in the emergency department: an evidence-based approach*. Emerg Med Pract*. 2019;21(5):1–24.

**Figure 1 f1-jetem-8-1-t1:**
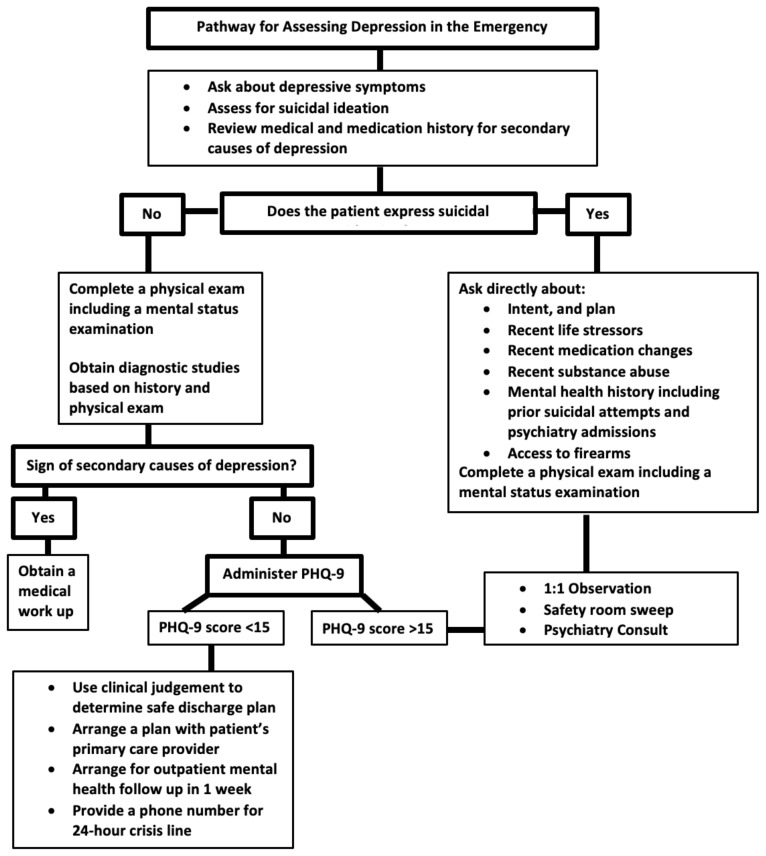
Adapted from Chang BP, Tezanos K, Gratch I, Cha C. Depressed and suicidal patients in the emergency department: an evidence-based approach*. Emerg Med Pract*. 2019;21(5):1–24*)*.

## LEARNER MATERIALS

### The Suicidal Patient in the Emergency Department TBL: individual Readiness Assessment Test (iRAT)

1. The single strongest predictor of a successful suicide attempt is:
  - Firearms at residence where patient is living
  - History of psychiatric illness
  - Prior history of suicide attempt
  - Recent layoff or unemployment
2. A 55-year-old male with a past medical history of depression, prior suicide attempt, and alcohol use presents with suicidal ideation. He appears clinically intoxicated and admits to drinking alcohol. He states he is having thoughts of ending his life by overdosing on opioids. He reports he owns firearms. Which of the following is the best next step in management?
  - Monitor and reassess
  - Place on involuntary hold
  - Psychiatry consultation for admission
  - Psychiatry consultation for admission after blood alcohol level returns to normal
3. Which of the following has been shown to be the most helpful in effective prevention of suicide after discharge from the emergency department?
  - Close follow up appointment with mental health
  - Creation of a safety “contract” with patients
  - Removing firearms from the home
  - Screening patient for suicidal ideation
4. Which statement is the most appropriate plan for patients who present with suicidal ideation?
  - Perform a physical exam, mental status evaluation, and discuss with a psychiatrist once medically cleared
  - May be discharged home after medical screening if they are established with an outpatient mental health clinician
  - All patients should be held involuntarily in the emergency department until psychiatric placement can be found
  - Patients should be immediately evaluated by a psychiatrist prior to medical screening by emergency medicine physician
5. Which of the following is TRUE for the emergency medicine physician when evaluating a patient with depression or suicidal ideations?
  - Review of systems should be focused on psychiatric symptoms
  - Routine serum and urine toxicology screening is recommended for all patients
  - Screening laboratory studies should be considered for new psychiatric symptoms in individuals > 65 years old
  - Screening computed tomography imaging of the brain should be obtained for new psychiatric symptoms in immunocompromised patients

### The Suicidal Patient in the Emergency Department TBL: group Readiness Assessment Test (gRAT)

Use scratch off stickers https://www.amazon.com/Kenco-Scratch-Off-Stickers-Silver/dp/B0839QF79D/ref=sr_1_8?dchild=1&keywords=scratch+off+stickers&qid=1586830370&sr=8-8 to hide the answers/stars so that learners get immediate feedback on their answer choice. See the example below.[Fig f2-jetem-8-1-t1]

### The Suicidal Patient in the Emergency Department TBL: group Readiness Assessment Test (gRAT)

1. The single strongest predictor of a successful suicide attempt is:
  a. Firearms at residence where patient is living
  b. History of psychiatric illness
  ⋆. Prior history of suicide attempt
  d. Recent layoff or unemployment
2. A 55-year-old male with a past medical history of depression, prior suicide attempt, and alcohol use presents with suicidal ideation. He appears clinically intoxicated and admits to drinking alcohol. He states he is having thoughts of ending his life by overdosing on opioids. He reports he owns firearms. Which of the following is the best next step in management?
  ⋆. Monitor and reassess
  b. Place on involuntary hold
  c. Psychiatry consultation for admission
  d. Psychiatry consultation for admission after blood alcohol level returns to normal
3. Which of the following has been shown to be the most helpful in effective prevention of suicide after discharge from the emergency department?
  a. Close follow up appointment with mental health
  b. Creation of a safety “contract” with patients
  ⋆. Removing firearms from the home
  d. Screening patient for suicidal ideation
4. Which statement is the most appropriate plan for patients who present with suicidal ideation?
  ⋆. Perform a physical exam, mental status evaluation, and discuss with a psychiatrist once medically cleared
  b. May be discharged home after medical screening if they are established with an outpatient mental health clinician
  c. All patients should be held involuntarily in the emergency department until psychiatric placement can be found
  d. Patients should be immediately evaluated by a psychiatrist prior to medical screening by emergency medicine physician
5. Which of the following is TRUE for the emergency medicine physician when evaluating a patient with depression or suicidal ideations?
  a. Review of systems should be focused on psychiatric symptoms
  b. Routine serum and urine toxicology screening is recommended for all patients
  ⋆. Screening laboratory studies should be considered for new psychiatric symptoms in individuals > 65 years old
  d. Screening computed tomography imaging of the brain should be obtained for new psychiatric symptoms in immunocompromised patients

### The Suicidal Patient in the Emergency Department TBL: Group Application Exercise (GAE)

#### Case #1

A 42yo M presents to the emergency department reporting suicidal thoughts with a plan. Patient states his brother made him come after patient texted him stating he wanted to “end it all.” Patient states he lost his job today and recently became divorced. Patient admits that he was thinking about suicide, but he would like to go home.

1. Define these terms or give an example of these different behaviors:
  - Passive suicidal thoughts –
  - Active suicidal thoughts –
  - Active suicidal thoughts with a plan –
  - Suicidal gesture -
2. Name some symptoms that a patient may complain of if they are experiencing depression.
3. What is the Diagnostic and Statistical Manual of Mental Disorders (DSM-5) Diagnostic Criteria for a major depressive episode?
4. What are risk factors for suicide?

#### Case #2**:**

28yo F is brought to the emergency department by emergency medical services (EMS) after suicidal attempt at home. Patient attempted suicide by self-injury with a razor blade.

1. Patient is brought back to a room in your emergency department. What are your initial steps to ensure patient safety?
2. What are your initial steps to evaluate the patient?
3. Patient appears to be reluctant to talk about today’s events with nursing staff. How would you engage the patient to open the conversation to obtain a history?

The patient tells you that she has been under a lot of stress lately. She has lost her social support after a recent divorce. She states due to recent missed shifts at work, she has lost her job. She reports no prior suicidal attempts or prior psychiatric admissions. Past medical history includes hypertension and takes Amlodipine 5MG daily for treatment

4. What signs on physical exam would make you concerned about possible ingestion?[Table t2-jetem-8-1-t1]
5. Describe your approach to your physical exam for this patient. What components are you including in your focused physical exam?

#### Case #3**:**

37yo M is brought by EMS after reporting suicidal ideation with a plan to a suicide hotline. Patient reports no new medication changes. Prior history of suicide attempt three years ago. Patient is here of his own volition. He states that he needs professional help for his suicidal thoughts. He states inpatient psychiatric admission has been helpful in the past.

1. To complete your examination of the patient, you completed a mental status evaluation of the patient. Please describe the different components of the mental status examination.[Table t3-jetem-8-1-t1]

The patient states he desires help. He feels that he is not going to be able to “fix” his depression and suicidal ideations on his own. He would like to be placed in an inpatient psych facility.

2. When would you consider involuntary placement versus voluntary placement?
3. Describe how you would explain an involuntary hold to your patient.
4. After your medical screening exam, mental status examination, and physical exam, you determine the patient is medically clear for psychiatric care. What are your next steps?

#### Case #4a**:**

27yo M presents to the emergency department after family found a suicide note. Patient endorses suicidal ideation with plan.

1. You have completed a full history and physical including mental status exam; you consider labs and other studies to work up this patient. What is the current American College of Emergency Physicians' (ACEP) policy on “screening studies?”
2. When would you consider the following studies:
  Computer Topography (CT) Head Imaging –
  Electrocardiogram (EKG) –
  Urine Drug Screen (UDS)/Acetaminophen/Alcohol/Salicylates –
  General Lab work (Complete Blood Count, Comprehensive Metabolic Panel, Thyroid Stimulating Hormone, etc)

#### Case #4b**:**

27yo M presents to the emergency department after family found a suicide note. Patient endorses suicidal ideation with plan. Patient appears acutely intoxicated. He endorses that he had a few beers prior to arrival. He states he usually drinks about 12 standard beers per day.

1. When is the best time for you to complete a mental status examination on the patient?
2. How will you determine the patient is clinically sober?
3. What is ACEP’s clinical policy recommendation for alcohol levels?

#### Case #5**:**

32yo F has been your care in the emergency department. Patient has had passive suicidal thoughts in the past but none currently. Patient has appropriate insight to her major depressive episode. She has been cleared by psychiatry for outpatient management for her depression. Patient feels comfortable with an outpatient management.

Please describe your discharge plan.

1. When and where should the patient follow up?
2. What would be helpful to include in your discharge instructions and after-visit summary?
3. What other instructions would you tell the patient prior to discharge?

The patient asks you about starting an antidepressant prior to discharge.

4. Would you consider starting an antidepressant?
5. What are some barriers to starting the medication in the emergency department?
6. Will an antidepressant help with the patient’s acute depressive symptoms?

## INSTRUCTOR MATERIALS

Answer keys to all exercises with explanations, are on the following pages.

Learners: Please do not proceed.

### The Suicidal Patient in the Emergency Department TBL: Readiness Assessment Test Key (RAT Key)

1. The single strongest predictor of suicide attempt is: (Objective 1)
  - Firearms at residence where patient is living
  - History of psychiatric illness
  - **Prior history of suicide attempt ***
  - Recent layoff or unemployment
  **Answer: (C) Individuals who have previously attempted suicide are six times more likely to make another attempt.****3** **One in 100 people who have attempted suicide will ultimately die of suicide within one year of initial suicide attempt.****3** **History of psychiatric illness increases risk by three times.****3** **Firearms are an independent risk factor but less of a risk than prior suicidal attempt.****3**
2. 55-year-old male with a past medical history of depression, prior suicide attempt, and alcohol use presents with suicidal ideation. He appears clinically intoxicated and admits to drinking alcohol. He states he is having thoughts of ending his life by overdose with opioids. He reports he owns firearms. Vital signs are within normal limits. Which of the following is the best next step in management? (Objective 6)
  - **Monitor and reassess ***
  - Place on involuntary hold
  - Psychiatry consultation for admission
  - Psychiatry consultation for admission after blood alcohol level returns to normal
  **Answer: (A) Alcohol abuse can worsen depression and depressive thoughts. Alcohol abuse is considered a risk factor for suicide. ****3** **Alcohol can complicate the approach to the suicidal patient in the emergency department (ED). Psychiatric evaluation is not recommended until no longer intoxicated. American College of Emergency Physicians (ACEP) recommends evaluating a patient’s cognitive abilities rather than a specific blood alcohol level as the guiding factor to pursue a formal psychiatric assessment.****1** **When clinically sober, all patients should be evaluated for psychiatric symptoms and resolution of symptoms. Patients without any ongoing safety risk may be managed in an outpatient setting**.
3. Which of the following has been shown to be the most helpful in effective prevention of suicide after discharge from the Emergency Department? (Objective 5)
  - Close follow-up appointment with mental health
  - Creation of a safety “contract” with patients
  - **Removing firearms from the home ***
  - Sending a post-discharge postcard
  **Answer: (C) Reducing access to lethal means (firearms) has been shown to reduce suicide attempts and deaths.****3** **Connecting with follow-up resources like mental is recommended but not effective at prevention. Safety contracts have not been shown to be helpful in randomized controlled trials in the prevention of suicide.****3** **A randomized controlled trial did show that sending postcards over the course of a year after being seen in the ED for suicidal ideation did reduce repeat suicide attempts but not to the same degree as reducing access to lethal means.****3**
4. Which statement is most appropriate for patients who present with suicidal ideation? (Objectives 2,3,4)
  - **Have a physical examination, mental status evaluation, and discussion with a psychiatric specialist ***
  - May be discharged home after medical screening if they are established with an outpatient mental health clinician
  - Should be held involuntarily in the emergency department until psychiatric placement can be found
  - Should be immediately evaluated by a psychiatric specialist prior to medical screening by emergency medicine physician
  **Answer: (A) All suicidal patients need a medical screening exam, physical exam, mental status evaluation, and discussion with a psychiatric specialist. A medical screening exam should occur before a psychiatric specialist evaluation. An involuntary hold may not be appropriate for all patients**.
5. Which of the following is TRUE for the emergency medicine physician when evaluating a patient with depression or suicidal ideations? (Objective 2)
  - Review of systems should be focused on psychiatric symptoms
  - Routine serum and urine toxicology screening is recommended
  - **Screening lab work should be considered for new psychiatric symptoms in individuals > 65 years old ***
  - Screening computer tomography (CT) imaging should be obtained for new psychiatric symptoms in patients >65yo
  **Answer: (C) Patients presenting to the ED with suicidal ideation should:**
  1. **Undergo a medical screening including a full medical and psychiatric history**
  2. **Undergo a focused physical examination**
  3. **Undergo a mental status examination**
  4. **Routine serum and urine toxicology screening is not recommended**

### The Suicidal Patient in the Emergency Department TBL: Group Application Exercise (GAE) Key

#### Case #1

A 42yo male presents to the emergency department (ED) reporting suicidal thoughts with a plan. Patient states his brother made him come after patient texted him stating he wanted to “end it all.” Patient states he lost his job today and recently became divorced. Patient admits that he was thinking about suicide, but he would like to go home.

1. Define these terms or give an example of these different behaviors.
  - Passive suicidal thoughts –
    **Person desires death, but does not have a specific plan. The patient may have a plan, but plan is out of their control. “I want to disappear,” or “The world is better off without me,” or “I hope I get hit by a bus.”**
  - Active suicidal thoughts –
    **May or may not have plan but actively wants to kill themselves. “I want to kill myself.”**
  - Active suicidal thoughts with a plan –
    **Actively wants to harm themselves and has a plan to do so. “I want to kill myself by overdose,” or “I want to die so I am going to walk across the road to be hit by a car.”**
  - Suicidal gesture -
    **An expressed statement or behavior that makes other people believe they want to kill themselves. The patient may not have clear suicidal intent. “Texting a family member stating I want to kill myself.”**
2. Name some symptoms that a patient may complain of if they are experiencing depression.
  1. **Depressed Mood**
  2. **Decreased Interest or pleasure**
  3. **Body Weight Change**
  4. **Insomnia or hypersomnia**
  5. **Psychomotor agitation, restlessness, or slowing of physical movement**
  6. **Fatigue or loss of energy**
  7. **Feeling of worthlessness or guilt**
  8. **Diminished ability to think or concentrate, or indecisiveness**
  9. **Recurrent thoughts of death or suicide**
3. What is the DSM-5 Diagnostic Criteria for a major depressive episode?
  **For diagnosis of major depressive disorder, at least one of the following symptoms is required. These symptoms must last most of the day, nearly every day, for a minimum of 2 weeks**.
  **Depression Symptoms:**
  1. **Depressed Mood**
  2. **Decreased Interest or pleasure**
  3. **Body Weight Change**
  4. **Insomnia or hypersomnia**
  5. **Psychomotor agitation, restlessness, or slowing of physical movement**
  6. **Fatigue or loss of energy**
  7. **Feeling of worthlessness or guilt**
  8. **Diminished ability to think or concentrate, or indecisiveness**
  9. **Recurrent thoughts of death or suicide**
4. What are risk factors for suicide?
  **SADPERSON scale is a mnemonic device that has been developed into a clinical assessment tool for medical professionals to determine suicide risk. However, the number of points should not affect your clinical assessment or disposition of the patient**.[Table t4-jetem-8-1-t1]

#### Case #2**:**

28yo female is brought to the emergency department by emergency medicine services (EMS) after suicidal attempt at home. Patient attempted suicide by self-injury with a razor blade.

1. Patient is brought back to a room in your emergency department. What are your initial steps to ensure patient safety?
  **The patient should be taken to an area of the ED that is free of all potentially dangerous medications and equipment. Patients should be searched for weapons, substances and other items of potential harm. They should be provided a set of scrubs or other disposable clothing to discourage elopement and encourage safety. Patients should not be left unattended. They should be observed in a 1:1 fashion with a designated sitter or another medical professional**.
2. What are your initial steps to evaluate the patient?
  **Obtain vital signs. If the patient is hemodynamically stable and life-threatening, overdose is likely not of concern**.
3. Patient appears to be reluctant to talk about today’s events with nursing staff. How would you engage the patient to open the conversation to obtain a history?
  **Try to maximize the patient’s privacy by interviewing in a private space. Especially avoid interviewing in the hallways. Approach the patient with an open-ended nonjudgmental question. Beginning with open-ended questions such as “What brought you here today?” or “How are you feeling?” Open-ended questions will elicit more detailed and helpful information**.
4. The patient tells you that she has been under a lot of stress lately. She has lost her social support after a recent divorce. She states due to recent missed shifts at work, she has lost her job. She reports no prior suicidal attempts or prior psychiatric admissions. Past medical history includes hypertension and takes amlodipine 5mg daily for treatment.
  **A review of symptoms incorporating infectious, toxic-metabolic, and neurologic complaints may help point to medical etiologies of the patient’s presentation. Also include recent medical events and medication changes**.
5. What signs on physical exam would make you concerned about possible ingestion?[Table t5-jetem-8-1-t1]
6. Describe your approach to your physical exam for this patient. What components are you including in your focused physical exam?
  **Physical examination should include vital signs, and head to toe physical exam. Actively search for signs and symptoms of acute ingestions, toxidromes, and withdrawal symptoms including diaphoresis, hyperthermia, bradypnea, miosis/mydriasis, hyper/hyporeflexia, tremors, clonus, and altered mental status**.

#### Case #3**:**

37yo male is brought by EMS after reporting suicidal ideation with a plan to a suicide hotline. Patient reports no new medication changes. Prior history of suicide attempt three years ago. Patient is here of his own volition. He states that he needs professional help for his suicidal thoughts. He states inpatient psychiatric admission has been helpful in the past.

1. To complete your examination of the patient you completed a mental status evaluation of the patient. Please describe the different components of the mental status examination.[Table t6-jetem-8-1-t1]

The patient states he desires help. He feels that he is not going to be able to “fix” his depression and suicidal ideations on his own. He would like to be placed in an inpatient psych facility.

2. When would you consider involuntary placement versus voluntary placement?
  **Involuntary confinement or hospitalization may be necessary for patients who are at imminent risk of harming themselves but refuse to stay in the ED or be hospitalized, or for suspected suicidal patients who refuse evaluation. In these cases, it may be necessary to hold patients involuntarily in the ED until a complete psychiatric and safety evaluation is performed and appropriate disposition planning has taken place. In general, an individual must be exhibiting behavior that is an imminent danger to himself or others, the hold must be for an evaluation only, and a court order must be received for more than a very short-term hospitalization. The timing of the holds and process is state dependent**.
  **Second, physicians must document their reasons supporting the decision to hold a patient involuntarily. Concerns for the patient’s safety should be explicitly documented, and/or the potential to harm himself and/or others**.
3. Describe how you would explain involuntary hold to your patient.
  **If the patient has an appropriate affect (not combative or paranoid, etc), tell the patient that they have an acute psychiatric condition that that requires further care. Tell the patient that they will need to stay in the ED to continue to receive care for their condition: “Due to our concerns for your safety, you will not be able to leave the facility, but you can still make decisions on your medical care. We will continue to observe you here in the ED until you are seen and cleared by the psychiatry team or receive admission to a psychiatric facility.”**
4. After your medical screening exam, mental status examination, and physical exam, you determine the patient is medically clear for psychiatric care. What are your next steps?
  **Consult a psychiatric team member for voluntary inpatient psychiatric placement of patient. Consider talking to family for collateral information. Continue 1:1 observation**.

#### Case #4a

27yo male presents to the emergency department after family found a suicide note. Patient endorses suicidal ideation with plan.

1. You have completed a full history and physical examination including mental status exam; you consider labs and other studies to work up this patient. What is the current ACEP policy on “screening studies?”
  **Screening studies including routine toxicology screening are not recommended per American College of Emergency Physicians (ACEP) guidelines.****1** **Data also does not support routine screening tests. Research has shown this increases door-to-disposition time and decreased time to inpatient psychiatric placement**.
2. When would you consider the following studies:
  Computer Topography (CT) Head Imaging
  **Should be considered in persistent altered mental status, abnormal neuro exam, or immunocompromised with new psych symptoms**.
  Electrocardiogram (EKG)
  **Consider if concerns for toxidromes, hemodynamically unstable, acutely agitated, or when repeat dosing (>2 doses) of QT prolongers are ordered**.
  Urine Drug Screen/Acetaminophen/Alcohol (EtOH)/Salicylates
  **Consider only if concerned about undifferentiated toxidromes. If the patient is awake and cooperative, ask them about ingestions instead**.
  Lab work (Complete Blood Count, Comprehensive Metabolic Panel, Thyroid Stimulating Hormone, etc)
  **Consider evaluating for new psychiatric symptoms in patients with a past medical history, >65yo or immunocompromised. Also, can consider Sexually Transmitted Infection labs (including syphilis and HIV screening) and infectious workup (chest x-ray, urine analysis, etc)**.

#### Case #4b

27yo male presents to the emergency department after family found a suicide note. Patient endorses suicidal ideation with plan. Patient appears acutely intoxicated. He endorses that he had a few beers prior to arrival. He states he usually drinks about 12 standard beers per day.

1. When is the best time for you to complete a mental status examination on the patient?
  **You will want to complete a mental status exam on the patient when the patient is clinically sober and able and willing to participate in the conversation**.
2. How will you determine if the patient is clinically sober?
  **Patient is able to speak in non-slurred speech. Patient is alert and oriented. Able to ambulate with a steady gait. Has fine motor skills intact. Able to articulate understanding and judgment on clinical decisions and plan**.
3. What is ACEP’s clinical policy recommendation for lab alcohol (EtOH) levels?
  **Recommendations are to watch and reassess the patient instead of placing the patient on an involuntary hold. You should reassess the patient for clinical sobriety. It is not recommended that you use EtOH levels to evaluate for sobriety. This can lead to accidental alcoholic withdrawals and delays in psychiatric care**.

#### Case #5

32yo female has been in your care in the emergency department. Patient has had passive suicidal thoughts in the past but none currently. Patient has appropriate insight to her major depressive episode. She has been cleared by psychiatry for outpatient management for her depression. The patient feels comfortable with outpatient management.

Please describe your discharge plan.

1. When and where should the patient follow up?
  **Patient should ideally follow up within 1 week of discharge from the ED with a mental health clinician**.
2. What would be helpful to include in your discharge summary?
  1. **Information on mental health clinicians in the community**
  2. **Information on 24-hour Crisis Line**
  3. **Medication Instructions**
  4. **Standard discharge instructions and return precautions**
3. What other instructions would you tell the patient prior to discharge?

**This can be achieved efficiently through 5 steps that can be taken in the ED** **3** **:**

1. **Provide a standard handout pamphlet that includes addresses, contact numbers, and information about insurance coverage of local outpatient mental health professionals**.
2. **Inform the patient of 24-hour crisis lines**.
3. **Ask the patient to anticipate any barriers to accessing preferred resources and what alternative solutions to barriers**.
4. **Schedule a follow-up appointment with a preferred outpatient clinician (ideally within one week of discharge)**.
5. **Document the patient’s preferred follow-up resources and steps taken to connect them with such resources**.

The patient asks you about starting an antidepressant prior to discharge.

4. Would you consider starting an antidepressant?
  **The administration of psychotropic medication to treat depression is not routinely initiated in the ED. If this is recommended by the psychiatry team, the ultimate choice of which specific antidepressant to use for patients should ideally be made in coordination with a psychiatry or primary care team following the patient because ongoing follow-up is essential for monitoring the efficacy and impact of any intervention**.
5. What are some barriers to starting the medication in the Emergency Department?
  **Close monitoring of the medication effectiveness and side effects is needed. Patient needs close outpatient follow-up. Ideally, the medication should be made in conjunction with psychiatry and primary care teams to consider the patient’s other medical conditions**.
6. Will an antidepressant help with the patient’s acute depressive symptoms?
  **Standard first-line antidepressants include selective serotonin reuptake inhibitors (SSRIs). SSRIs take 6–8 weeks to take effect and do not impact short-term symptoms. These medications will not help with the patient’s acute symptoms**.[Fig f1-jetem-8-1-t1]

### The Suicidal Patient in the Emergency Department TBL: Debrief PowerPoint

Please see associated PowerPoint file
