# Supplementary material for: The Suicidal Patient in the Emergency Department Team-Based Learning Activity
Source: J Educ Teach Emerg Med. 2023 Jan 31;8(1):T1–T37. doi: 10.21980/J8892X (PMC10332773; doi:10.21980/J8892X)
Supplement: Supplementary file 2 [file jetem-8-1-T1-supp2.docx]

**The Suicidal Patient in the Emergency Department TBL:**

**Readiness Assessment Test Key (RAT Key)**

1. The single strongest predictor of suicide attempt is: (Objective 1)
   1. Firearms at residence where patient is living
   2. History of psychiatric illness
   3. **Prior history of suicide attempt ***
   4. Recent layoff or unemployment

**Answer: (C) Individuals who have previously attempted suicide are six times more likely to make another attempt.^3^ One in 100 people who have attempted suicide will ultimately die of suicide within one year of initial suicide attempt.^3^ History of psychiatric illness increases risk by three times.^3^ Firearms are an independent risk factor but less of a risk than prior suicidal attempt.^3^**

1. 55-year-old male with a past medical history of depression, prior suicide attempt, and alcohol use presents with suicidal ideation. He appears clinically intoxicated and admits to drinking alcohol. He states he is having thoughts of ending his life by overdose with opioids. He reports he owns firearms. Vital signs are within normal limits. Which of the following is the best next step in management? (Objective 6)
2. **Monitor and reassess ***
3. Place on involuntary hold
4. Psychiatry consultation for admission
5. Psychiatry consultation for admission after blood alcohol level returns to normal

**Answer: (A) Alcohol abuse can worsen depression and depressive thoughts. Alcohol abuse is considered a risk factor for suicide. ^3^ Alcohol can complicate the approach to the suicidal patient in the emergency department (ED). Psychiatric evaluation is not recommended until no longer intoxicated. American College of Emergency Physicians (ACEP) recommends evaluating a patient’s cognitive abilities rather than a specific blood alcohol level as the guiding factor to pursue a formal psychiatric assessment.^1^ When clinically sober, all patients should be evaluated for psychiatric symptoms and resolution of symptoms. Patients without any ongoing safety risk may be managed in an outpatient setting.**

1. Which of the following has been shown to be the most helpful in effective prevention of suicide after discharge from the Emergency Department? (Objective 5)
2. Close follow-up appointment with mental health
3. Creation of a safety “contract” with patients
4. **Removing firearms from the home ***
5. Sending a post-discharge postcard

**Answer: (C) Reducing access to lethal means (firearms) has been shown to reduce suicide attempts and deaths.^3^ Connecting with follow-up resources like mental is recommended but not effective at prevention. Safety contracts have not been shown to be helpful in randomized controlled trials in the prevention of suicide.^3^ A randomized controlled trial did show that sending postcards over the course of a year after being seen in the ED for suicidal ideation did reduce repeat suicide attempts but not to the same degree as reducing access to lethal means.^3^**

1. Which statement is most appropriate for patients who present with suicidal ideation? (Objectives 2,3,4)
2. **Have a physical examination, mental status evaluation, and discussion with a psychiatric specialist ***
3. May be discharged home after medical screening if they are established with an outpatient mental health clinician
4. Should be held involuntarily in the emergency department until psychiatric placement can be found
5. Should be immediately evaluated by a psychiatric specialist prior to medical screening by emergency medicine physician

**Answer: (A) All suicidal patients need a medical screening exam, physical exam, mental status evaluation, and discussion with a psychiatric specialist. A medical screening exam should occur before a psychiatric specialist evaluation. An involuntary hold may not be appropriate for all patients.**

1. Which of the following is TRUE for the emergency medicine physician when evaluating a patient with depression or suicidal ideations? (Objective 2)
2. Review of systems should be focused on psychiatric symptoms
3. Routine serum and urine toxicology screening is recommended
4. **Screening lab work should be considered for new psychiatric symptoms in individuals > 65 years old ***
5. Screening computer tomography (CT) imaging should be obtained for new psychiatric symptoms in patients >65yo

**Answer: (C) Patients presenting to the ED with suicidal ideation should:**

1. **Undergo a medical screening including a full medical and psychiatric history**
2. **Undergo a focused physical examination**
3. **Undergo a mental status examination**
4. **Routine serum and urine toxicology screening is not recommended**

**References:**

1. Chang BP, Tezanos K, Gratch I, Cha C. Depressed and suicidal patients in the emergency department: an evidence-based approach. *Emerg Med Pract*. 2019;21(5):1-24.
2. Nazarian DJ. Clinical Policy: Critical Issues in the Diagnosis and Management of the Adult Psychiatric Patient in the Emergency Department. ACEP . Published January 17, 2017. Accessed September 13, 2021. At: https://www.acep.org/patient-care/clinical-policies/Psychiatric-Patient/
3. Betz ME, Boudreaux ED. Managing suicidal patients in the emergency department. *Annals of Emergency Medicine*. 2016;67(2):276-282. At: doi:10.1016/j.annemergmed.2015.09.001
4. Hockberger RS, Rothstein RJ. Assessment of suicide potential by nonpsychiatrists using the sad persons score. *The Journal of Emergency Medicine*. 1988;6(2):99-107. At: doi:10.1016/0736-4679(88)90147-3
5. DeVos E. Suicidal. CDEM Curriculum: Suicidal. Published 2019. Accessed September 13, 2021. At: https://www.saem.org/about-saem/academies-interest-groups-affiliates2/cdem/for-students/online-education/m4-curriculum/group-m4-psychiatry/suicidal
6. Smith E. Emergency department tips & tricks for managing the suicidal patient. emDOCs.net - Emergency Medicine Education. Published April 21, 2017. Accessed September 13, 2021. At: http://www.emdocs.net/emergency-department-tips-tricks-managing-suicidal-patient/
