# Supplementary material for: The Suicidal Patient in the Emergency Department Team-Based Learning Activity
Source: J Educ Teach Emerg Med. 2023 Jan 31;8(1):T1–T37. doi: 10.21980/J8892X (PMC10332773; doi:10.21980/J8892X)
Supplement: Supplementary file 3 [file jetem-8-1-T1-supp3.docx]

**The Suicidal Patient in the Emergency Department TBL:**

**group Readiness Assessment Test (gRAT)**

1. The single strongest predictor of a successful suicide attempt is:

- 1. Firearms at residence where patient is living
  2. History of psychiatric illness
  3. Prior history of suicide attempt
  4. Recent layoff or unemployment

1. A 55-year-old male with a past medical history of depression, prior suicide attempt, and alcohol use presents with suicidal ideation. He appears clinically intoxicated and admits to drinking alcohol. He states he is having thoughts of ending his life by overdosing on opioids. He reports he owns firearms. Which of the following is the best next step in management?
   1. Monitor and reassess
   2. Place on involuntary hold
   3. Psychiatry consultation for admission
   4. Psychiatry consultation for admission after blood alcohol level returns to normal
2. Which of the following has been shown to be the most helpful in effective prevention of suicide after discharge from the emergency department?
   1. Close follow up appointment with mental health
   2. Creation of a safety “contract” with patients
   3. Removing firearms from the home
   4. Screening patient for suicidal ideation
3. Which statement is the most appropriate plan for patients who present with suicidal ideation?
   1. Perform a physical exam, mental status evaluation, and discuss with a psychiatrist once medically cleared
   2. May be discharged home after medical screening if they are established with an outpatient mental health clinician
   3. All patients should be held involuntarily in the emergency department until psychiatric placement can be found
   4. Patients should be immediately evaluated by a psychiatrist prior to medical screening by emergency medicine physician
4. Which of the following is TRUE for the emergency medicine physician when evaluating a patient with depression or suicidal ideations?
   1. Review of systems should be focused on psychiatric symptoms
   2. Routine serum and urine toxicology screening is recommended for all patients
   3. Screening laboratory studies should be considered for new psychiatric symptoms in individuals > 65 years old
   4. Screening computed tomography imaging of the brain should be obtained for new psychiatric symptoms in immunocompromised patients
