# Supplementary material for: The Suicidal Patient in the Emergency Department Team-Based Learning Activity
Source: J Educ Teach Emerg Med. 2023 Jan 31;8(1):T1–T37. doi: 10.21980/J8892X (PMC10332773; doi:10.21980/J8892X)
Supplement: Supplementary file 4 [file jetem-8-1-T1-supp4.docx]

**The Suicidal Patient in the Emergency Department TBL:**

**Group Application Exercise (GAE) Key**

***Case #1***:

A 42yo male presents to the emergency department (ED) reporting suicidal thoughts with a plan. Patient states his brother made him come after patient texted him stating he wanted to “end it all.” Patient states he lost his job today and recently became divorced. Patient admits that he was thinking about suicide, but he would like to go home.

1. Define these terms or give an example of these different behaviors.
   1. Passive suicidal thoughts –

**Person desires death, but does not have a specific plan. The patient may have a plan, but plan is out of their control. “I want to disappear,” or “The world is better off without me,” or “I hope I get hit by a bus.”**

- 1. Active suicidal thoughts –

**May or may not have plan but actively wants to kill themselves. “I want to kill myself.”**

- 1. Active suicidal thoughts with a plan –

**Actively wants to harm themselves and has a plan to do so. “I want to kill myself by overdose,” or “I want to die so I am going to walk across the road to be hit by a car.”**

- 1. Suicidal gesture -

**An expressed statement or behavior that makes other people believe they want to kill themselves. The patient may not have clear suicidal intent. “Texting a family member stating I want to kill myself.”**

1. Name some symptoms that a patient may complain of if they are experiencing depression.
2. **Depressed Mood**
3. **Decreased Interest or pleasure**
4. **Body Weight Change**
5. **Insomnia or hypersomnia**
6. **Psychomotor agitation, restlessness, or slowing of physical movement**
7. **Fatigue or loss of energy**
8. **Feeling of worthlessness or guilt**
9. **Diminished ability to think or concentrate, or indecisiveness**
10. **Recurrent thoughts of death or suicide**
11. What is the DSM-5 Diagnostic Criteria for a major depressive episode?

**For diagnosis of major depressive disorder, at least one of the following symptoms is required. These symptoms must last most of the day, nearly every day, for a minimum of 2 weeks.**

**Depression Symptoms:**

1. **Depressed Mood**
2. **Decreased Interest or pleasure**
3. **Body Weight Change**
4. **Insomnia or hypersomnia**
5. **Psychomotor agitation, restlessness, or slowing of physical movement**
6. **Fatigue or loss of energy**
7. **Feeling of worthlessness or guilt**
8. **Diminished ability to think or concentrate, or indecisiveness**
9. **Recurrent thoughts of death or suicide**
10. What are risk factors for suicide?

**SADPERSON scale is a mnemonic device that has been developed into a clinical assessment tool for medical professionals to determine suicide risk. However, the number of points should not affect your clinical assessment or disposition of the patient.**

| **Modified SAD Persons Scale** | | |
| --- | --- | --- |
| **Factor** | | **Points** |
| **S=** | **Sex (male)** | **1** |
| **A=** | **Age (<19 or >45 years)** | **1** |
| **D=** | **Depression or hopelessness** | **2** |
| **P=** | **Previous suicide attempts or psychiatric care** | **1** |
| **E=** | **Excessive alcohol or drug use** | **1** |
| **R=** | **Rational thinking loss** | **2** |
| **S=** | **Separated, divorced or widowed** | **1** |
| **O=** | **Organized or serious attempt** | **2** |
| **N=** | **No social supports** | **1** |
| **S=** | **Stated future intent** | **1** |

Hockberger RS, Rothstein RJ. Assessment of suicide potential by nonpsychiatrists using the SAD PERSONS score. The Journal of emergency medicine. 1988; 6(2), 99–107. https://doi.org/10.1016/0736-4679(88)90147-3

***Case #2*:**

28yo female is brought to the emergency department by emergency medicine services (EMS) after suicidal attempt at home. Patient attempted suicide by self-injury with a razor blade.

1. Patient is brought back to a room in your emergency department. What are your initial steps to ensure patient safety?

**The patient should be taken to an area of the ED that is free of all potentially dangerous medications and equipment. Patients should be searched for weapons, substances and other items of potential harm. They should be provided a set of scrubs or other disposable clothing to discourage elopement and encourage safety. Patients should not be left unattended. They should be observed in a 1:1 fashion with a designated sitter or another medical professional.**

1. What are your initial steps to evaluate the patient?

**Obtain vital signs. If the patient is hemodynamically stable and life-threatening, overdose is likely not of concern.**

1. Patient appears to be reluctant to talk about today’s events with nursing staff. How would you engage the patient to open the conversation to obtain a history?

**Try to maximize the patient’s privacy by interviewing in a private space. Especially avoid interviewing in the hallways. Approach the patient with an open-ended nonjudgmental question. Beginning with open-ended questions such as “What brought you here today?” or “How are you feeling?” Open-ended questions will elicit more detailed and helpful information.**

1. The patient tells you that she has been under a lot of stress lately. She has lost her social support after a recent divorce. She states due to recent missed shifts at work, she has lost her job. She reports no prior suicidal attempts or prior psychiatric admissions. Past medical history includes hypertension and takes amlodipine 5mg daily for treatment.

**A review of symptoms incorporating infectious, toxic-metabolic, and neurologic complaints may help point to medical etiologies of the patient’s presentation. Also include recent medical events and medication changes.**

1. What signs on physical exam would make you concerned about possible ingestion?

**Toxidrome Physical Exam Findings**

| **Toxidrome** | **Vital Signs** | **Pupils** | **Skin** | **Mental Status** |
| --- | --- | --- | --- | --- |
| **Opioid** | **↓Respiration Rate**  **↓Blood Pressure (BP)** | **Constricted** | **Normal** | **Depressed** |
| **Cholinergic** | **↓Heart Rate (HR)** | **Constricted** | **Diaphoresis** | **Depressed**  **Confused**  **Seizures** |
| **Anticholinergic** | **↑HR**  **↑BP**  **↑Temperature** | **Dilated** | **Dry** | **Depressed**  **Confused**  **Hallucinations**  **Seizures** |
| **Sedative-Hypnotic** | **Normal** | **Normal** | **Normal** | **Depressed** |
| **Hallucinogenic** | **↑HR** | **Normal**  **Dilated** | **Normal** | **Confused**  **Hallucinations** |
| **Sympathomimetic** | **↑HR**  **↑BP**  **↑Temperature** | **Dilated** | **Diaphoresis** | **Alert**  **Agitated**  **Seizures** |

1. Describe your approach to your physical exam for this patient. What components are you including in your focused physical exam?

**Physical examination should include vital signs, and head to toe physical exam. Actively search for signs and symptoms of acute ingestions, toxidromes, and withdrawal symptoms including diaphoresis, hyperthermia, bradypnea, miosis/mydriasis, hyper/hyporeflexia, tremors, clonus, and altered mental status.**

***Case #3*:**

37yo male is brought by EMS after reporting suicidal ideation with a plan to a suicide hotline. Patient reports no new medication changes. Prior history of suicide attempt three years ago. Patient is here of his own volition. He states that he needs professional help for his suicidal thoughts. He states inpatient psychiatric admission has been helpful in the past.

1. To complete your examination of the patient you completed a mental status evaluation of the patient. Please describe the different components of the mental status examination.

**Aspects of the Mental Status Examination**

| **Examination Component** | **Description** |
| --- | --- |
| **General Appearance** | **Patient’s general appearance including grooming, clothing, and posture** |
| **Orientation** | **Patient’s full name, full date (day, month, year), and current location** |
| **Speech** | **Note the patient’s speech including volume, rate, fluency, rhythm, tone, and spontaneity** |
| **Motor Activity** | **Patient’s motor behavior including gestures, tics, body movements, and gait** |
| **Affect** | **Patient’s outward projection of their emotional state. Examples include sad, depressed, anxious, agitated, irritable, angry, elated, expansive, labile, inappropriate, incongruent with mood, congruent** |
| **Mood** | **Patient’s stated mood. Present as a direct quote. For example, “I am anxious and have been feeling poorly over the past week.”** |
| **Thought Process** | **Comment on patient’s thinking. Is it logical, tangential, goal directed, flight of ideas, loosely associated?** |
| **Thought Content** | **Comment on suicidal ideation, homicidal ideations, delusion, repetitive themes** |
| **Perceptual Disturbances** | **Comment on auditory, olfactory, visual, and somatosensory hallucinations or delusions** |

Adapted from Chang BP, Tezanos K, Gratch I, Cha C. Depressed and suicidal patients in the emergency department: an evidence-based approach. Emerg Med Pract. 2019;21(5):1-24.

The patient states he desires help. He feels that he is not going to be able to “fix” his depression and suicidal ideations on his own. He would like to be placed in an inpatient psych facility.

1. When would you consider involuntary placement versus voluntary placement?

**Involuntary confinement or hospitalization may be necessary for patients who are at imminent risk of harming themselves but refuse to stay in the ED or be hospitalized, or for suspected suicidal patients who refuse evaluation. In these cases, it may be necessary to hold patients involuntarily in the ED until a complete psychiatric and safety evaluation is performed and appropriate disposition planning has taken place. In general, an individual must be exhibiting behavior that is an imminent danger to himself or others, the hold must be for an evaluation only, and a court order must be received for more than a very short-term hospitalization. The timing of the holds and process is state dependent.**

**Second, physicians must document their reasons supporting the decision to hold a patient involuntarily. Concerns for the patient’s safety should be explicitly documented, and/or the potential to harm himself and/or others.**

1. Describe how you would explain involuntary hold to your patient.

**If the patient has an appropriate affect (not combative or paranoid, etc), tell the patient that they have an acute psychiatric condition that that requires further care. Tell the patient that they will need to stay in the ED to continue to receive care for their condition: “Due to our concerns for your safety, you will not be able to leave the facility, but you can still make decisions on your medical care. We will continue to observe you here in the ED until you are seen and cleared by the psychiatry team or receive admission to a psychiatric facility.”**

1. After your medical screening exam, mental status examination, and physical exam, you determine the patient is medically clear for psychiatric care. What are your next steps?

**Consult a psychiatric team member for voluntary inpatient psychiatric placement of patient. Consider talking to family for collateral information. Continue 1:1 observation.**

***Case #4a:***

27yo male presents to the emergency department after family found a suicide note. Patient endorses suicidal ideation with plan.

1. You have completed a full history and physical examination including mental status exam; you consider labs and other studies to work up this patient. What is the current ACEP policy on “screening studies?”

**Screening studies including routine toxicology screening are not recommended per American College of Emergency Physicians (ACEP) guidelines.^1^ Data also does not support routine screening tests. Research has shown this increases door-to-disposition time and decreased time to inpatient psychiatric placement.**

1. When would you consider the following studies:

Computer Topography (CT) Head Imaging

**Should be considered in persistent altered mental status, abnormal neuro exam, or immunocompromised with new psych symptoms.**

Electrocardiogram (EKG)

**Consider if concerns for toxidromes, hemodynamically unstable, acutely agitated, or when repeat dosing (>2 doses) of QT prolongers are ordered.**

Urine Drug Screen/Acetaminophen/Alcohol (EtOH)/Salicylates

**Consider only if concerned about undifferentiated toxidromes. If the patient is awake and cooperative, ask them about ingestions instead.**

Lab work (Complete Blood Count, Comprehensive Metabolic Panel, Thyroid Stimulating Hormone, etc)

**Consider evaluating for new psychiatric symptoms in patients with a past medical history, >65yo or immunocompromised. Also, can consider Sexually Transmitted Infection labs (including syphilis and HIV screening) and infectious workup (chest x-ray, urine analysis, etc).**

**Case #4b:**

27yo male presents to the emergency department after family found a suicide note. Patient endorses suicidal ideation with plan. Patient appears acutely intoxicated. He endorses that he had a few beers prior to arrival. He states he usually drinks about 12 standard beers per day.

1. When is the best time for you to complete a mental status examination on the patient?

**You will want to complete a mental status exam on the patient when the patient is clinically sober and able and willing to participate in the conversation.**

1. How will you determine if the patient is clinically sober?

**Patient is able to speak in non-slurred speech. Patient is alert and oriented. Able to ambulate with a steady gait. Has fine motor skills intact. Able to articulate understanding and judgment on clinical decisions and plan.**

1. What is ACEP’s clinical policy recommendation for lab alcohol (EtOH) levels?

**Recommendations are to watch and reassess the patient instead of placing the patient on an involuntary hold. You should reassess the patient for clinical sobriety. It is not recommended that you use EtOH levels to evaluate for sobriety. This can lead to accidental alcoholic withdrawals and delays in psychiatric care.**

***Case #5:***

32yo female has been in your care in the emergency department. Patient has had passive suicidal thoughts in the past but none currently. Patient has appropriate insight to her major depressive episode. She has been cleared by psychiatry for outpatient management for her depression. The patient feels comfortable with outpatient management.

Please describe your discharge plan.

1. When and where should the patient follow up?

**Patient should ideally follow up within 1 week of discharge from the ED with a mental health clinician.**

1. What would be helpful to include in your discharge summary?

**1. Information on mental health clinicians in the community**

**2. Information on 24-hour Crisis Line**

**3. Medication Instructions**

**4. Standard discharge instructions and return precautions**

1. What other instructions would you tell the patient prior to discharge?

**This can be achieved efficiently through 5 steps that can be taken in the ED^3^:**

1. **Provide a standard handout pamphlet that includes addresses, contact numbers, and information about insurance coverage of local outpatient mental health professionals.**
2. **Inform the patient of 24-hour crisis lines.**
3. **Ask the patient to anticipate any barriers to accessing preferred resources and what alternative solutions to barriers.**
4. **Schedule a follow-up appointment with a preferred outpatient clinician (ideally within one week of discharge).**
5. **Document the patient’s preferred follow-up resources and steps taken to connect them with such resources.**

The patient asks you about starting an antidepressant prior to discharge.

1. Would you consider starting an antidepressant?

**The administration of psychotropic medication to treat depression is not routinely initiated in the ED. If this is recommended by the psychiatry team, the ultimate choice of which specific antidepressant to use for patients should ideally be made in coordination with a psychiatry or primary care team following the patient because ongoing follow-up is essential for monitoring the efficacy and impact of any intervention.**

1. What are some barriers to starting the medication in the Emergency Department?

**Close monitoring of the medication effectiveness and side effects is needed. Patient needs close outpatient follow-up. Ideally, the medication should be made in conjunction with psychiatry and primary care teams to consider the patient’s other medical conditions.**

1. Will an antidepressant help with the patient’s acute depressive symptoms?

**Standard first-line antidepressants include selective serotonin reuptake inhibitors (SSRIs). SSRIs take 6-8 weeks to take effect and do not impact short-term symptoms. These medications will not help with the patient’s acute symptoms.**


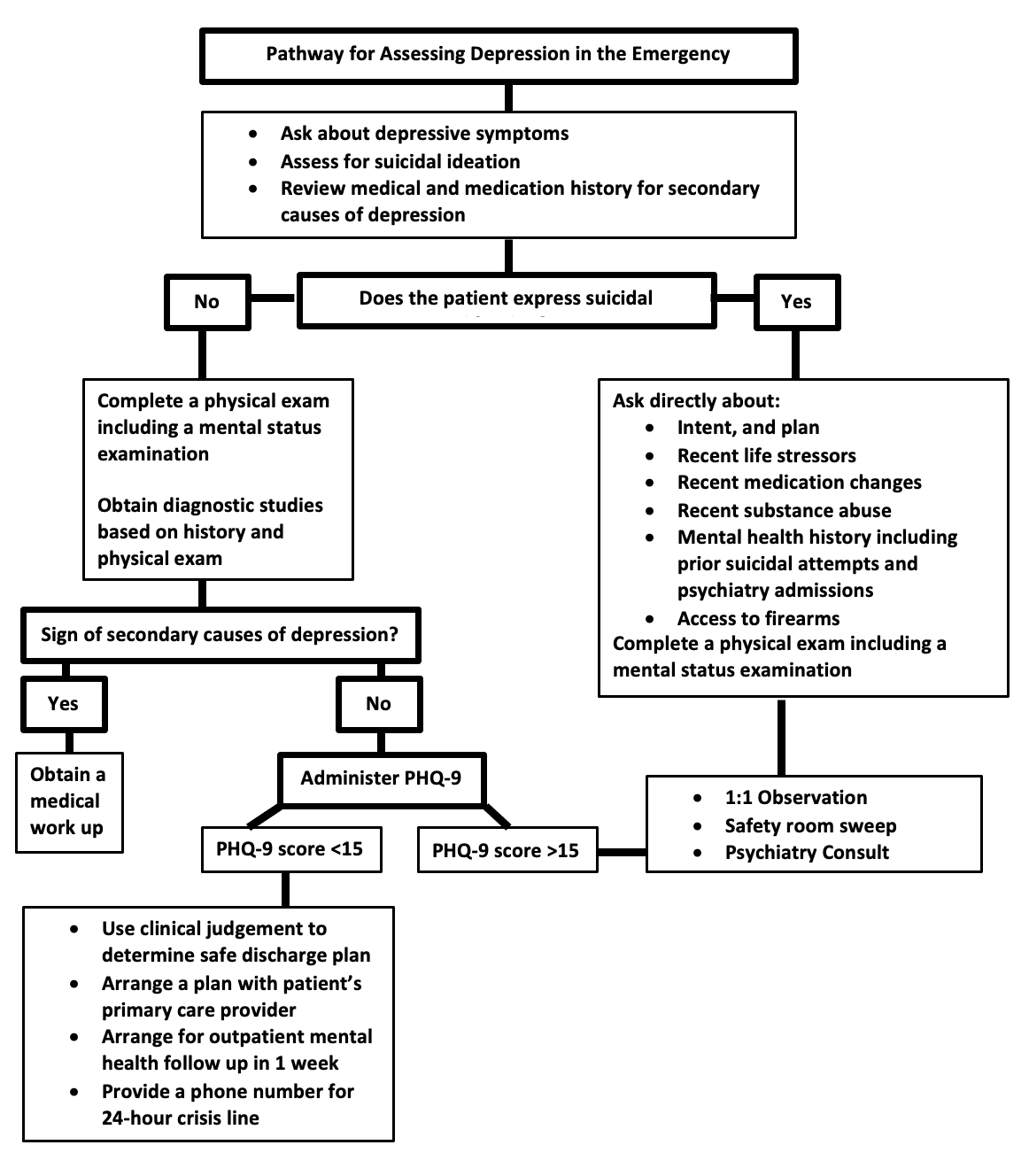


Figure 1: Adapted from Chang BP, Tezanos K, Gratch I, Cha C. Depressed and suicidal patients in the emergency department: an evidence-based approach. Emerg Med Pract. 2019;21(5):1-24).

**References:**

1. Chang BP, Tezanos K, Gratch I, Cha C. Depressed and suicidal patients in the emergency department: an evidence-based approach. *Emerg Med Pract*. 2019;21(5):1-24.
2. Nazarian DJ. Clinical Policy: Critical Issues in the Diagnosis and Management of the Adult Psychiatric Patient in the Emergency Department. ACEP . Published January 17, 2017. Accessed September 13, 2021. At: https://www.acep.org/patient-care/clinical-policies/Psychiatric-Patient/
3. Betz ME, Boudreaux ED. Managing suicidal patients in the emergency department. *Annals of Emergency Medicine*. 2016;67(2):276-282. At: doi:10.1016/j.annemergmed.2015.09.001
4. Hockberger RS, Rothstein RJ. Assessment of suicide potential by nonpsychiatrists using the sad persons score. *The Journal of Emergency Medicine*. 1988;6(2):99-107. At: doi:10.1016/0736-4679(88)90147-3
5. DeVos E. Suicidal. CDEM Curriculum: Suicidal. Published 2019. Accessed September 13, 2021. At: https://www.saem.org/about-saem/academies-interest-groups-affiliates2/cdem/for-students/online-education/m4-curriculum/group-m4-psychiatry/suicidal
6. Smith E. Emergency department tips & tricks for managing the suicidal patient. emDOCs.net - Emergency Medicine Education. Published April 21, 2017. Accessed September 13, 2021. At: http://www.emdocs.net/emergency-department-tips-tricks-managing-suicidal-patient/
