# Supplementary material for: The Suicidal Patient in the Emergency Department Team-Based Learning Activity
Source: J Educ Teach Emerg Med. 2023 Jan 31;8(1):T1–T37. doi: 10.21980/J8892X (PMC10332773; doi:10.21980/J8892X)
Supplement: Supplementary file 5 [file jetem-8-1-T1-supp5.docx]

**The Suicidal Patient in the Emergency Department TBL:**

**Group Application Exercise (GAE)**

***Case #1***:

A 42yo M presents to the emergency department reporting suicidal thoughts with a plan. Patient states his brother made him come after patient texted him stating he wanted to “end it all.” Patient states he lost his job today and recently became divorced. Patient admits that he was thinking about suicide, but he would like to go home.

1. Define these terms or give an example of these different behaviors:
   1. Passive suicidal thoughts –
   2. Active suicidal thoughts –
   3. Active suicidal thoughts with a plan –
   4. Suicidal gesture -
2. Name some symptoms that a patient may complain of if they are experiencing depression.
3. What is the Diagnostic and Statistical Manual of Mental Disorders (DSM-5) Diagnostic Criteria for a major depressive episode?
4. What are risk factors for suicide?

***Case #2*:**

28yo F is brought to the emergency department by emergency medical services (EMS) after suicidal attempt at home. Patient attempted suicide by self-injury with a razor blade.

1. Patient is brought back to a room in your emergency department. What are your initial steps to ensure patient safety?
2. What are your initial steps to evaluate the patient?
3. Patient appears to be reluctant to talk about today’s events with nursing staff. How would you engage the patient to open the conversation to obtain a history?

The patient tells you that she has been under a lot of stress lately. She has lost her social support after a recent divorce. She states due to recent missed shifts at work, she has lost her job. She reports no prior suicidal attempts or prior psychiatric admissions. Past medical history includes hypertension and takes Amlodipine 5MG daily for treatment

1. What signs on physical exam would make you concerned about possible ingestion?

**Toxidrome Physical Exam Findings:**

| **Toxidrome** | **Vital Signs** | **Pupils** | **Skin** | **Mental Status** |
| --- | --- | --- | --- | --- |
| **Opioid** |  |  |  |  |
| **Cholinergic** |  |  |  |  |
| **Anticholinergic** |  |  |  |  |
| **Sedative-Hypnotic** |  |  |  |  |
| **Hallucinogenic** |  |  |  |  |
| **Sympathomimetic** |  |  |  |  |

1. Describe your approach to your physical exam for this patient. What components are you including in your focused physical exam?

***Case #3*:**

37yo M is brought by EMS after reporting suicidal ideation with a plan to a suicide hotline. Patient reports no new medication changes. Prior history of suicide attempt three years ago. Patient is here of his own volition. He states that he needs professional help for his suicidal thoughts. He states inpatient psychiatric admission has been helpful in the past.

1. To complete your examination of the patient, you completed a mental status evaluation of the patient. Please describe the different components of the mental status examination.

**Aspects of the Mental Status Evaluation**

| **Examination Component** | **Description** |
| --- | --- |
| **General Appearance** |  |
| **Orientation** |  |
| **Speech** |  |
| **Motor Activity** |  |
| **Affect** |  |
| **Mood** |  |
| **Thought Process** |  |
| **Thought Content** |  |
| **Perceptual Disturbances** |  |

The patient states he desires help. He feels that he is not going to be able to “fix” his depression and suicidal ideations on his own. He would like to be placed in an inpatient psych facility.

1. When would you consider involuntary placement versus voluntary placement?
2. Describe how you would explain an involuntary hold to your patient.
3. After your medical screening exam, mental status examination, and physical exam, you determine the patient is medically clear for psychiatric care. What are your next steps?

***Case #4a*:**

27yo M presents to the emergency department after family found a suicide note. Patient endorses suicidal ideation with plan.

1. You have completed a full history and physical including mental status exam; you consider labs and other studies to work up this patient. What is the current American College of Emergency Physicians' (ACEP) policy on “screening studies?”
2. When would you consider the following studies:

Computer Topography (CT) Head Imaging –

Electrocardiogram (EKG) –

Urine Drug Screen (UDS)/Acetaminophen/Alcohol/Salicylates –

General Lab work (Complete Blood Count, Comprehensive Metabolic Panel, Thyroid Stimulating Hormone, etc)

***Case #4b*:**

27yo M presents to the emergency department after family found a suicide note. Patient endorses suicidal ideation with plan. Patient appears acutely intoxicated. He endorses that he had a few beers prior to arrival. He states he usually drinks about 12 standard beers per day.

1. When is the best time for you to complete a mental status examination on the patient?
2. How will you determine the patient is clinically sober?
3. What is ACEP’s clinical policy recommendation for alcohol levels?

***Case #5*:**

32yo F has been your care in the emergency department. Patient has had passive suicidal thoughts in the past but none currently. Patient has appropriate insight to her major depressive episode. She has been cleared by psychiatry for outpatient management for her depression. Patient feels comfortable with an outpatient management.

Please describe your discharge plan.

1. When and where should the patient follow up?
2. What would be helpful to include in your discharge instructions and after-visit summary?
3. What other instructions would you tell the patient prior to discharge?

The patient asks you about starting an antidepressant prior to discharge.

1. Would you consider starting an antidepressant?
2. What are some barriers to starting the medication in the emergency department?
3. Will an antidepressant help with the patient’s acute depressive symptoms?
