# Supplementary material for: The Suicidal Patient in the Emergency Department Team-Based Learning Activity
Source: J Educ Teach Emerg Med. 2023 Jan 31;8(1):T1–T37. doi: 10.21980/J8892X (PMC10332773; doi:10.21980/J8892X)
Supplement: Supplementary file 6 — Please see associated PowerPoint file [file jetem-8-1-t1-supp6.pptx]

## Slide 1
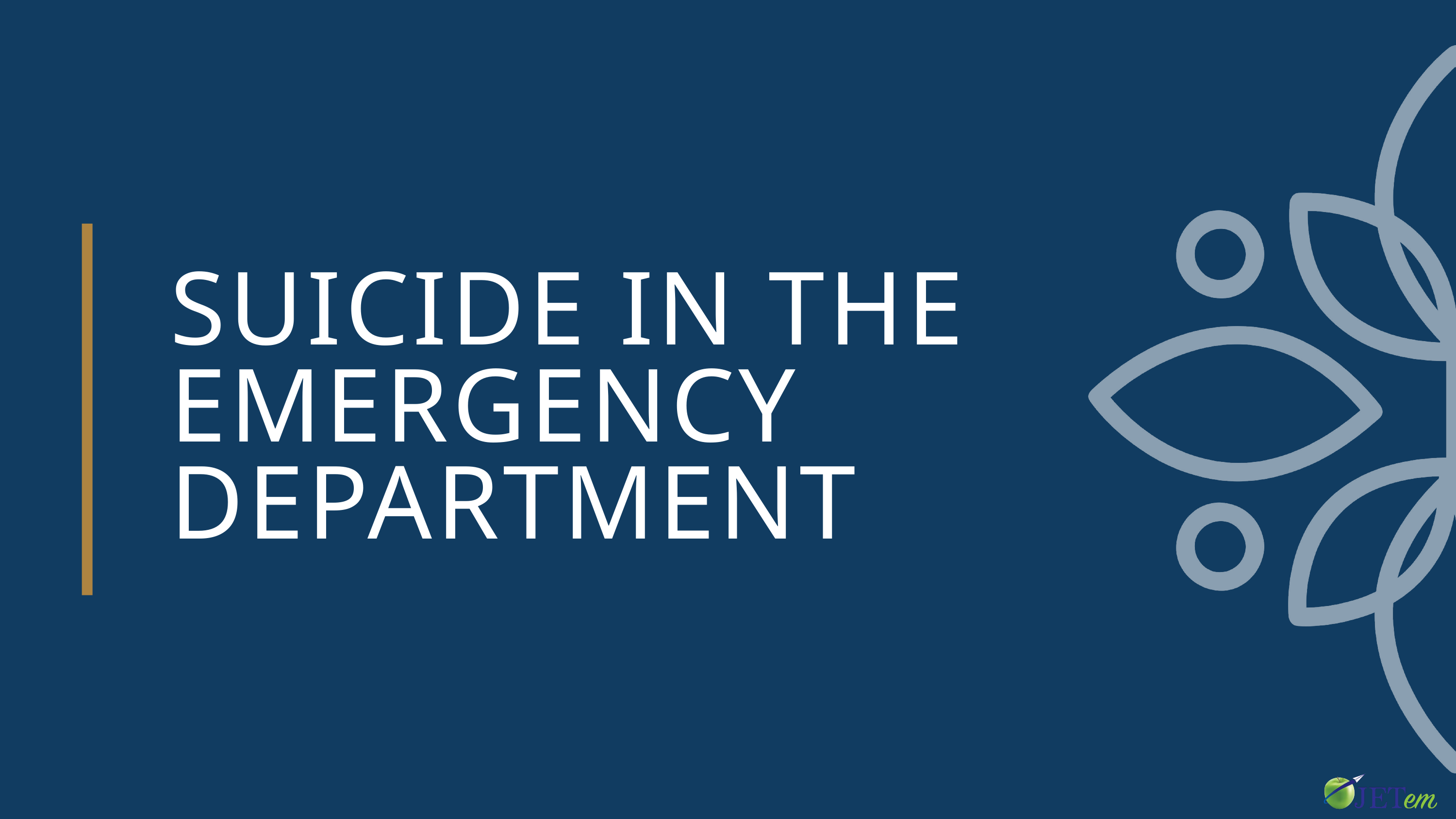

SUICIDE IN THE EMERGENCY DEPARTMENT

## Slide 2
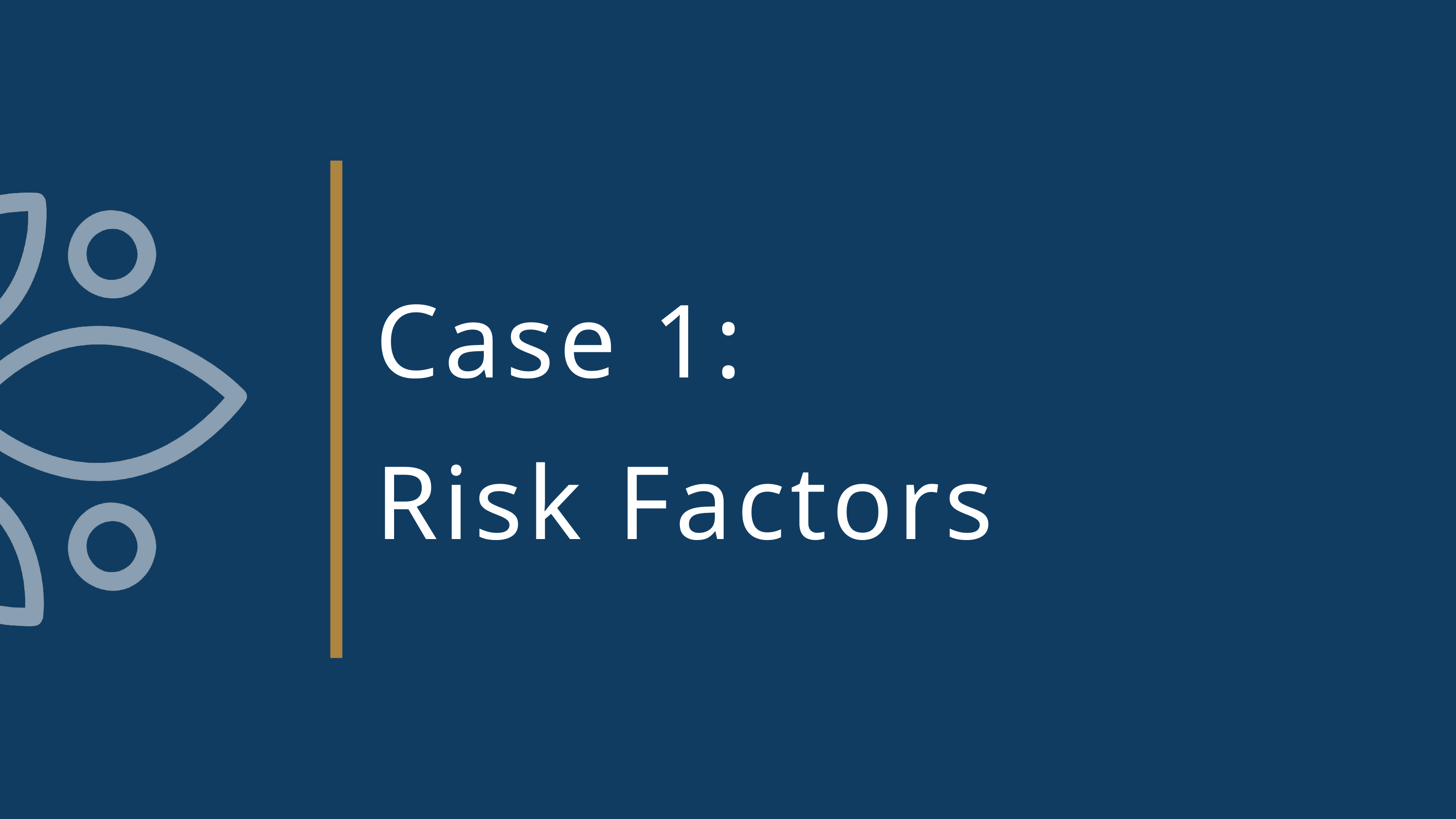

Case 1:
Risk Factors

## Slide 3
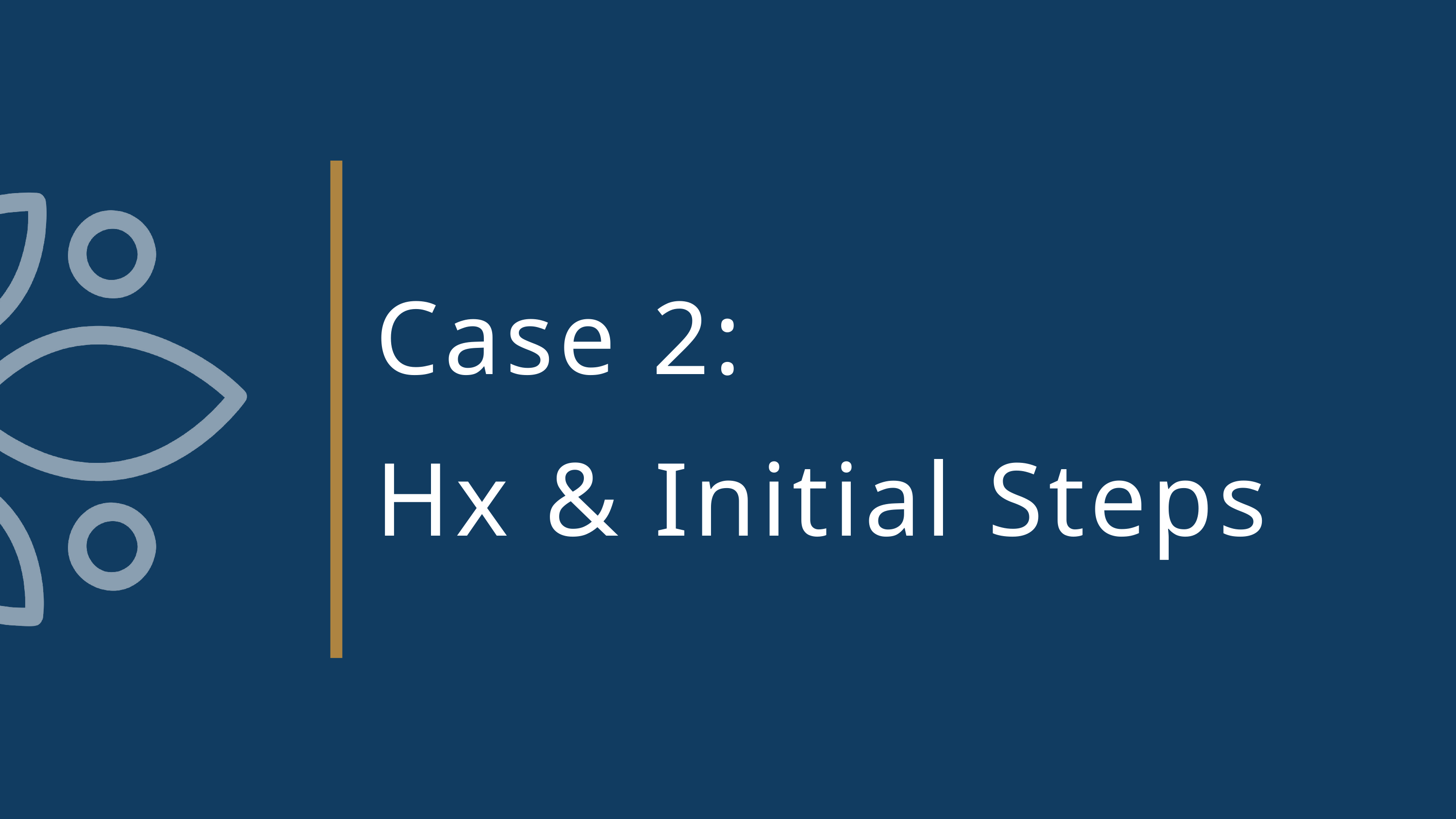

Case 2:
Hx & Initial Steps

## Slide 4
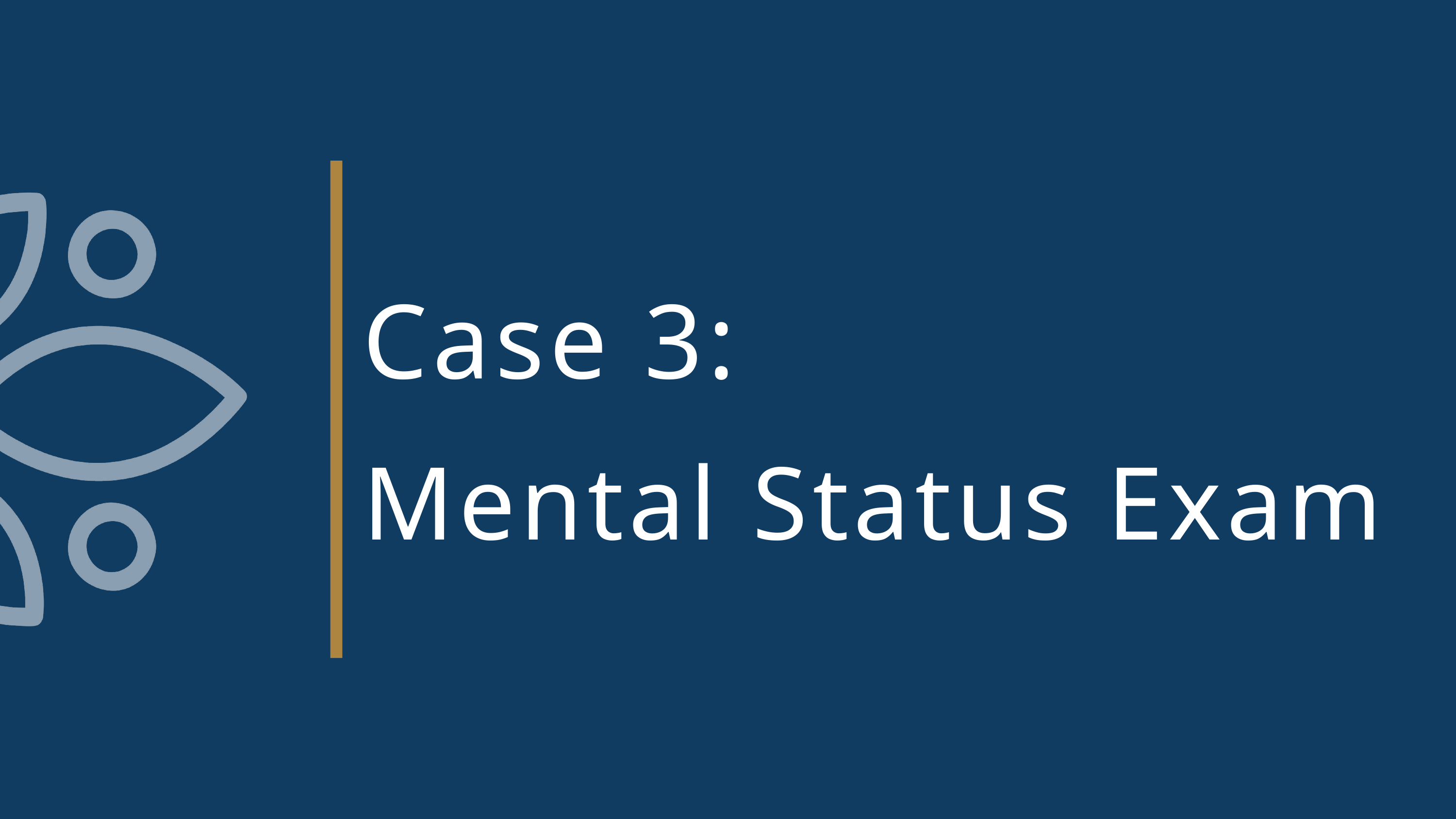

Case 3:
Mental Status Exam

## Slide 5
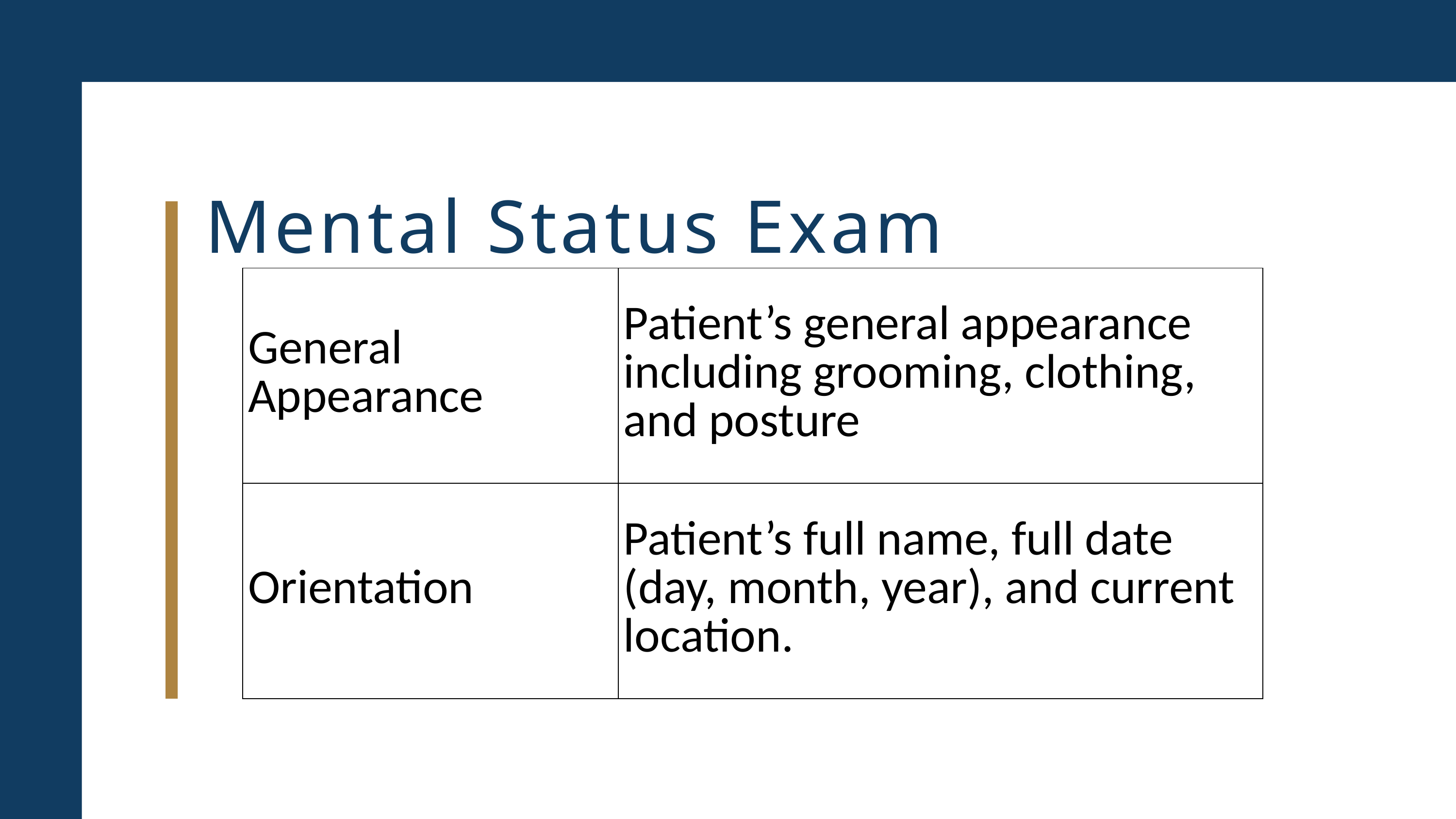

Mental Status Exam
| General Appearance | Patient’s general appearance including grooming, clothing, and posture |
| --- | --- |
| Orientation | Patient’s full name, full date (day, month, year), and current location. |

## Slide 6
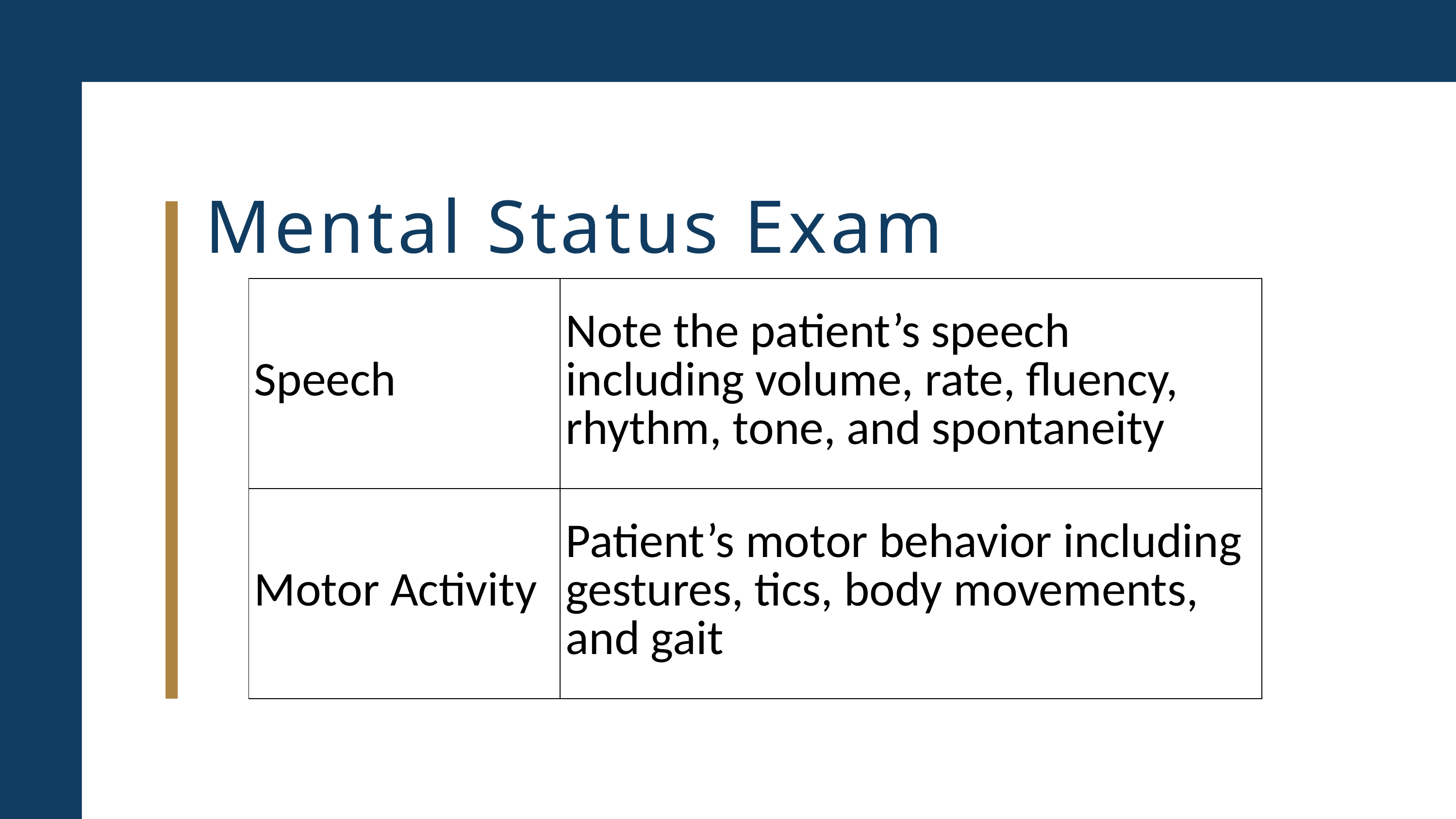

Mental Status Exam
| Speech | Note the patient’s speech including volume, rate, fluency, rhythm, tone, and spontaneity |
| --- | --- |
| Motor Activity | Patient’s motor behavior including gestures, tics, body movements, and gait |

## Slide 7
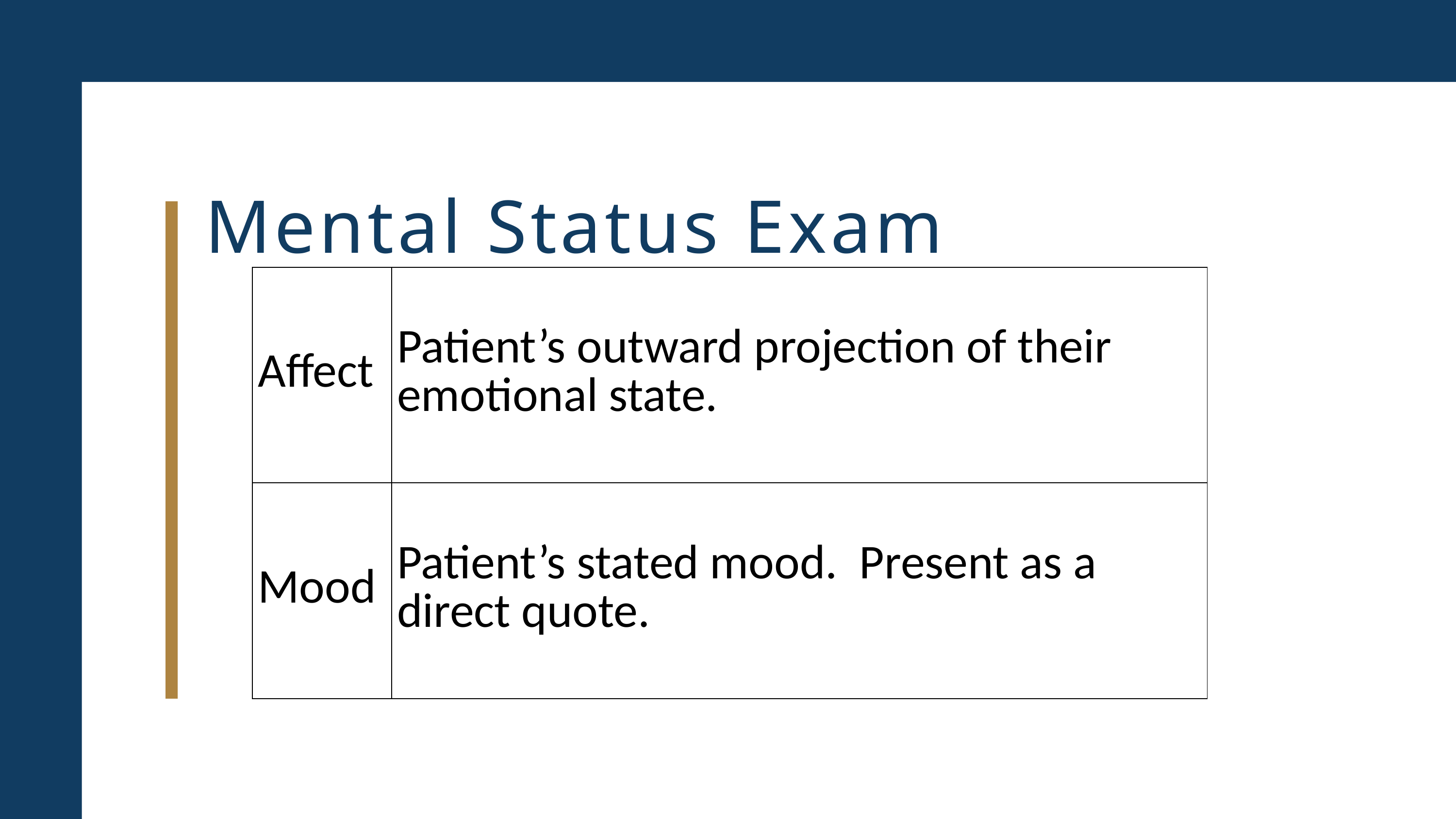

Mental Status Exam
| Affect | Patient’s outward projection of their emotional state. |
| --- | --- |
| Mood | Patient’s stated mood. Present as a direct quote. |

## Slide 8
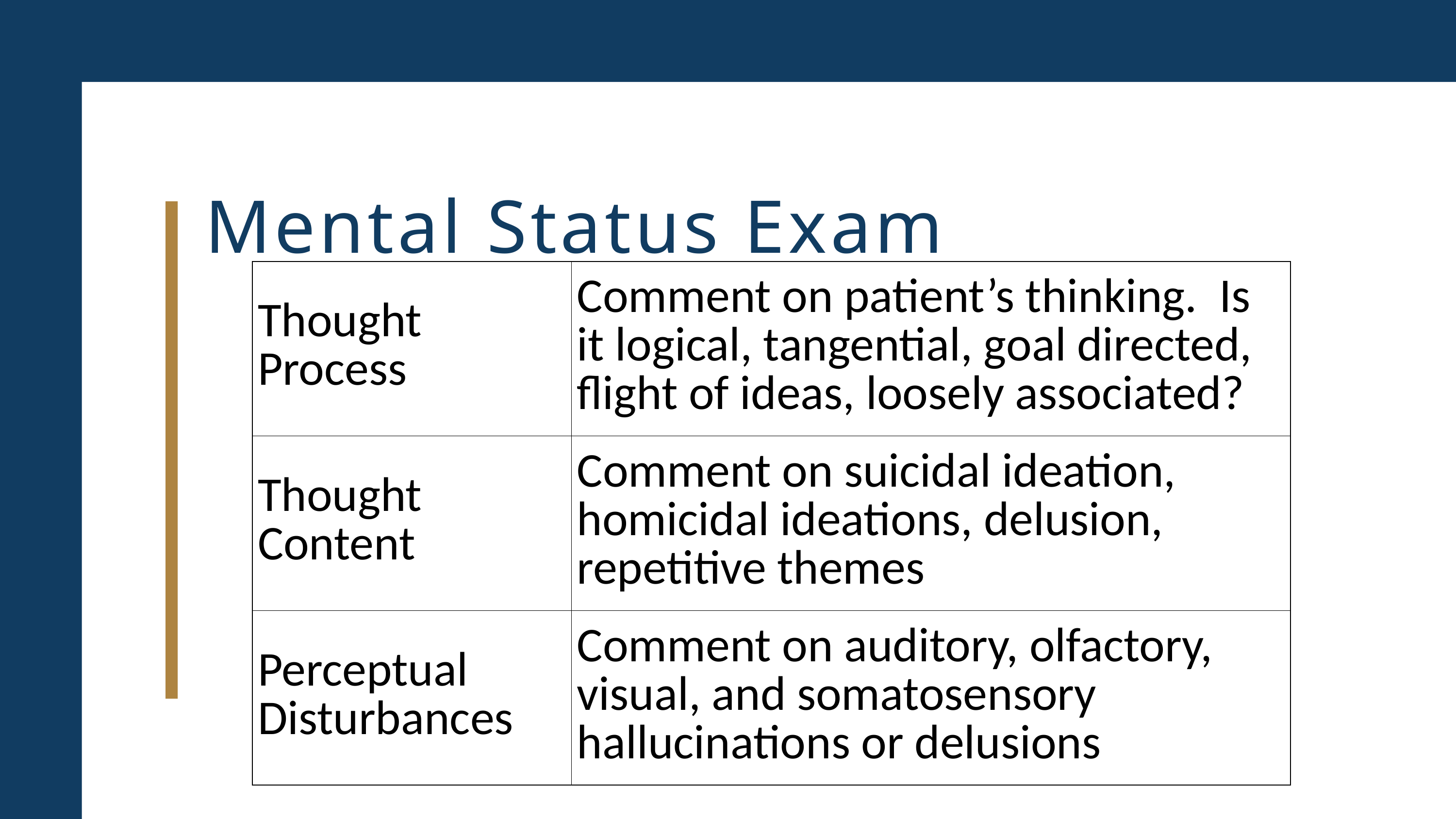

Mental Status Exam
| Thought Process | Comment on patient’s thinking. Is it logical, tangential, goal directed, flight of ideas, loosely associated? |
| --- | --- |
| Thought Content | Comment on suicidal ideation, homicidal ideations, delusion, repetitive themes |
| Perceptual Disturbances | Comment on auditory, olfactory, visual, and somatosensory hallucinations or delusions |

## Slide 9
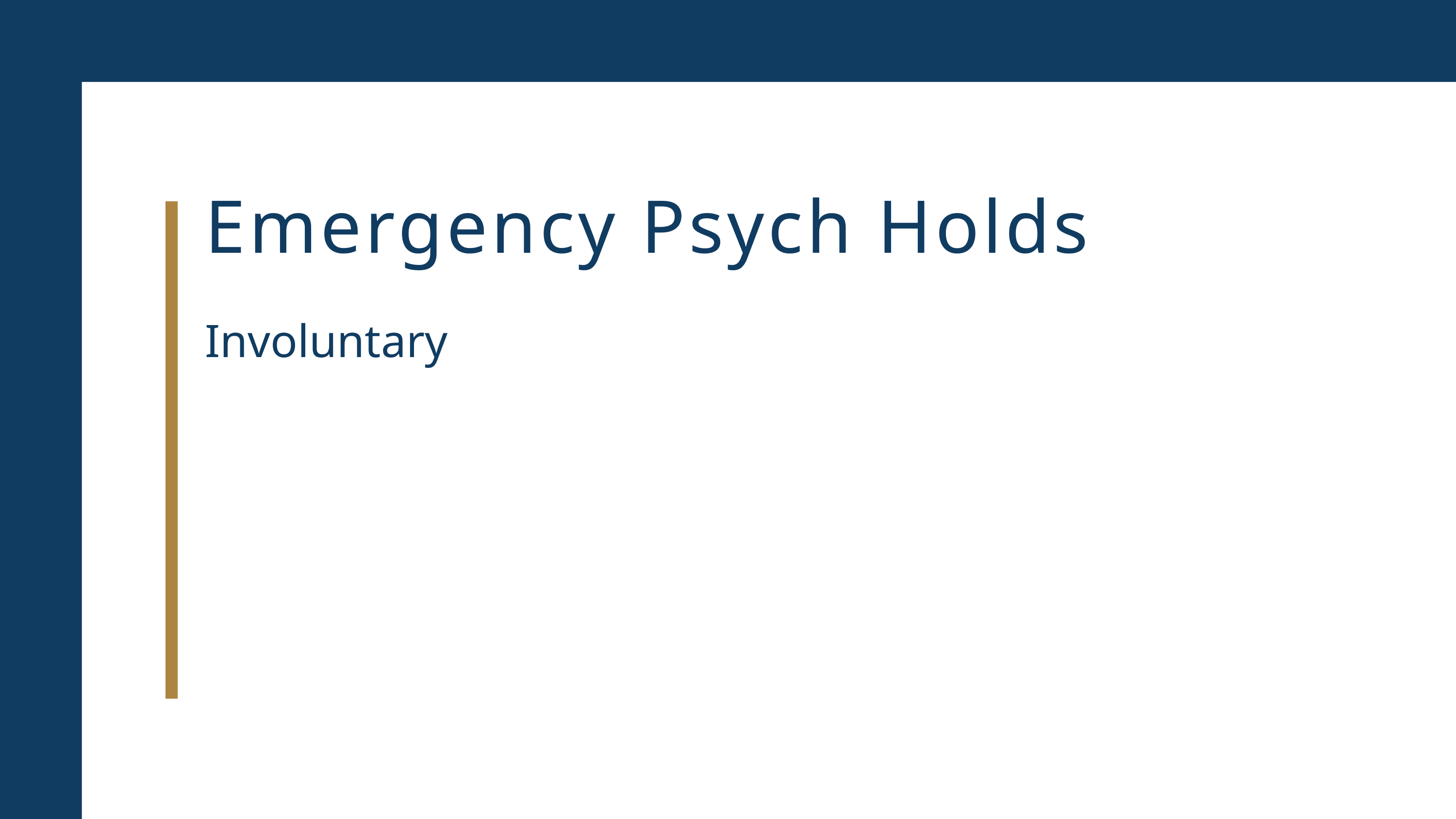

Emergency Psych Holds
Involuntary

## Slide 10
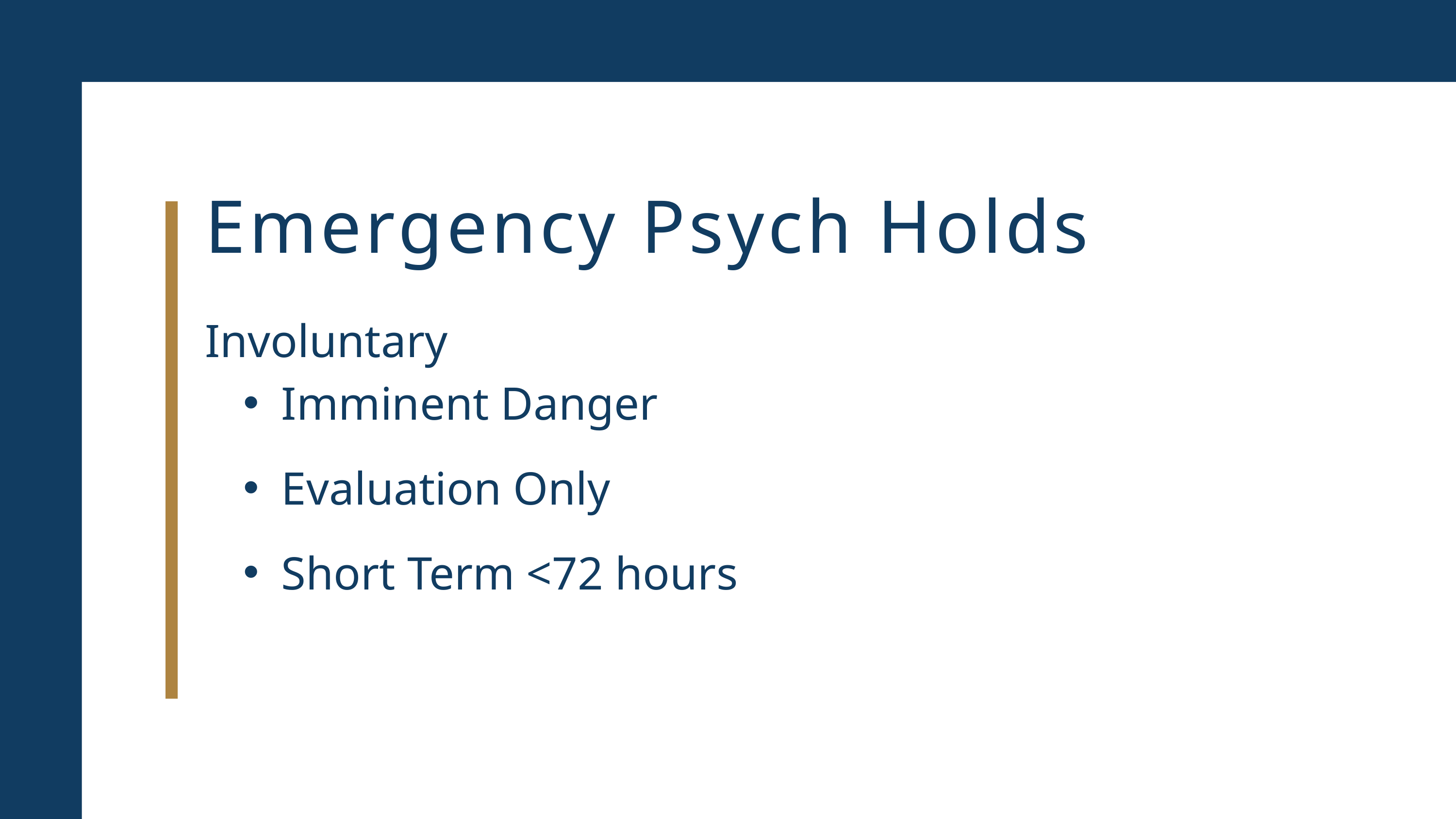

Emergency Psych Holds
Involuntary
Imminent Danger
Evaluation Only
Short Term <72 hours

## Slide 11
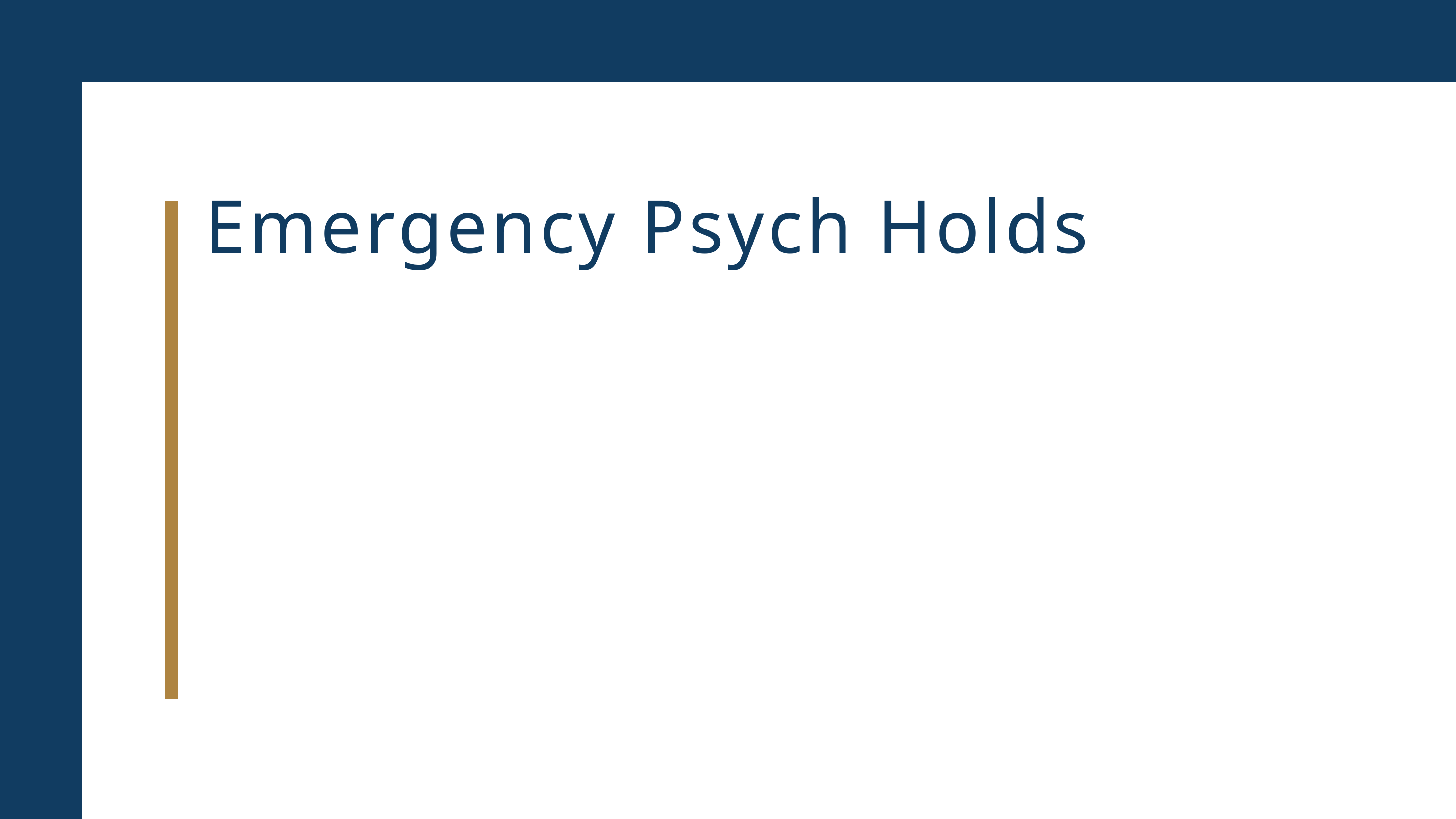

Emergency Psych Holds

## Slide 12
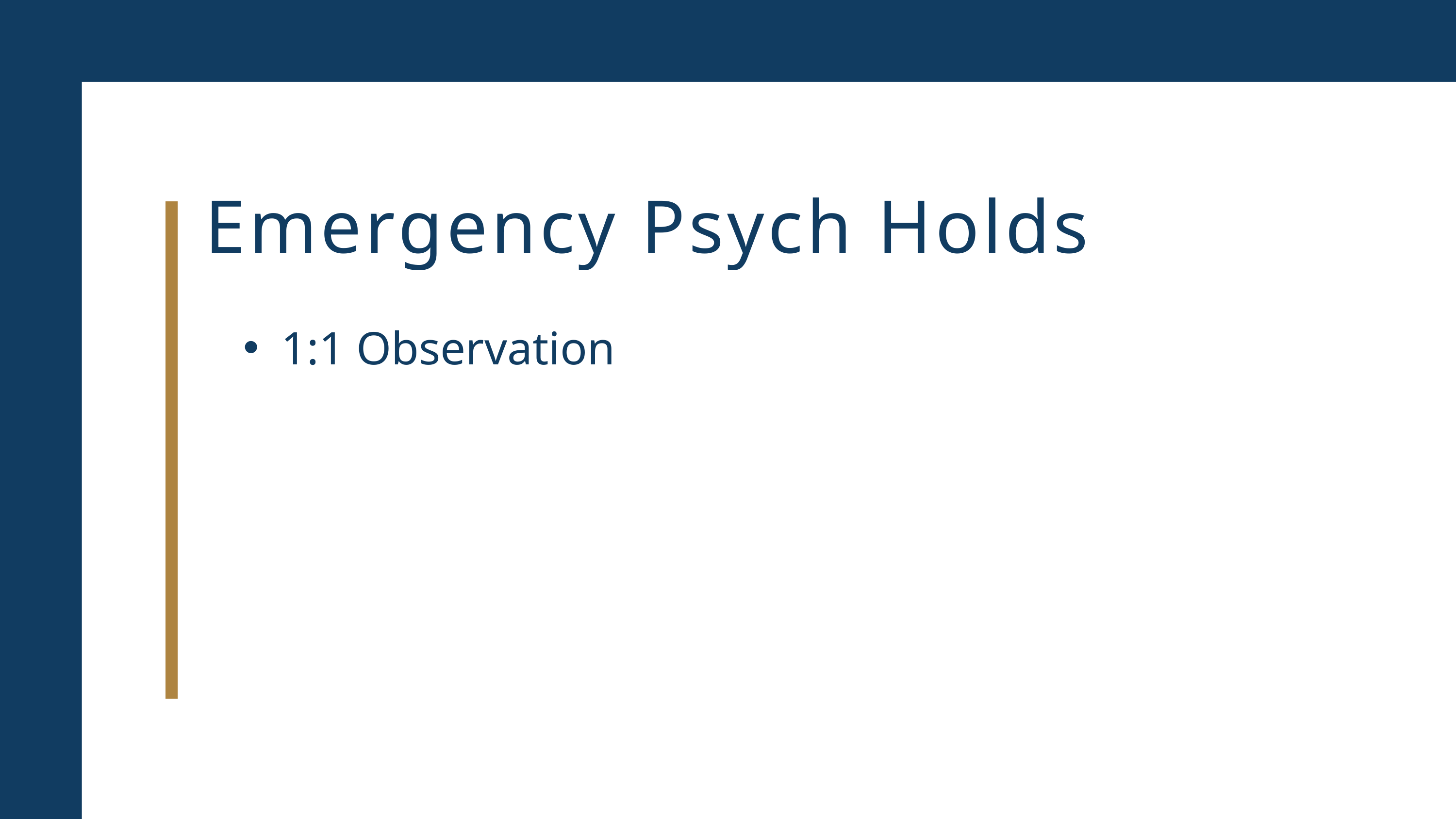

Emergency Psych Holds
1:1 Observation

## Slide 13
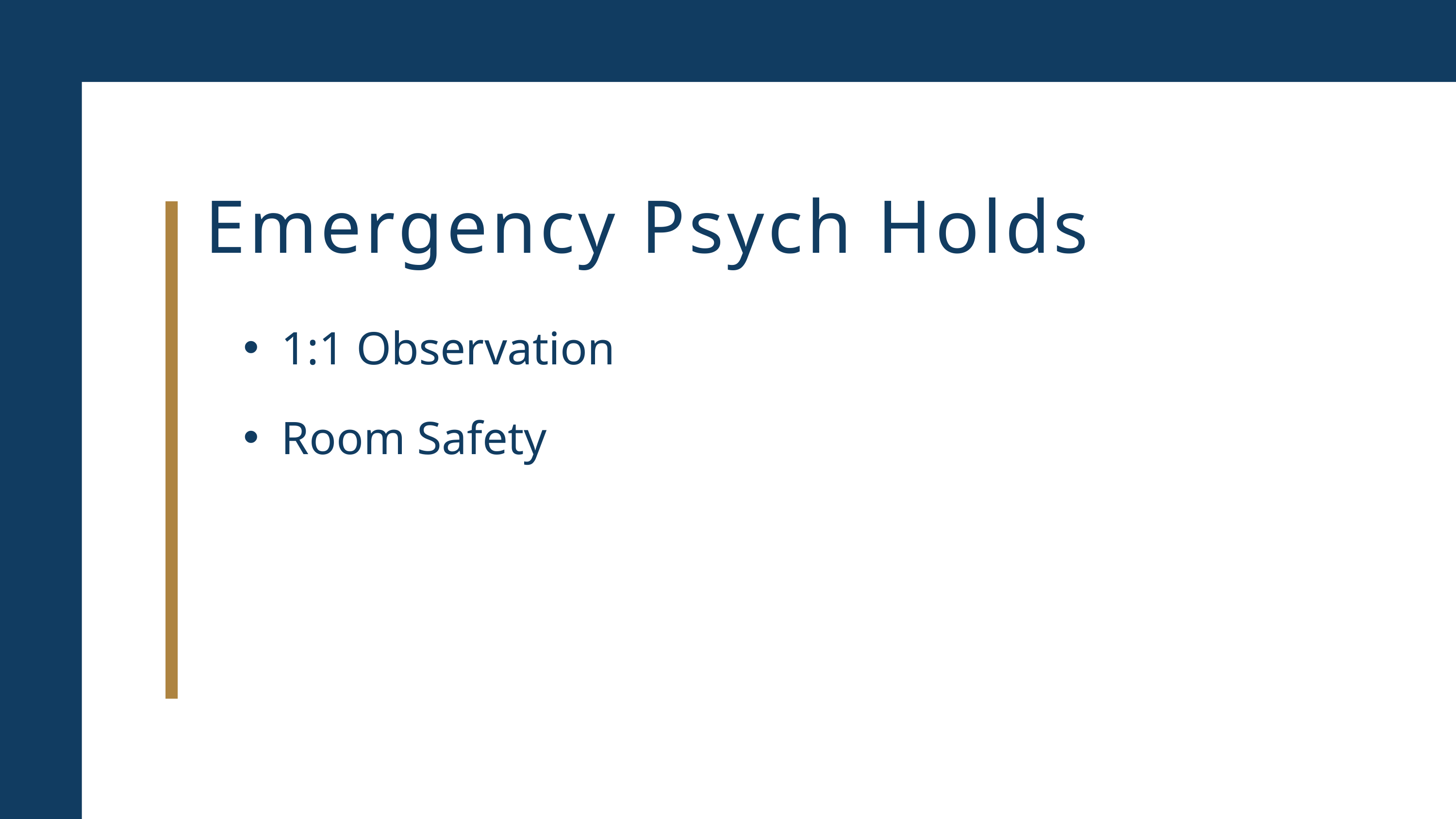

Emergency Psych Holds
1:1 Observation
Room Safety

## Slide 14
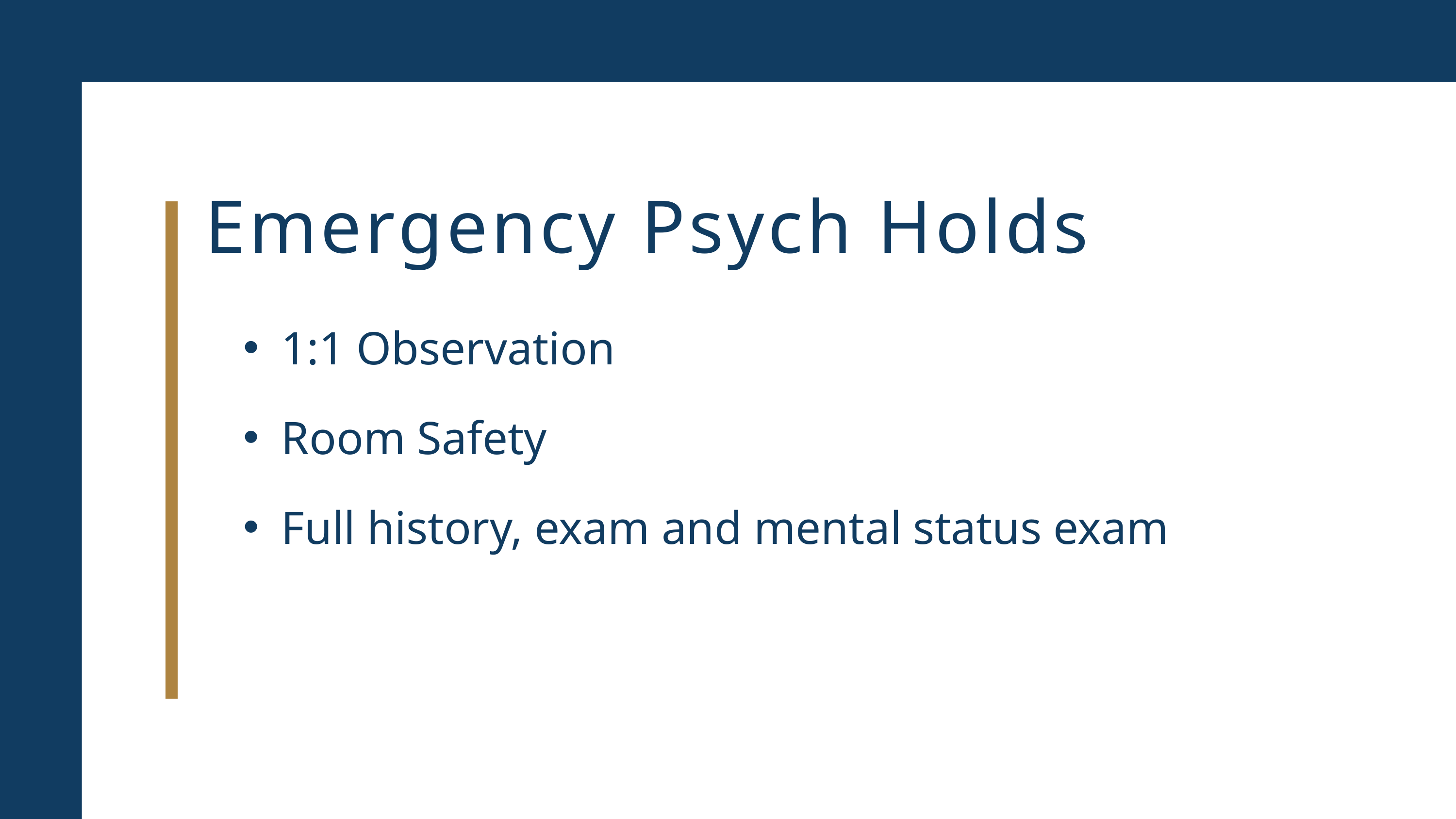

Emergency Psych Holds
1:1 Observation
Room Safety
Full history, exam and mental status exam

## Slide 15
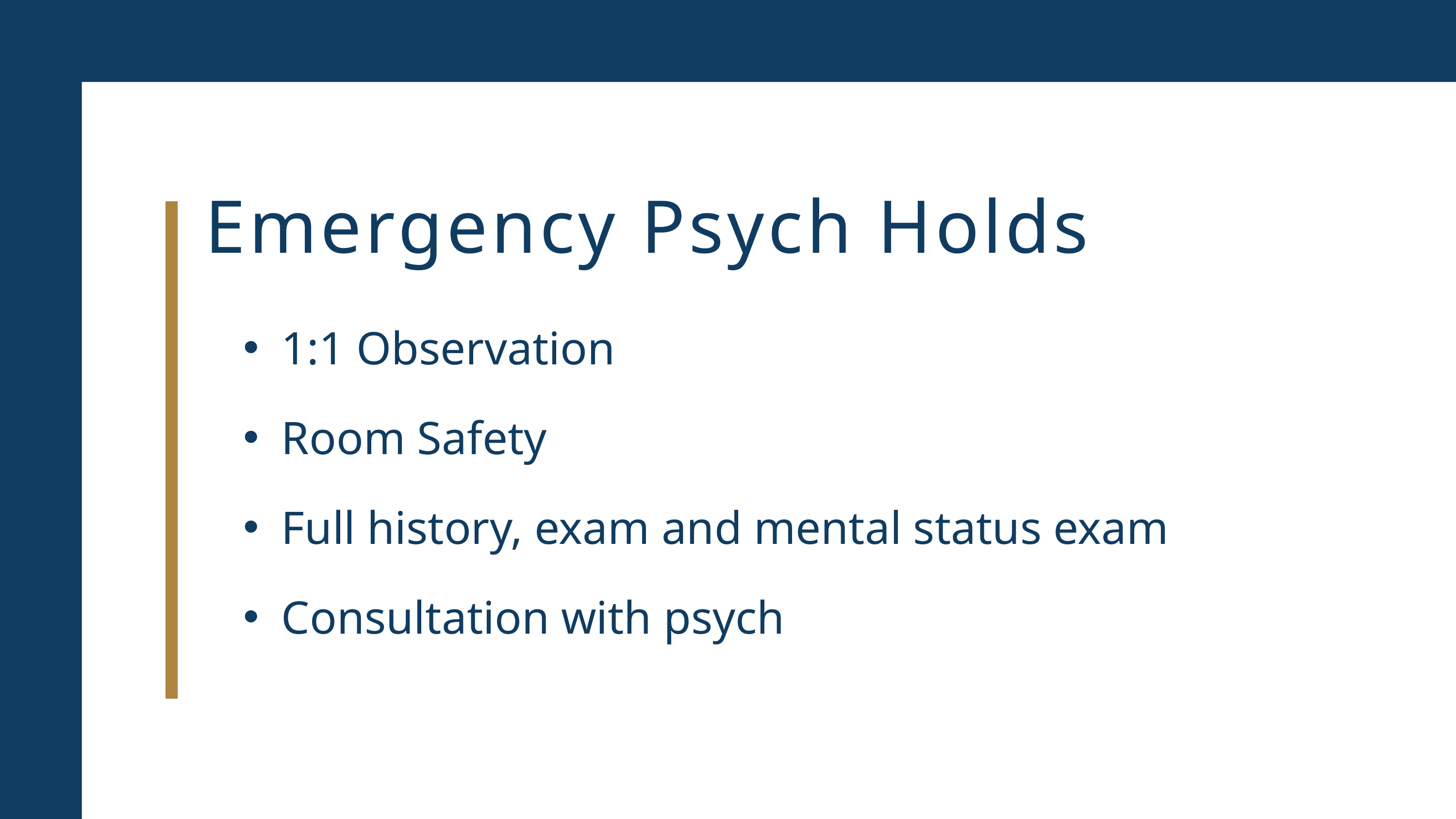

Emergency Psych Holds
1:1 Observation
Room Safety
Full history, exam and mental status exam
Consultation with psych

## Slide 16
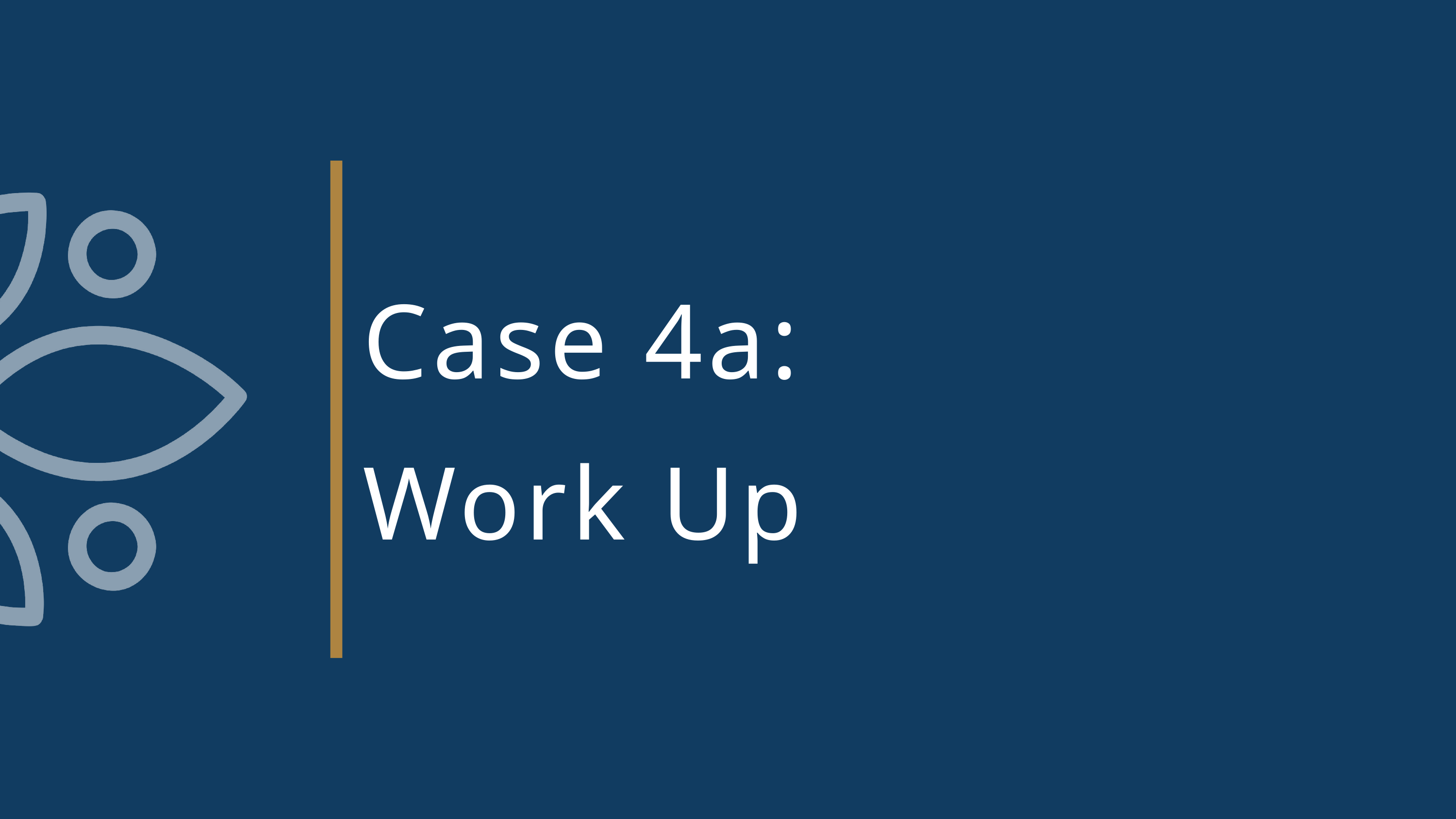

Case 4a:
Work Up

## Slide 17
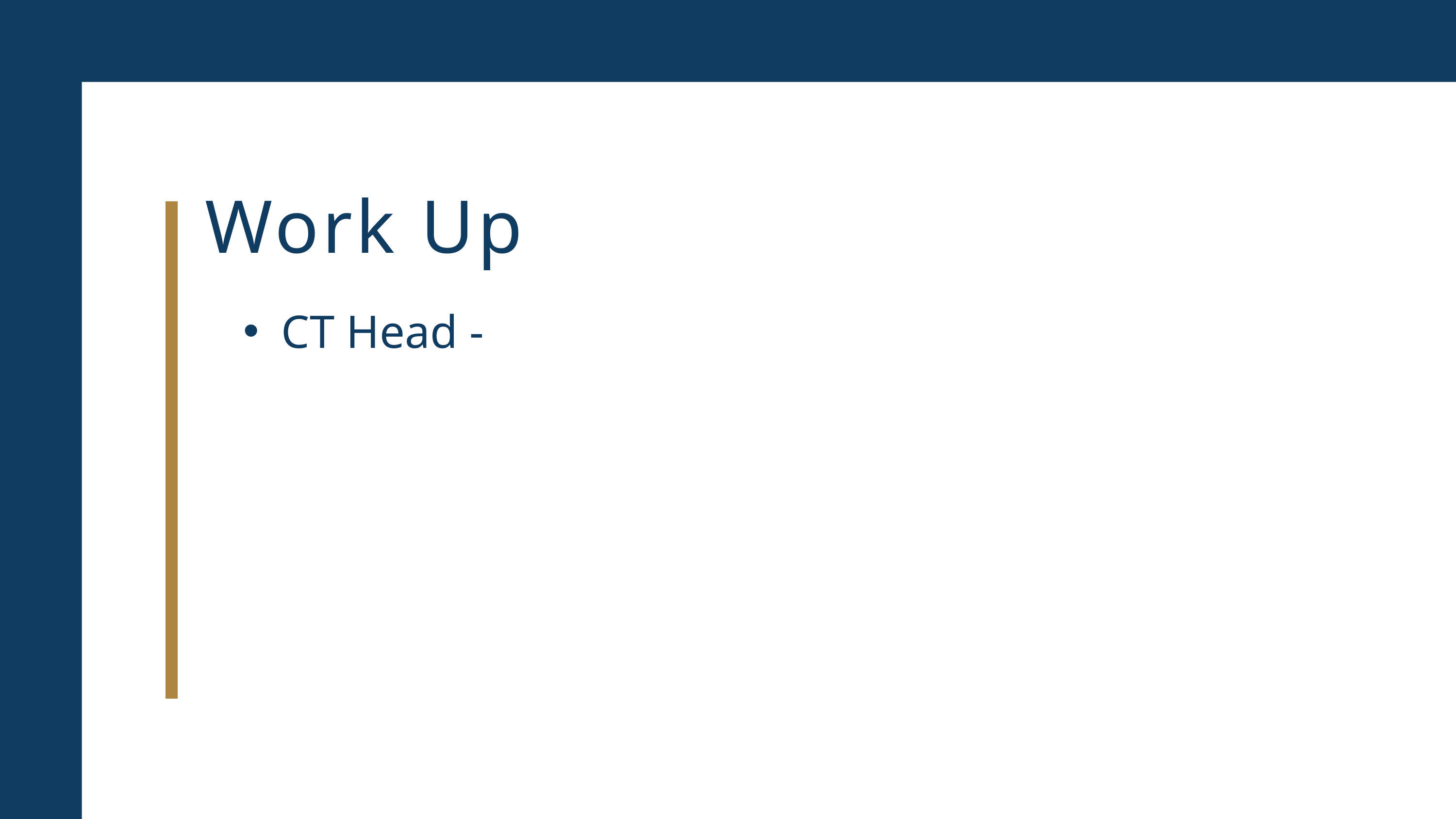

Work Up
CT Head -

## Slide 18
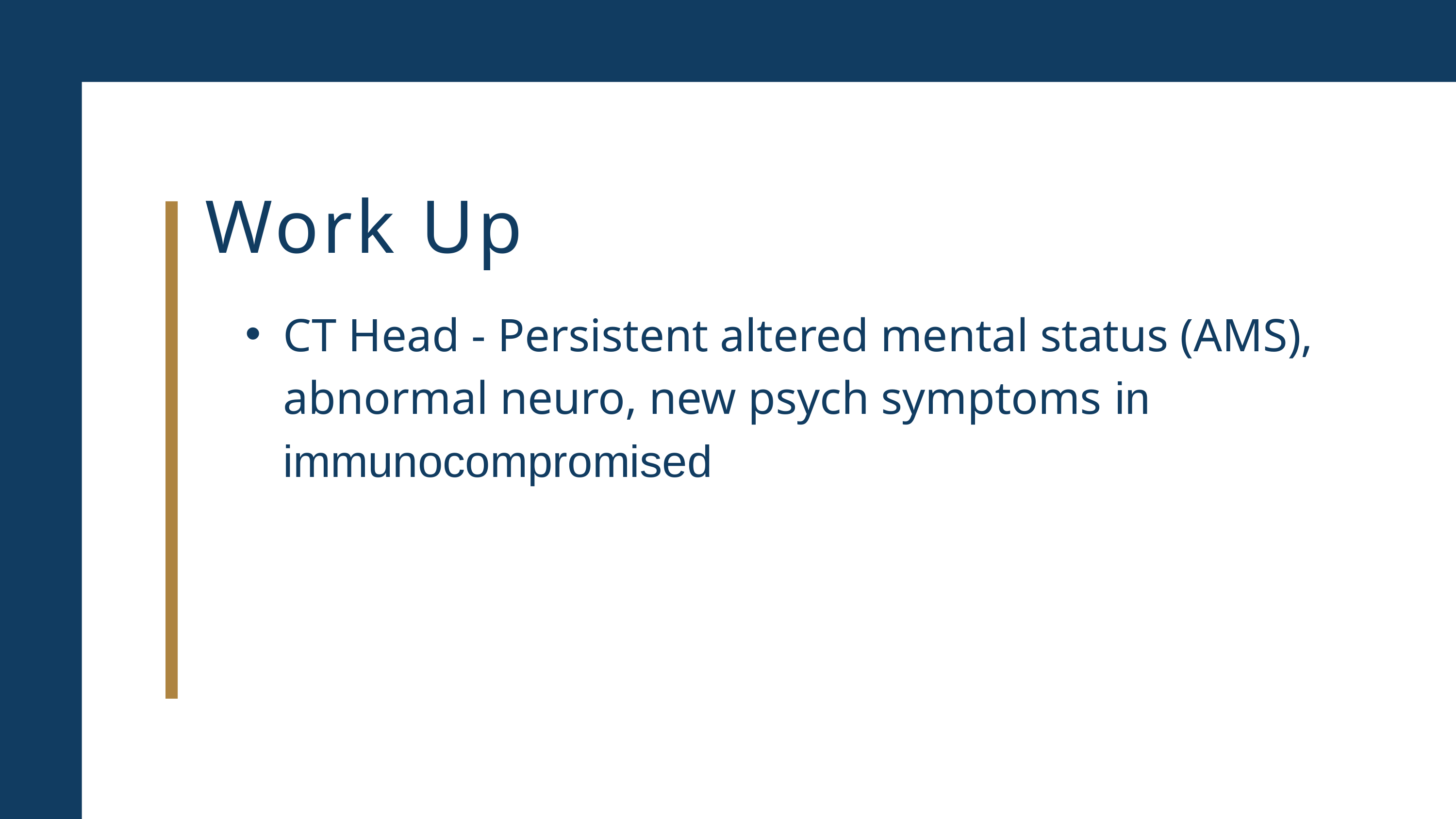

Work Up
CT Head - Persistent altered mental status (AMS), abnormal neuro, new psych symptoms in immunocompromised

## Slide 19
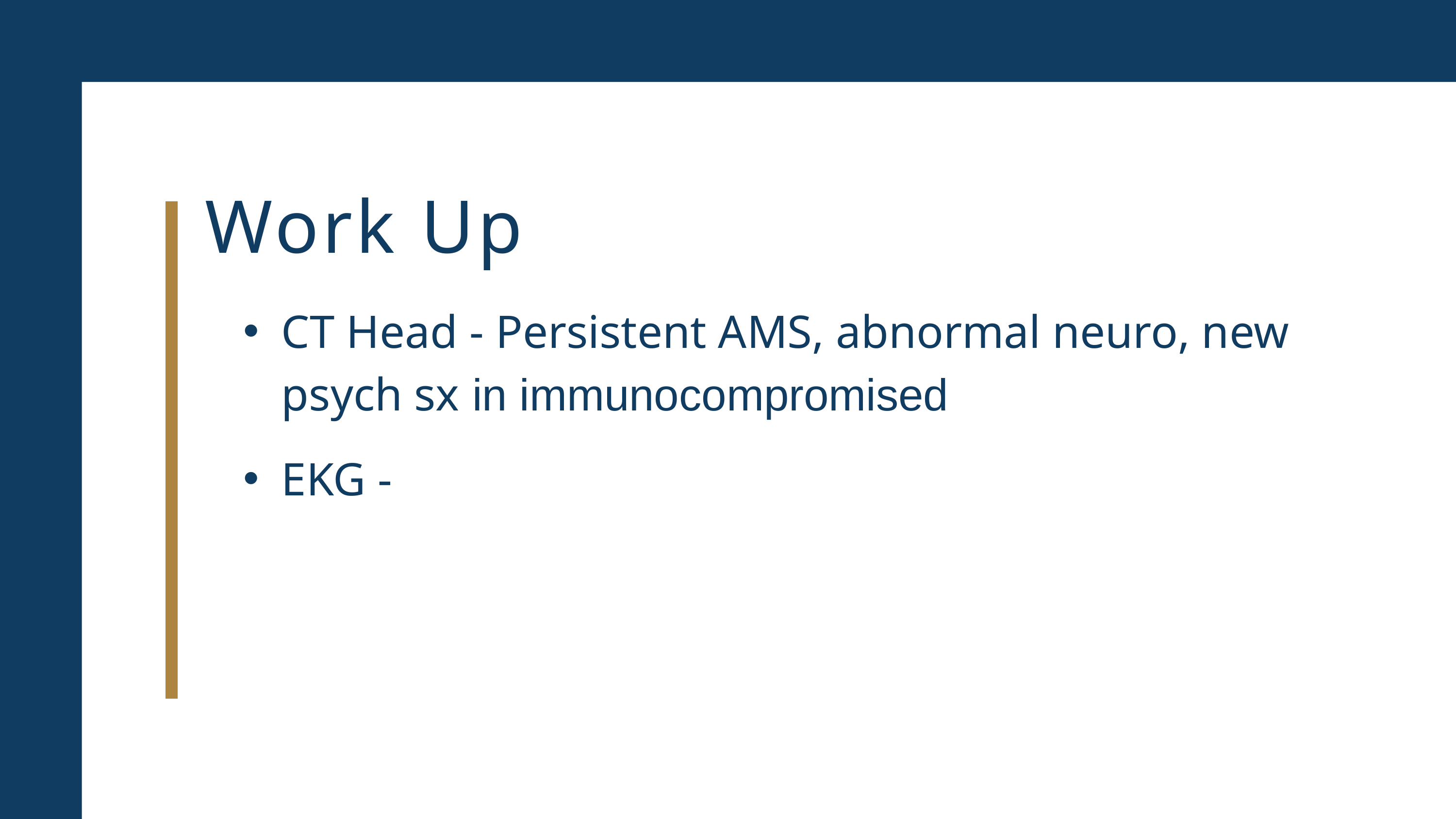

Work Up
CT Head - Persistent AMS, abnormal neuro, new psych sx in immunocompromised
EKG -

## Slide 20
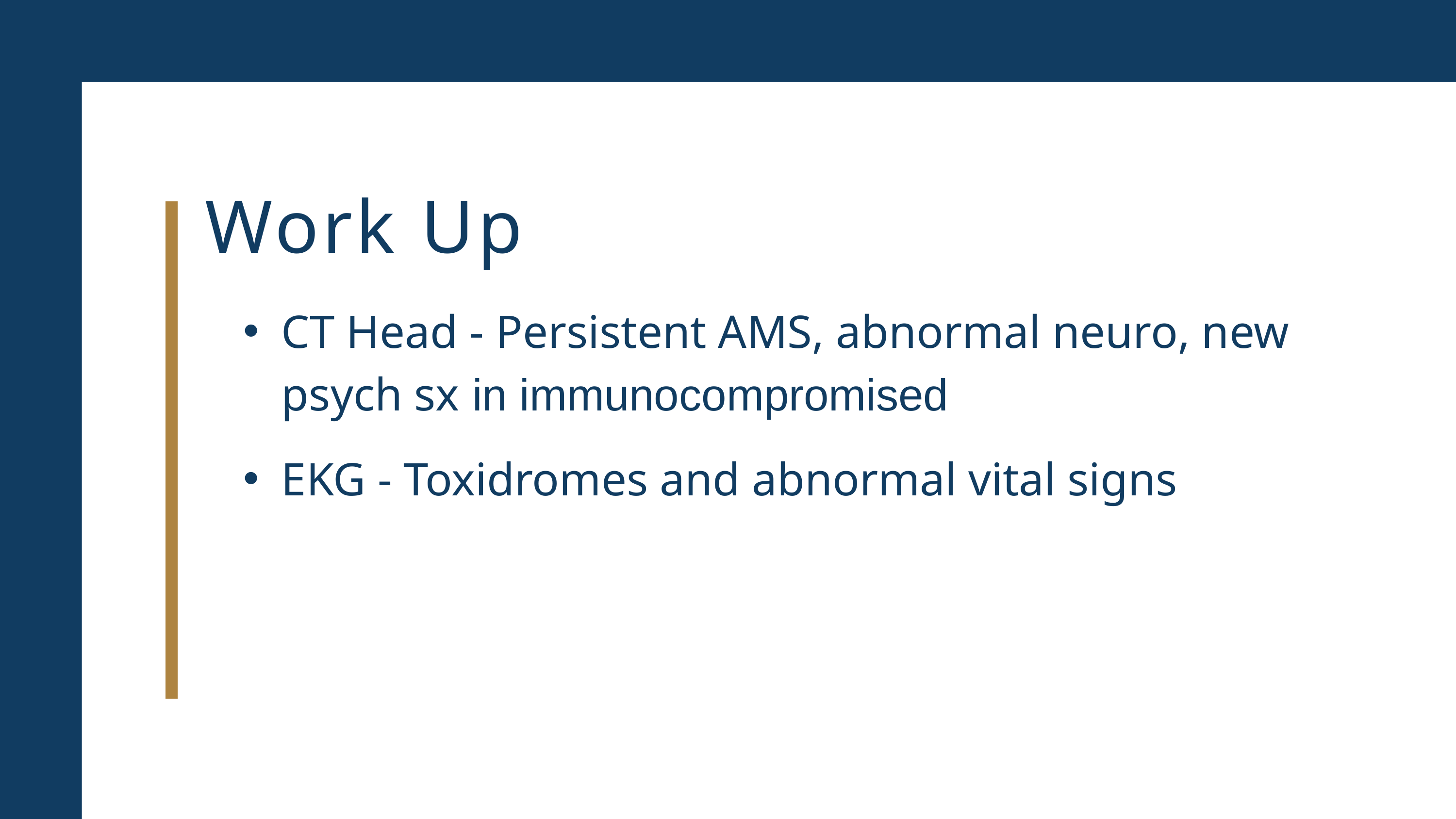

Work Up
CT Head - Persistent AMS, abnormal neuro, new psych sx in immunocompromised
EKG - Toxidromes and abnormal vital signs

## Slide 21
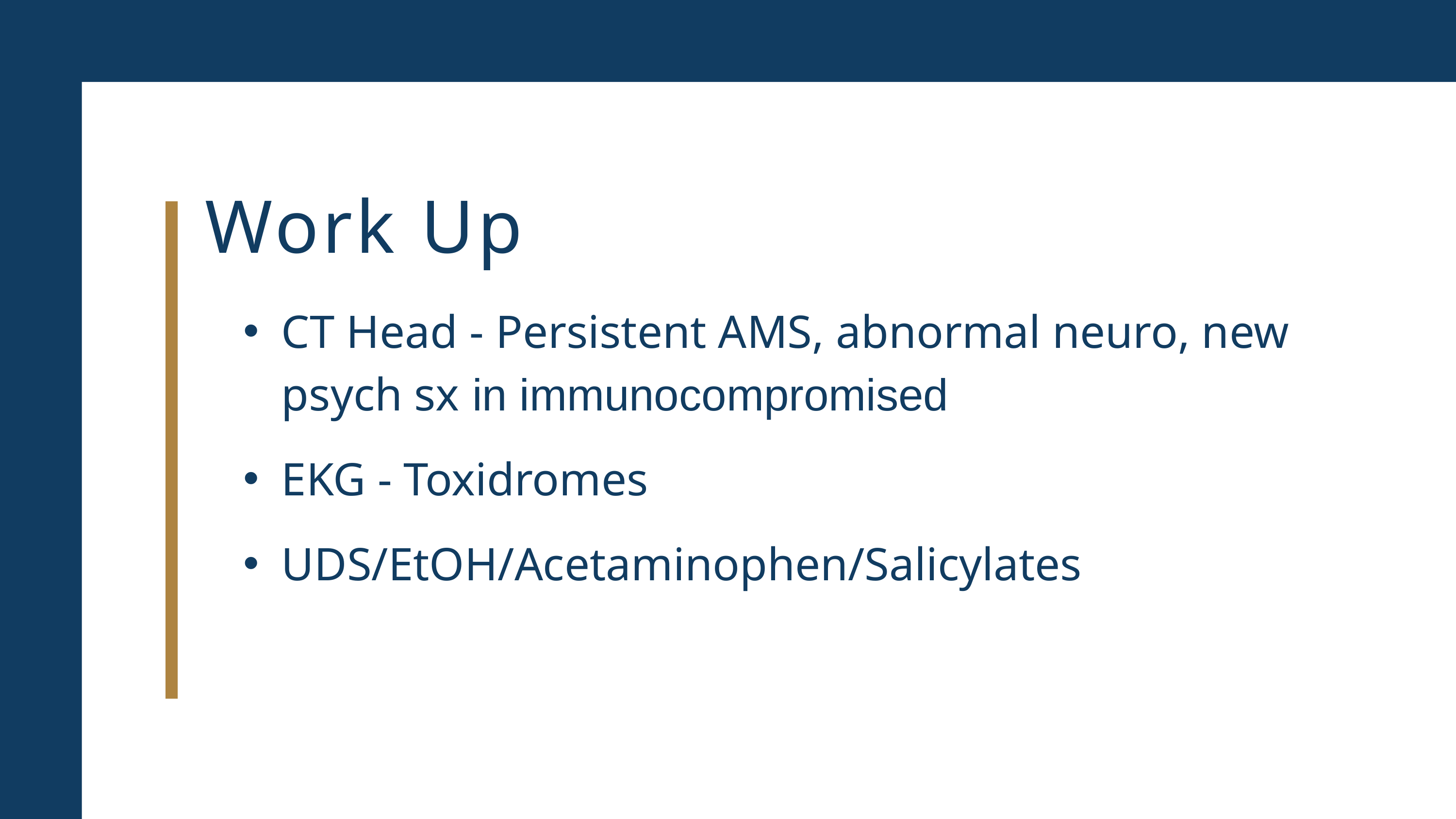

Work Up
CT Head - Persistent AMS, abnormal neuro, new psych sx in immunocompromised
EKG - Toxidromes
UDS/EtOH/Acetaminophen/Salicylates

## Slide 22
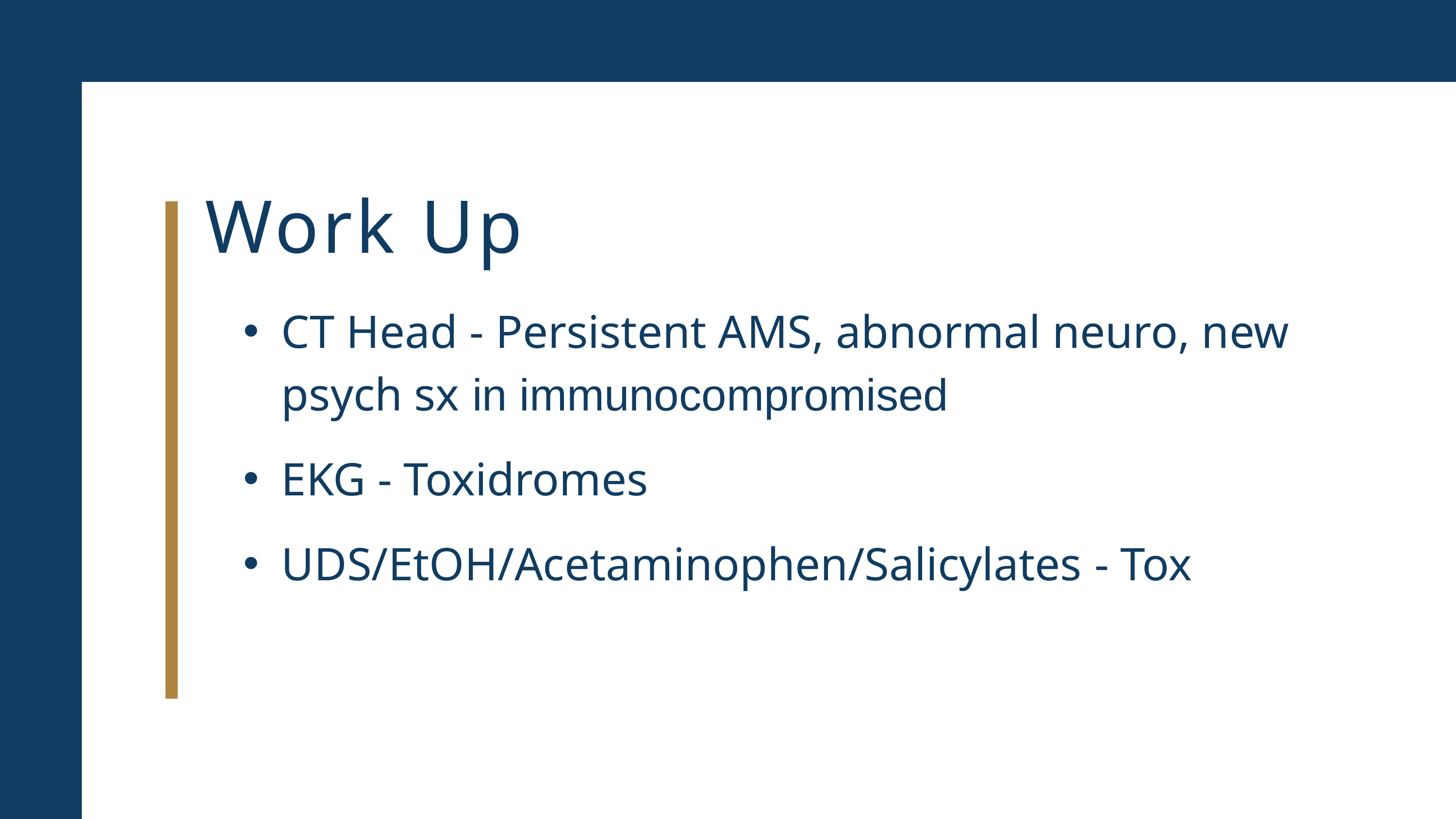

Work Up
CT Head - Persistent AMS, abnormal neuro, new psych sx in immunocompromised
EKG - Toxidromes
UDS/EtOH/Acetaminophen/Salicylates - Tox

## Slide 23
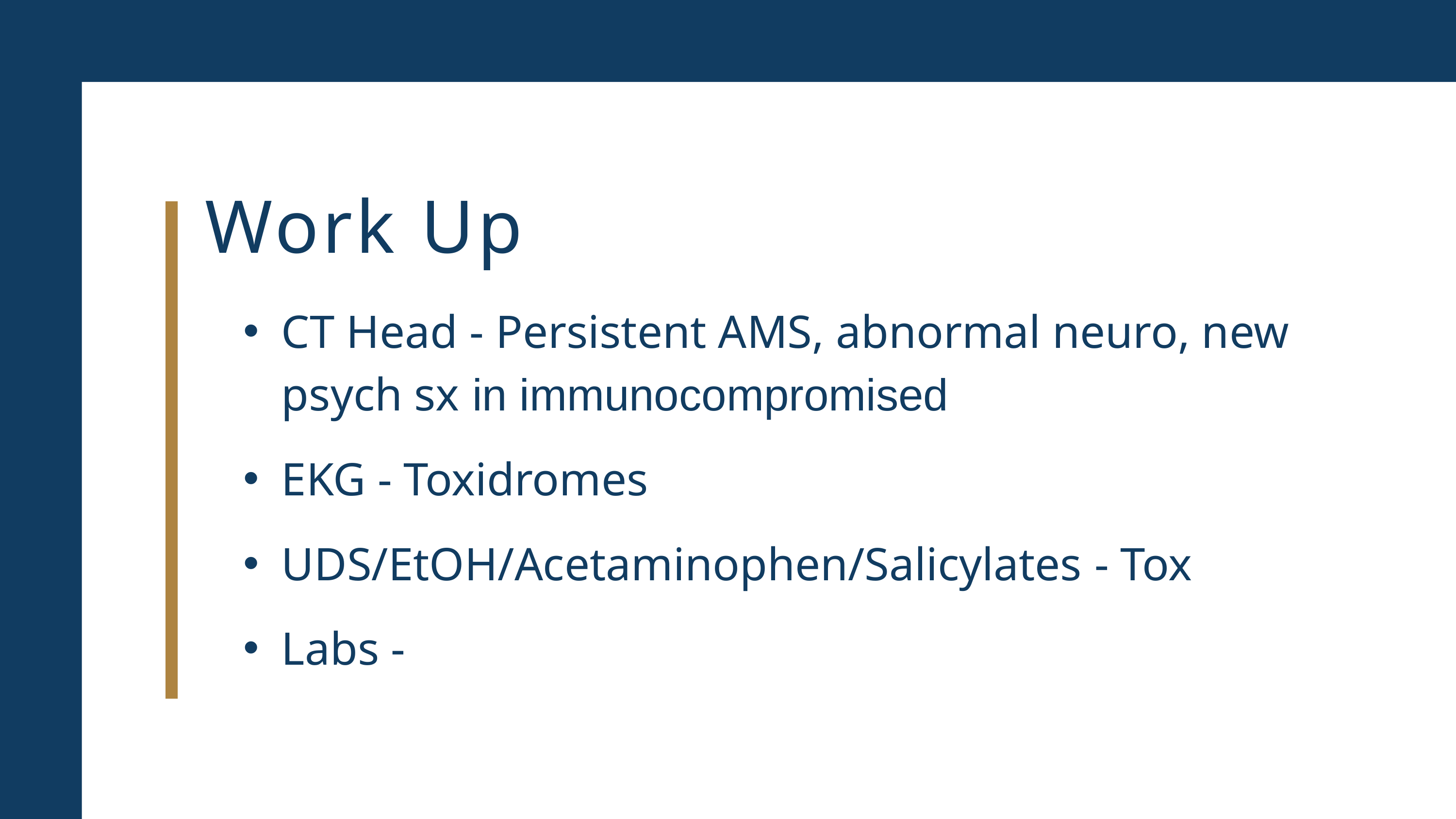

Work Up
CT Head - Persistent AMS, abnormal neuro, new psych sx in immunocompromised
EKG - Toxidromes
UDS/EtOH/Acetaminophen/Salicylates - Tox
Labs -

## Slide 24
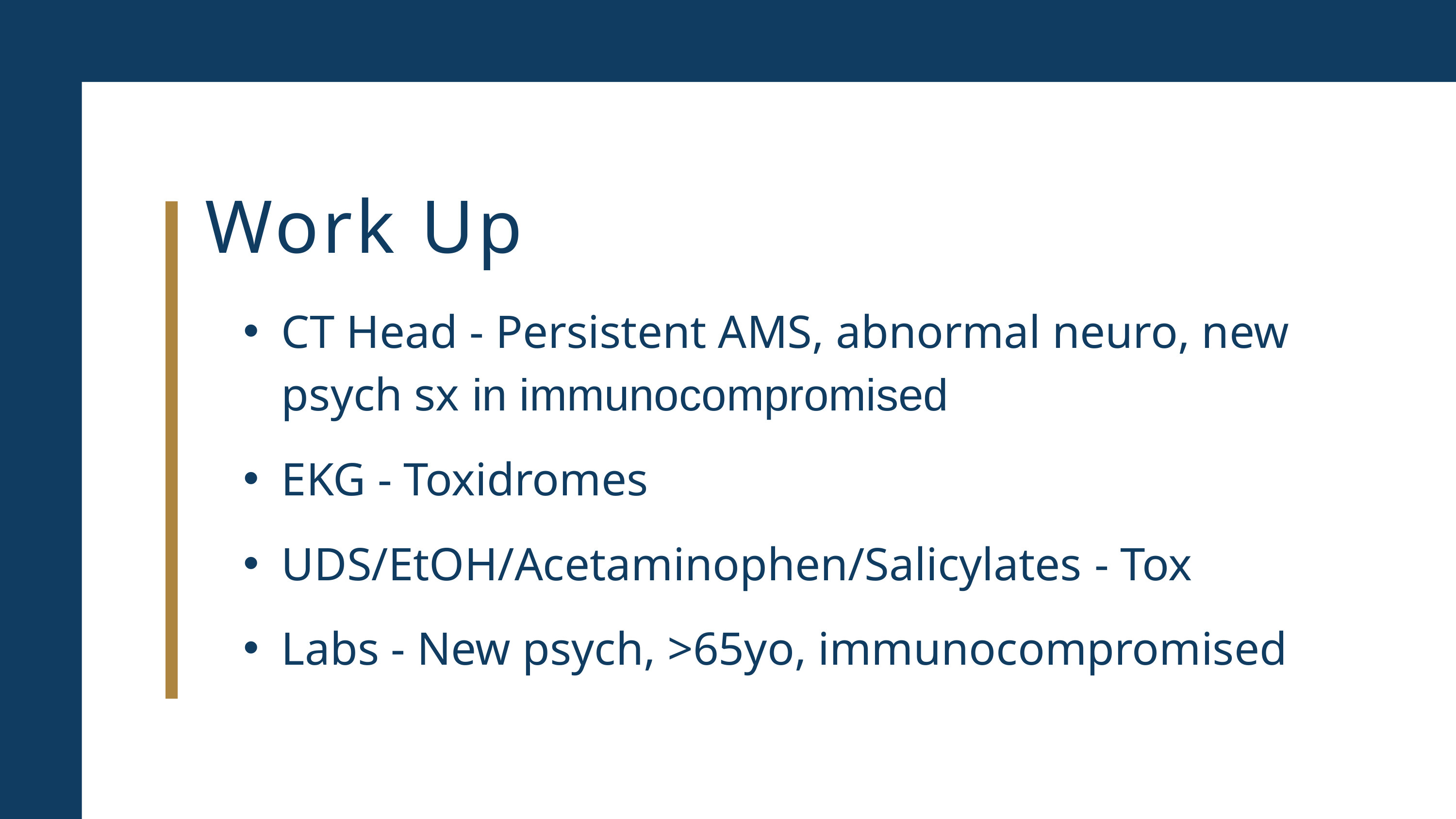

Work Up
CT Head - Persistent AMS, abnormal neuro, new psych sx in immunocompromised
EKG - Toxidromes
UDS/EtOH/Acetaminophen/Salicylates - Tox
Labs - New psych, >65yo, immunocompromised

## Slide 25
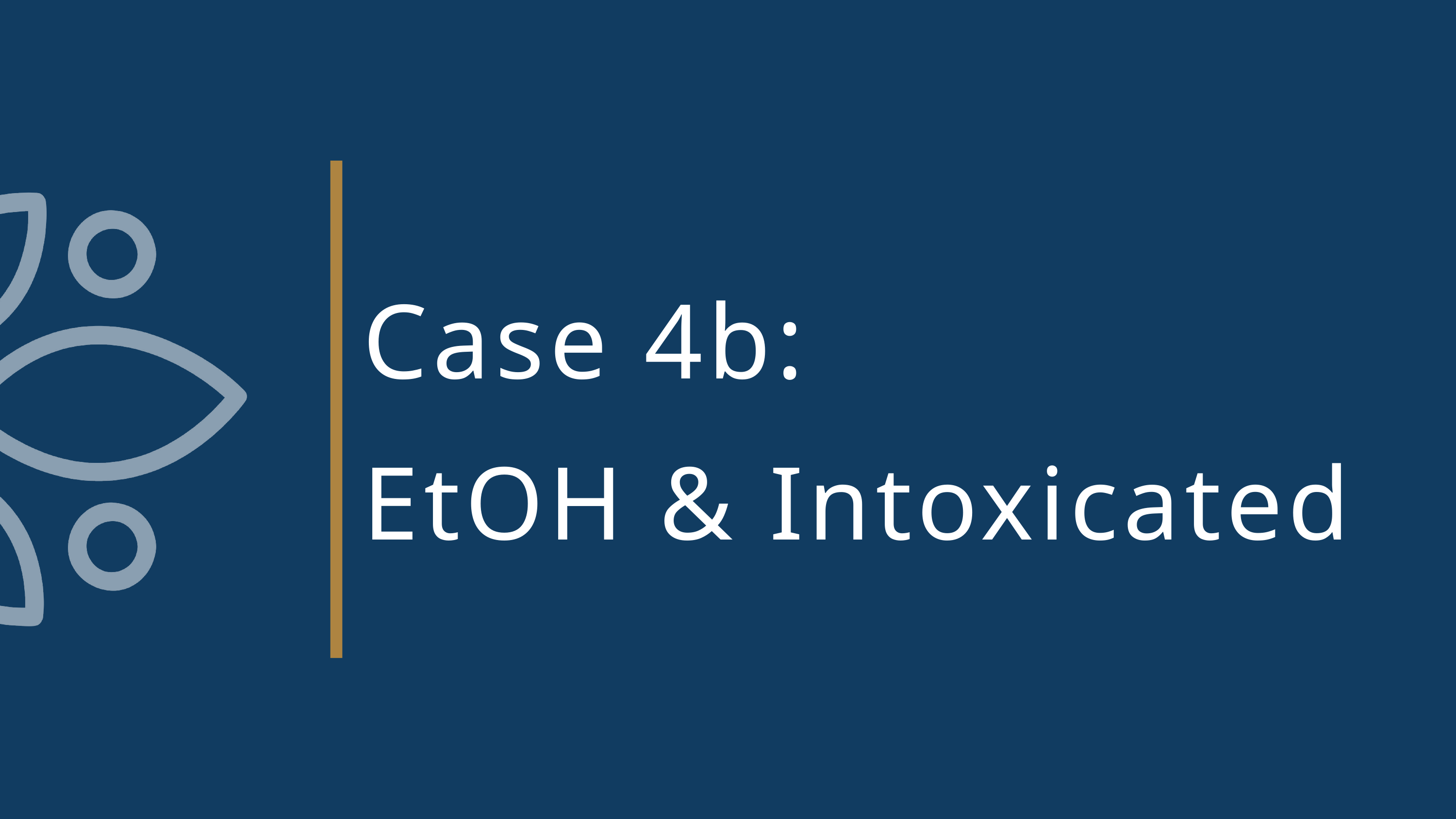

Case 4b:
EtOH & Intoxicated

## Slide 26
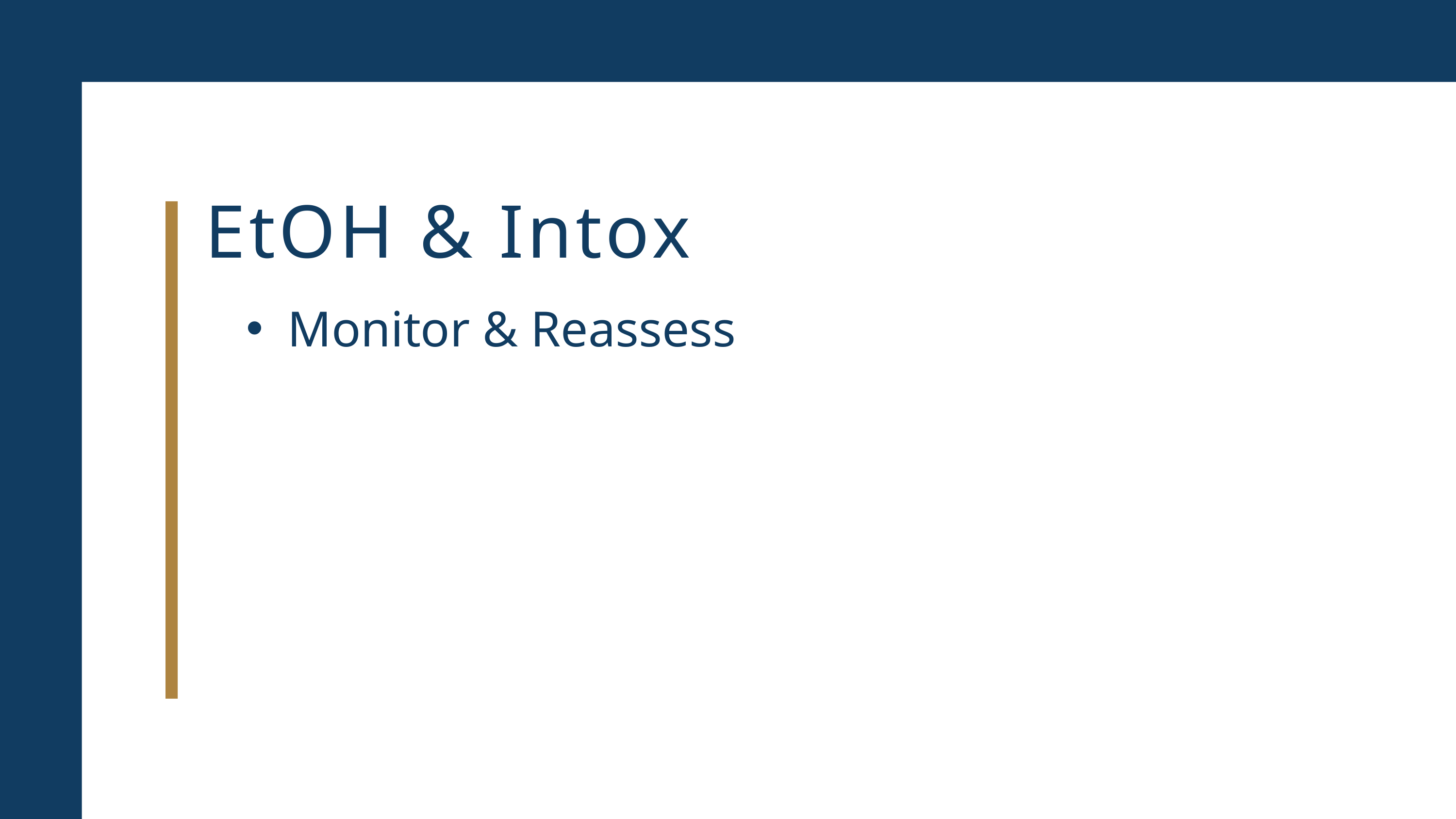

EtOH & Intox
Monitor & Reassess

## Slide 27
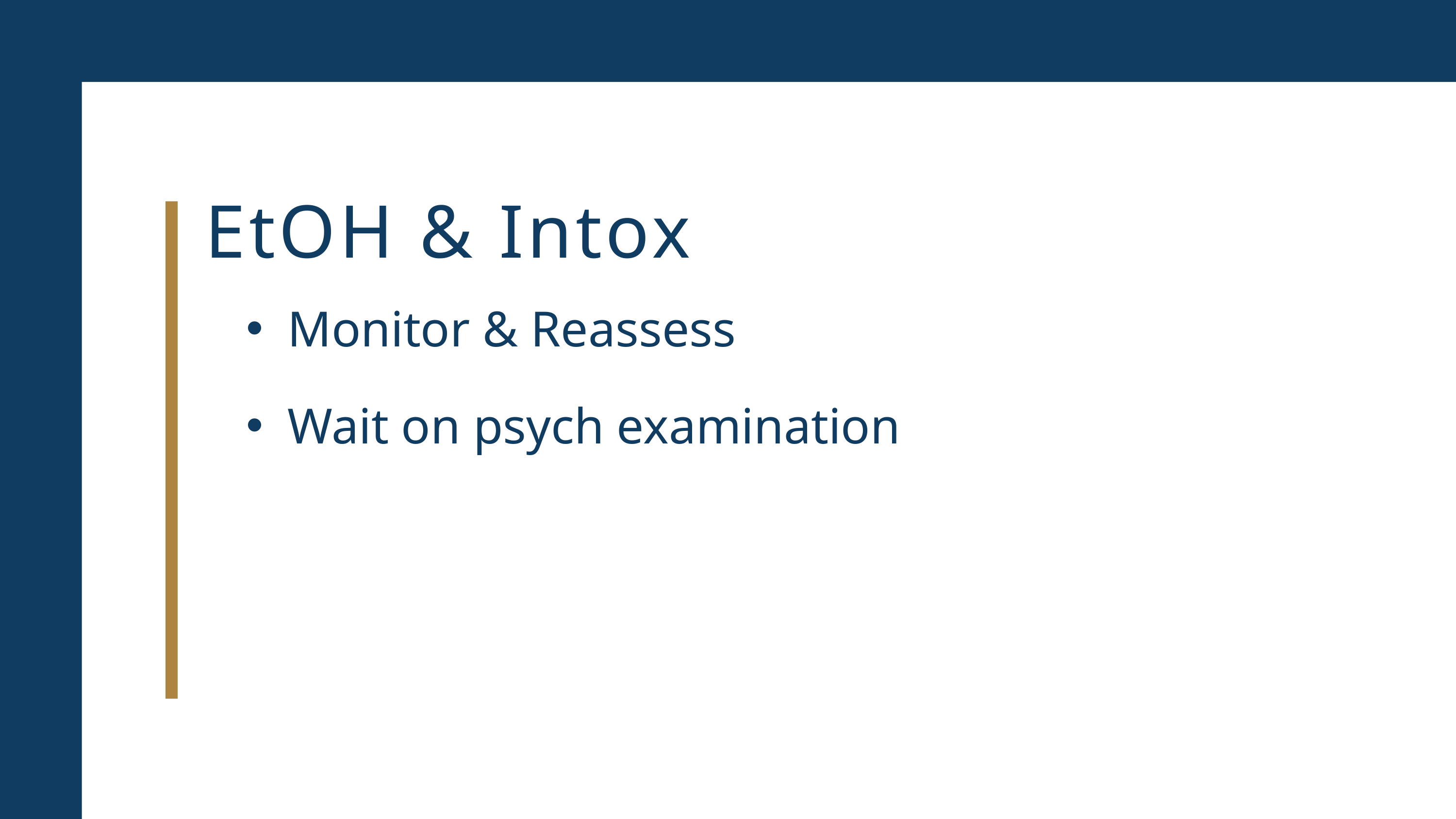

EtOH & Intox
Monitor & Reassess
Wait on psych examination

## Slide 28
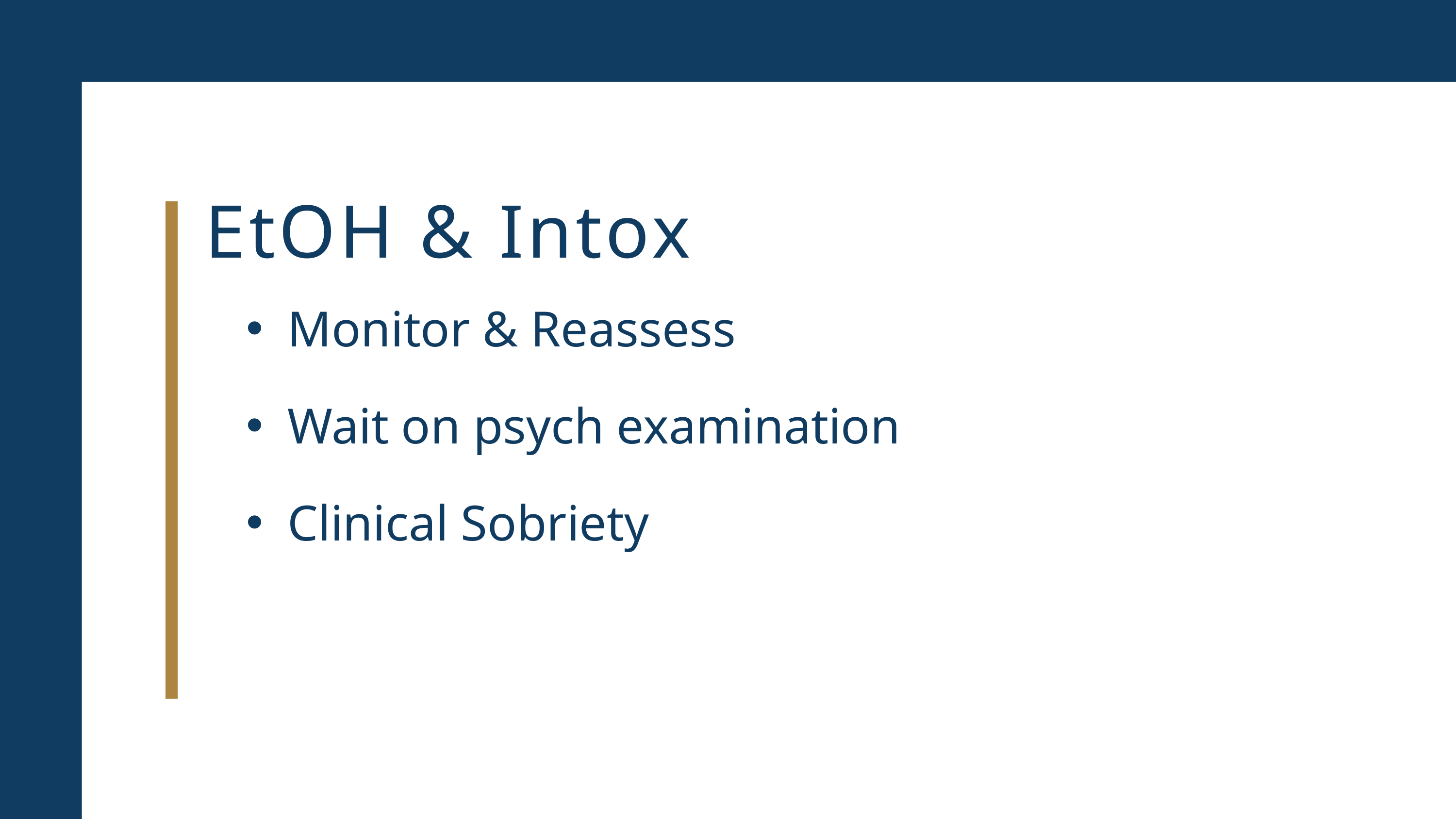

EtOH & Intox
Monitor & Reassess
Wait on psych examination
Clinical Sobriety

## Slide 29
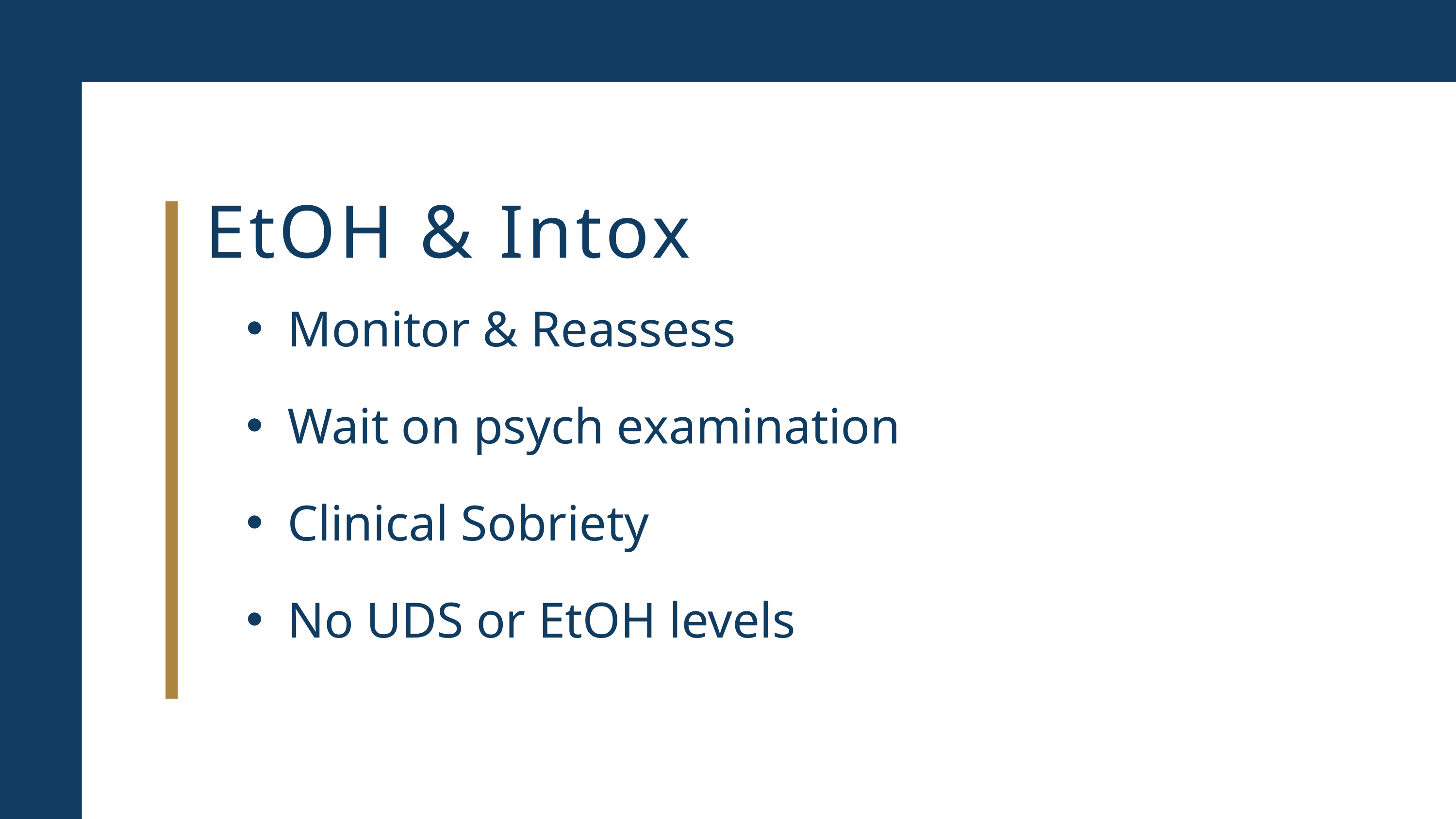

EtOH & Intox
Monitor & Reassess
Wait on psych examination
Clinical Sobriety
No UDS or EtOH levels

## Slide 30
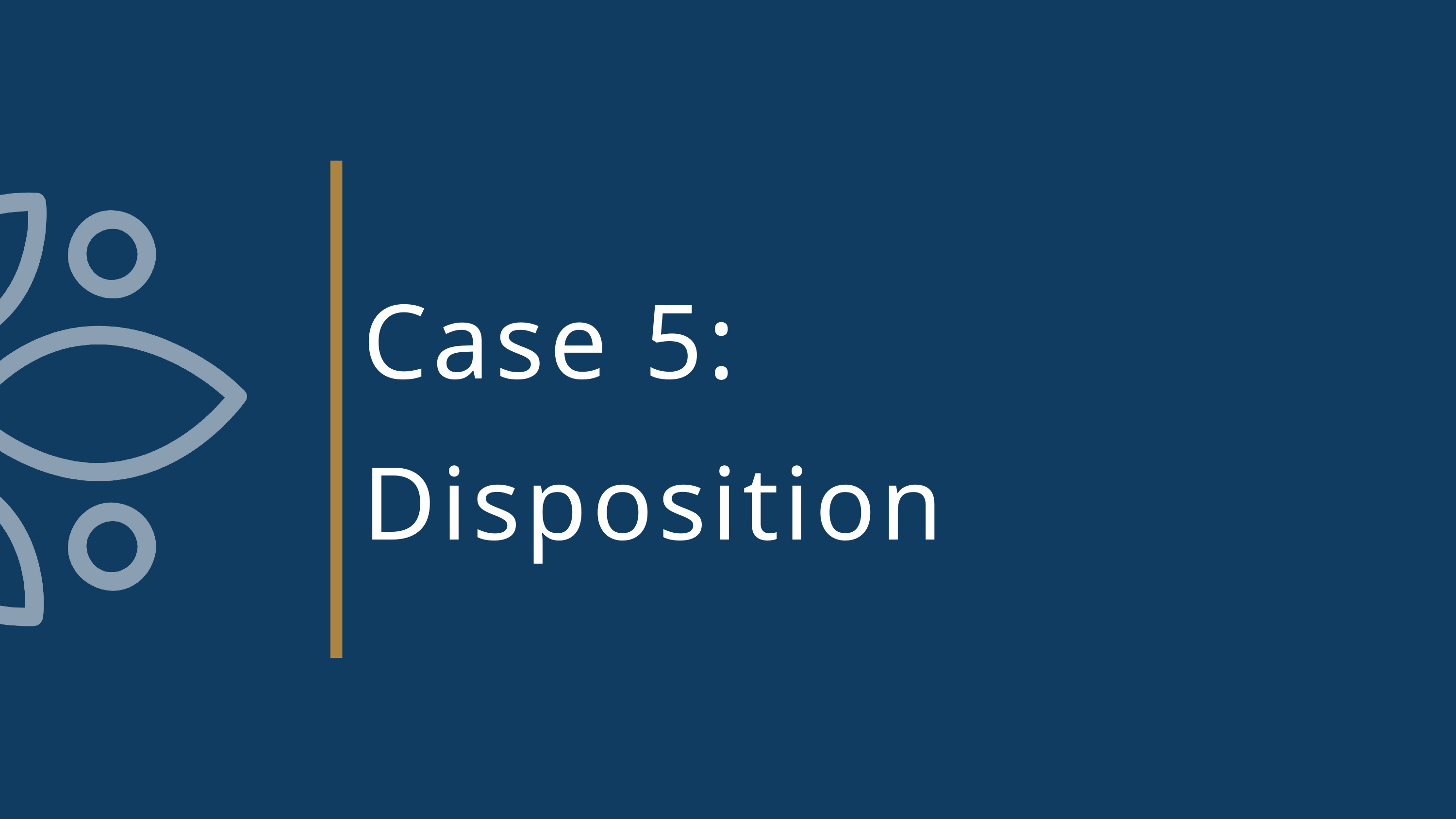

Case 5:
Disposition

## Slide 31
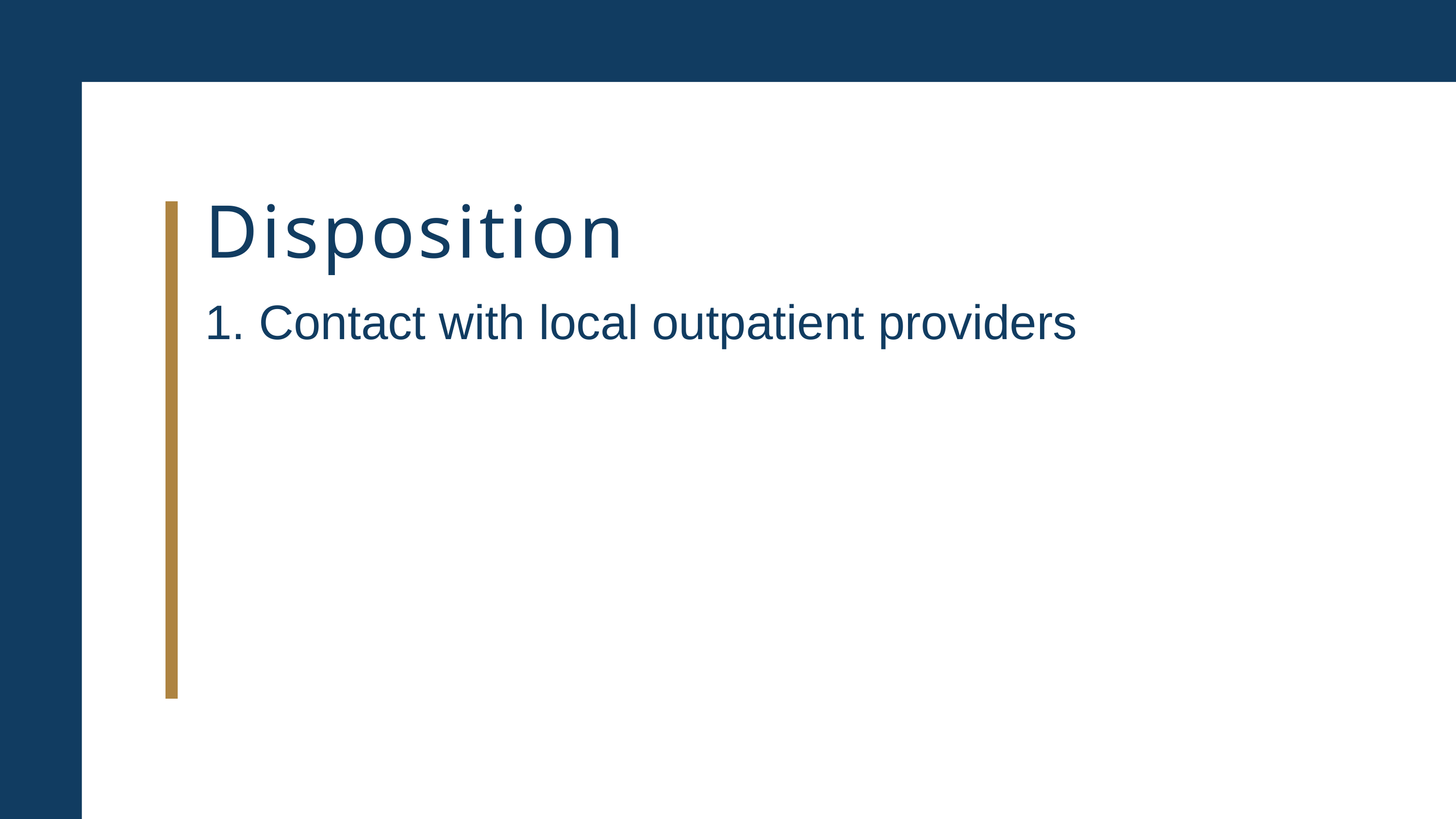

Disposition
1. Contact with local outpatient providers

## Slide 32
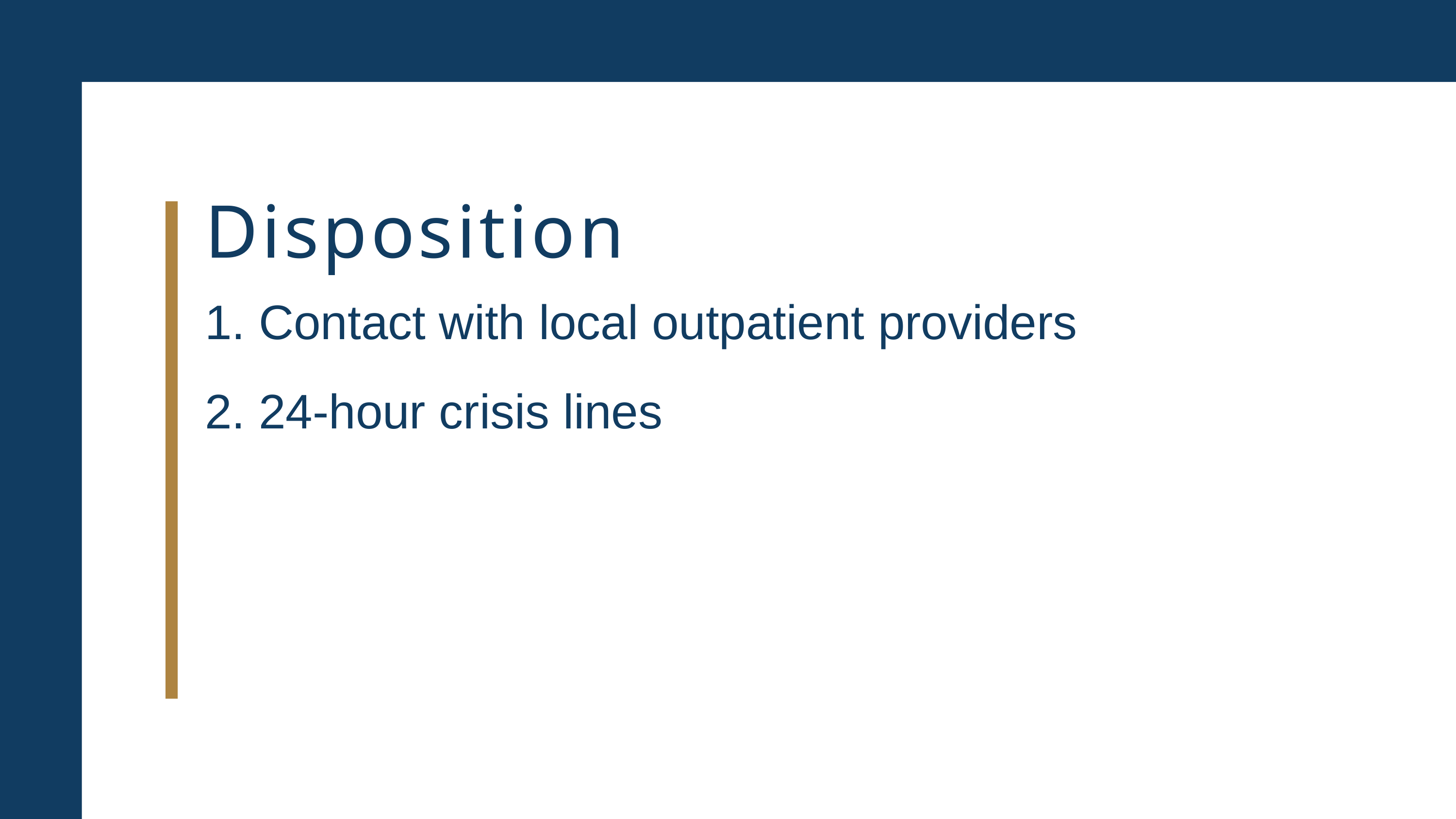

Disposition
1. Contact with local outpatient providers
2. 24-hour crisis lines

## Slide 33
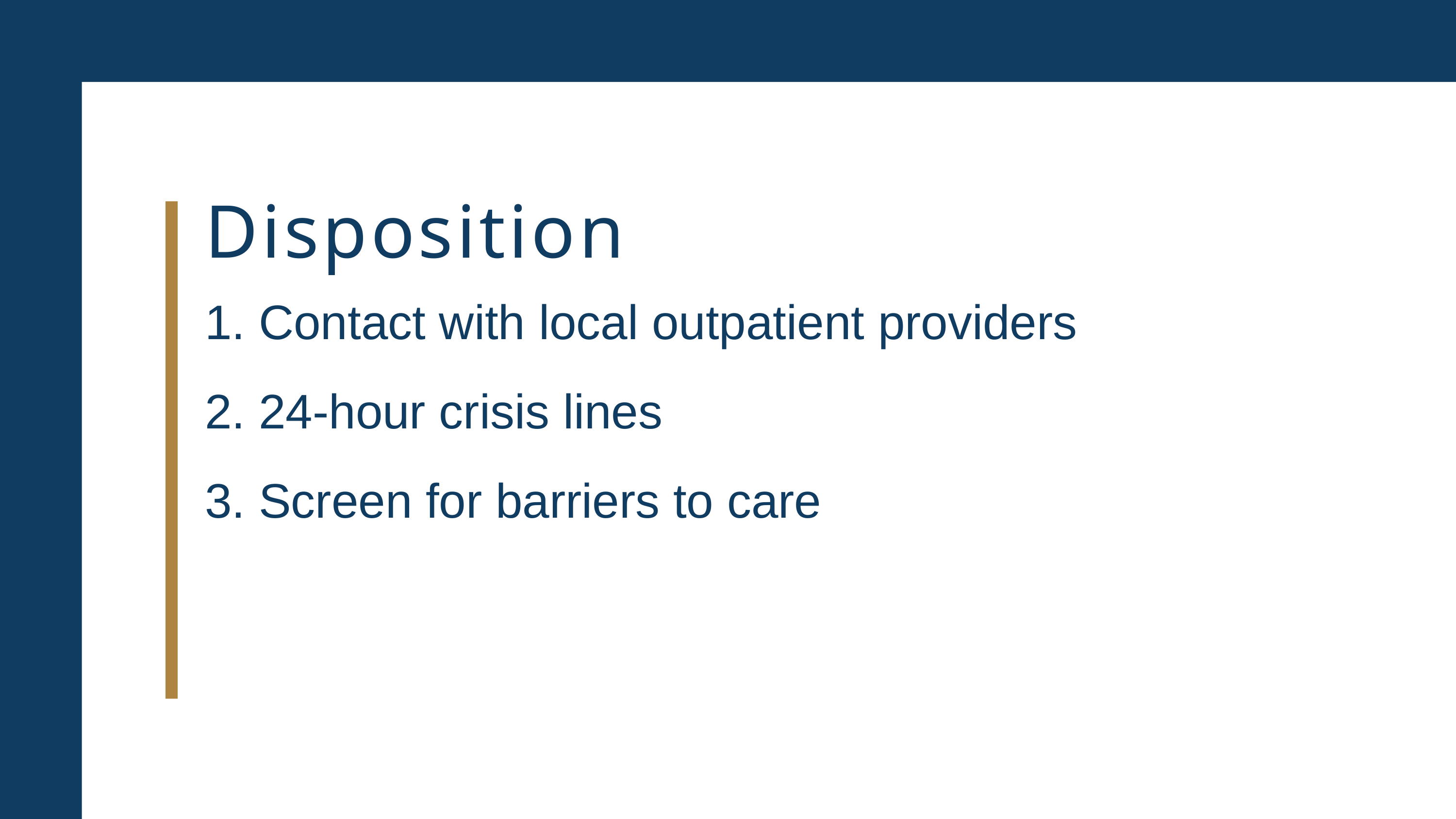

Disposition
1. Contact with local outpatient providers
2. 24-hour crisis lines
3. Screen for barriers to care

## Slide 34
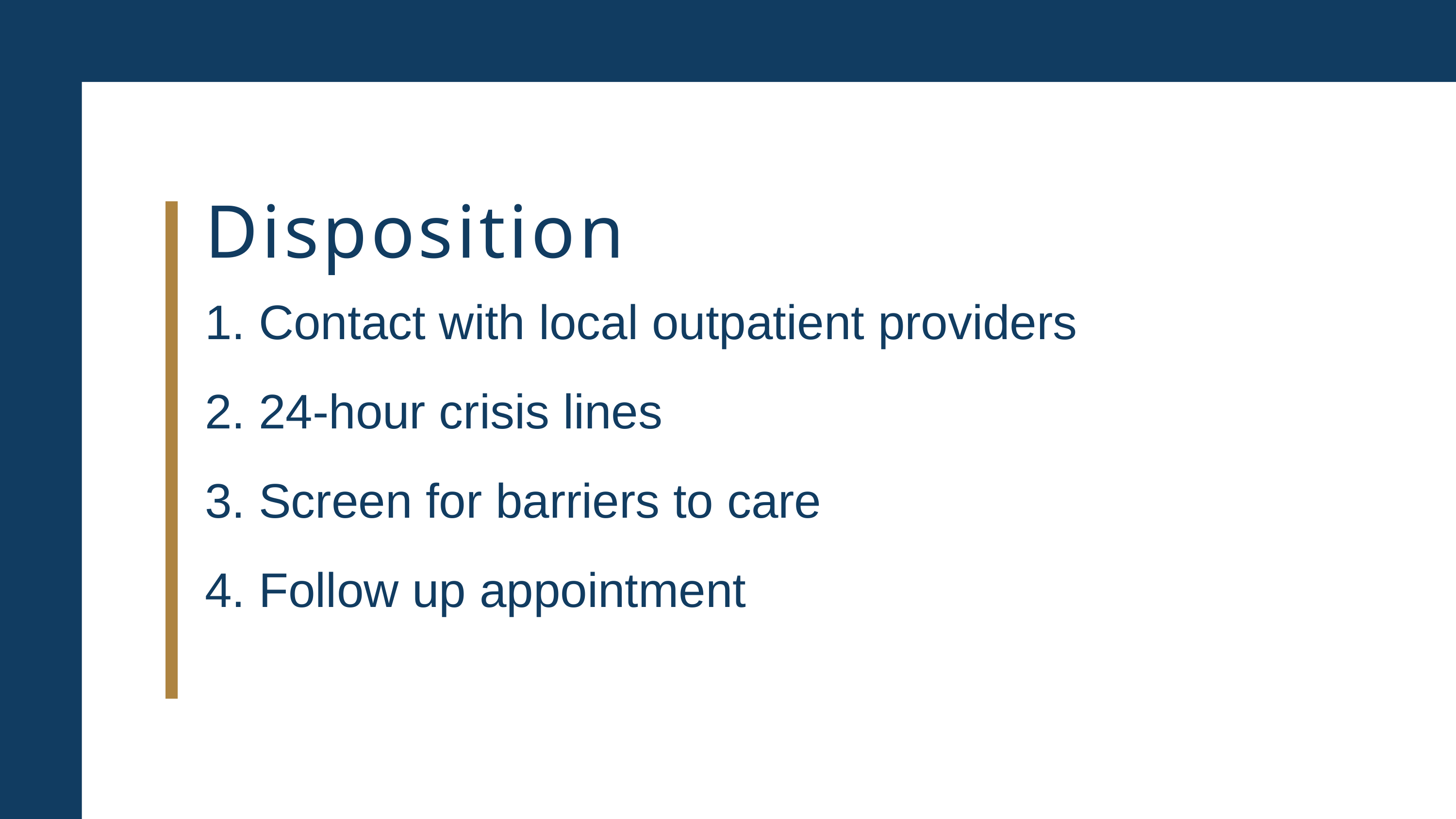

Disposition
1. Contact with local outpatient providers
2. 24-hour crisis lines
3. Screen for barriers to care
4. Follow up appointment

## Slide 35
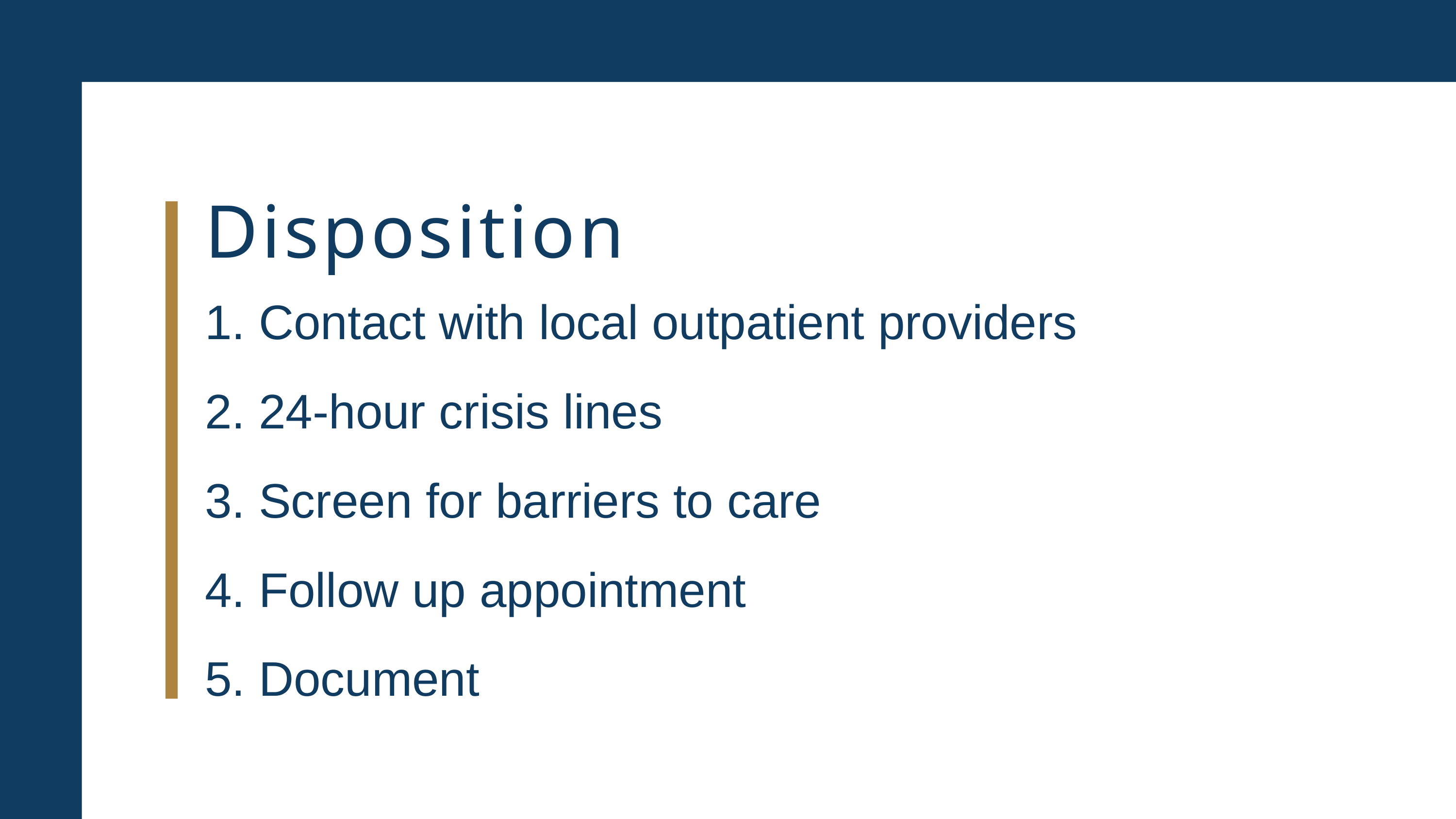

Disposition
1. Contact with local outpatient providers
2. 24-hour crisis lines
3. Screen for barriers to care
4. Follow up appointment
5. Document

## Slide 36
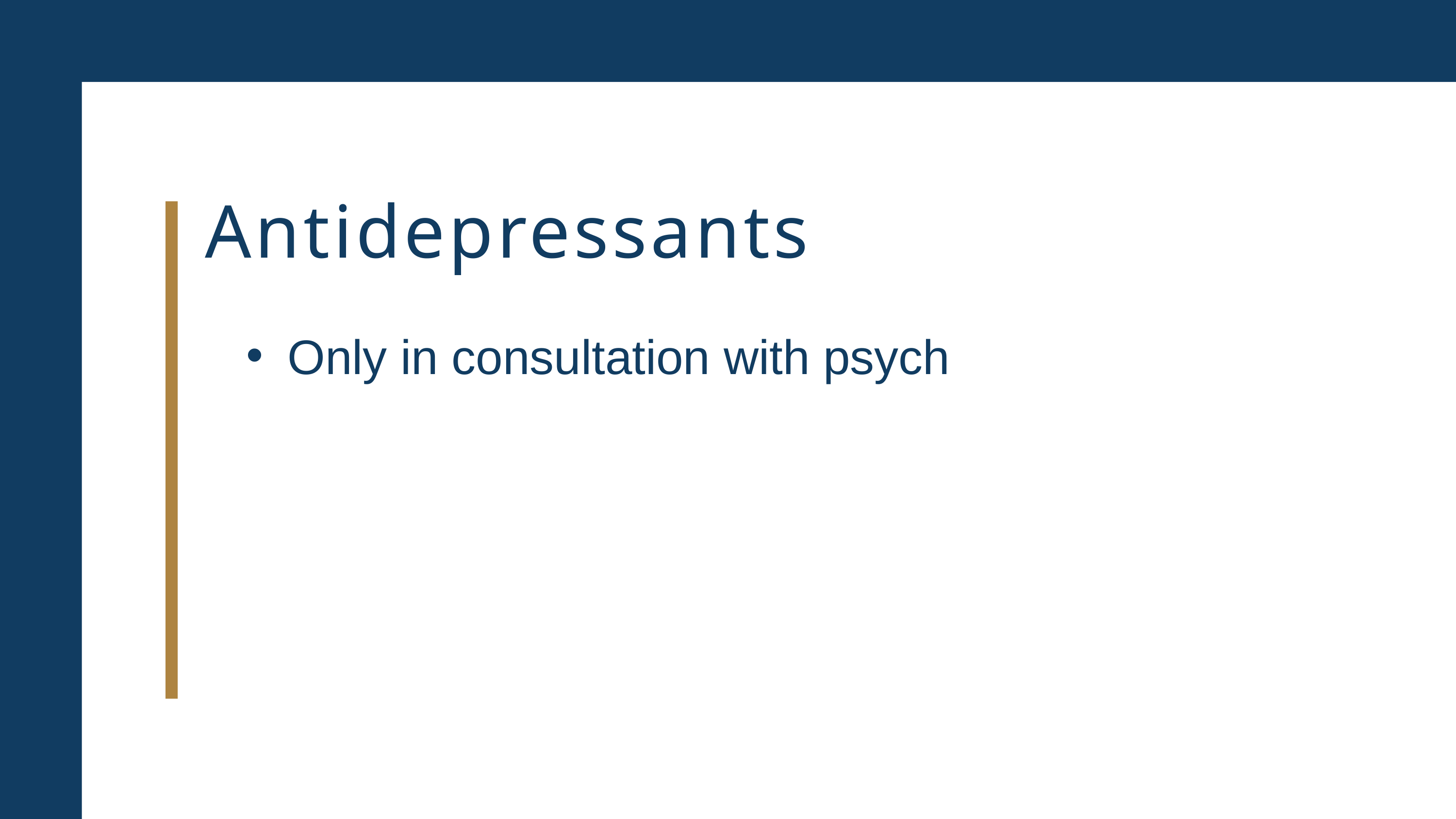

Antidepressants
Only in consultation with psych

## Slide 37
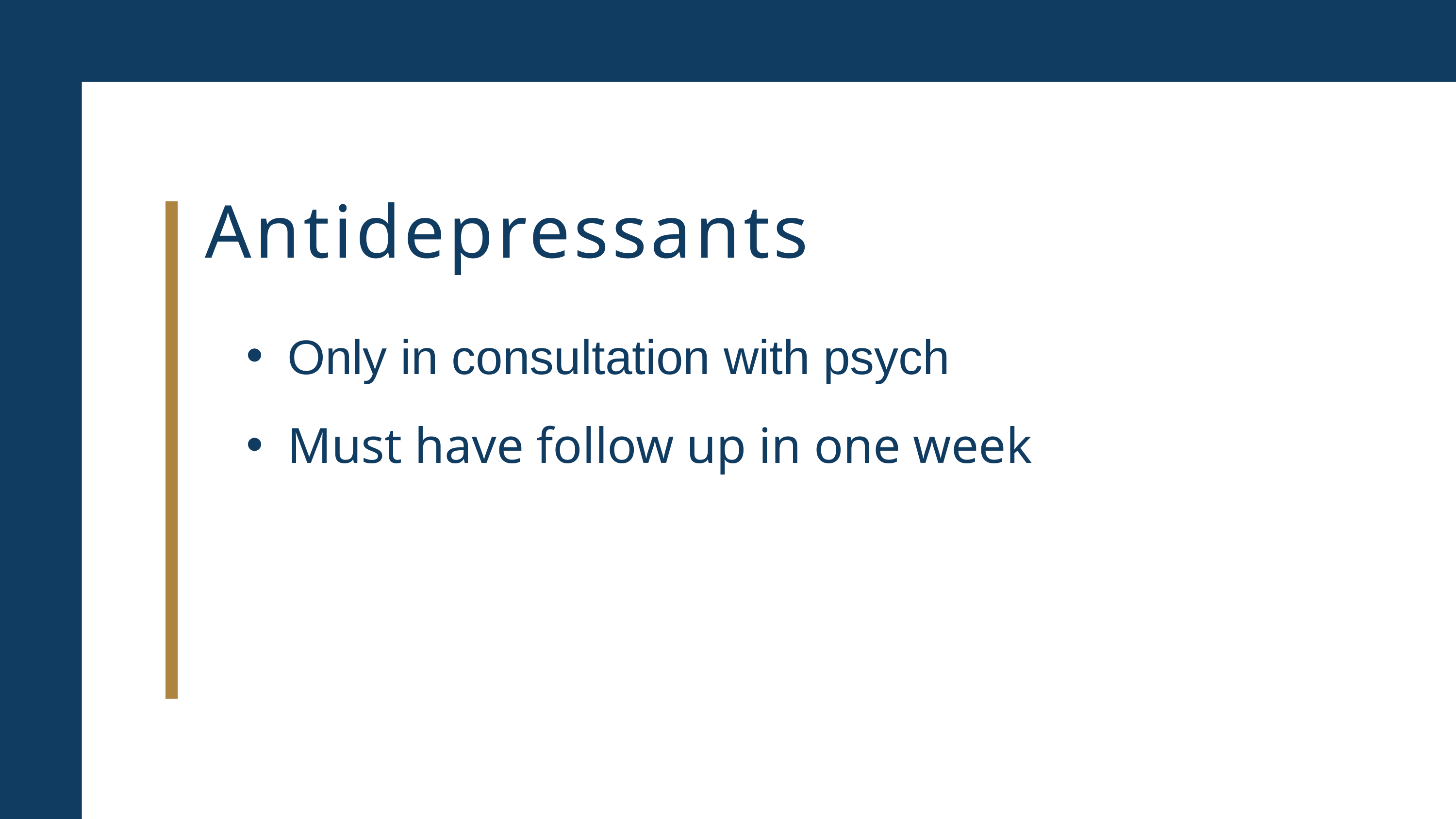

Antidepressants
Only in consultation with psych
Must have follow up in one week

## Slide 38
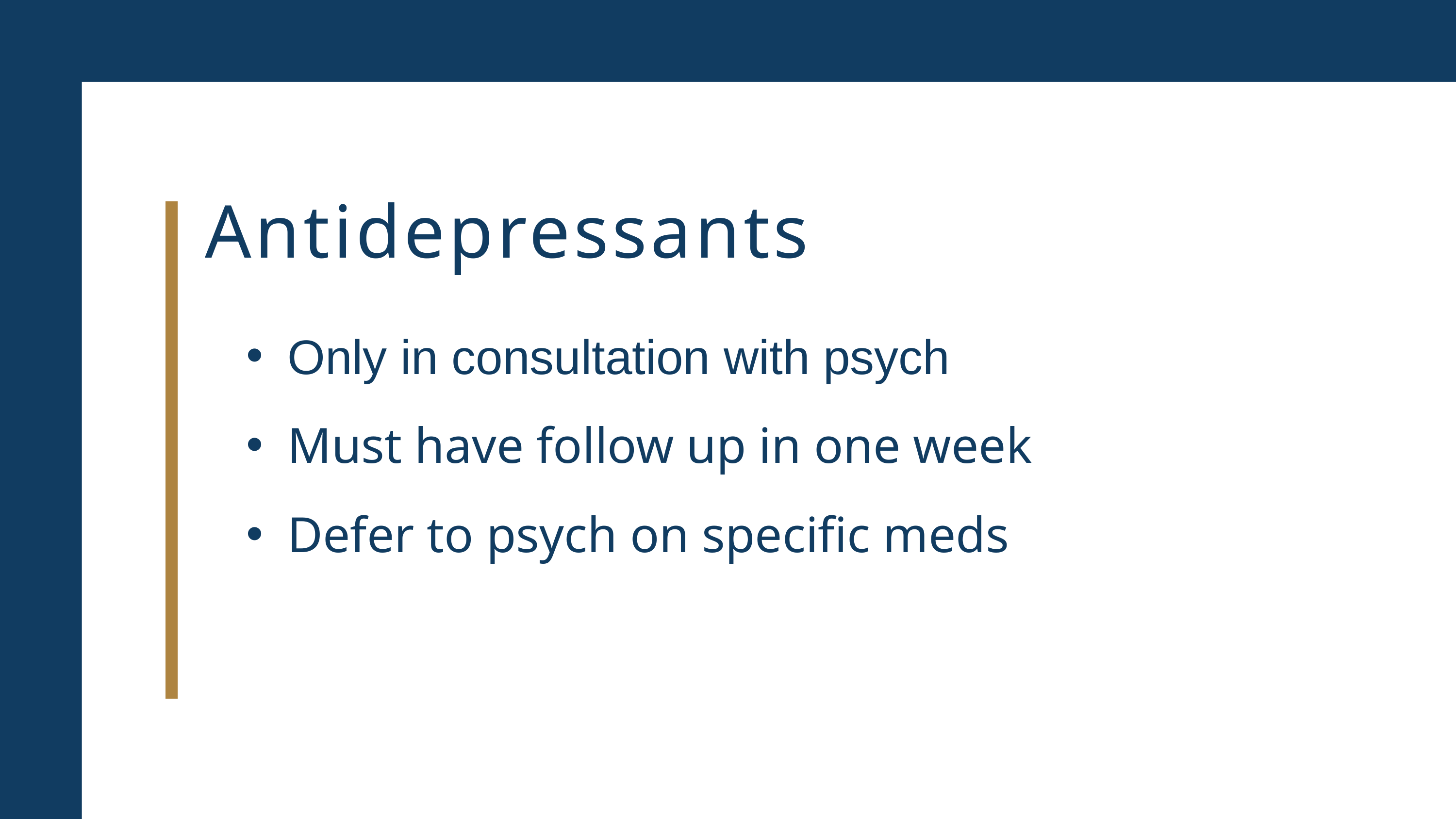

Antidepressants
Only in consultation with psych
Must have follow up in one week
Defer to psych on specific meds

## Slide 39
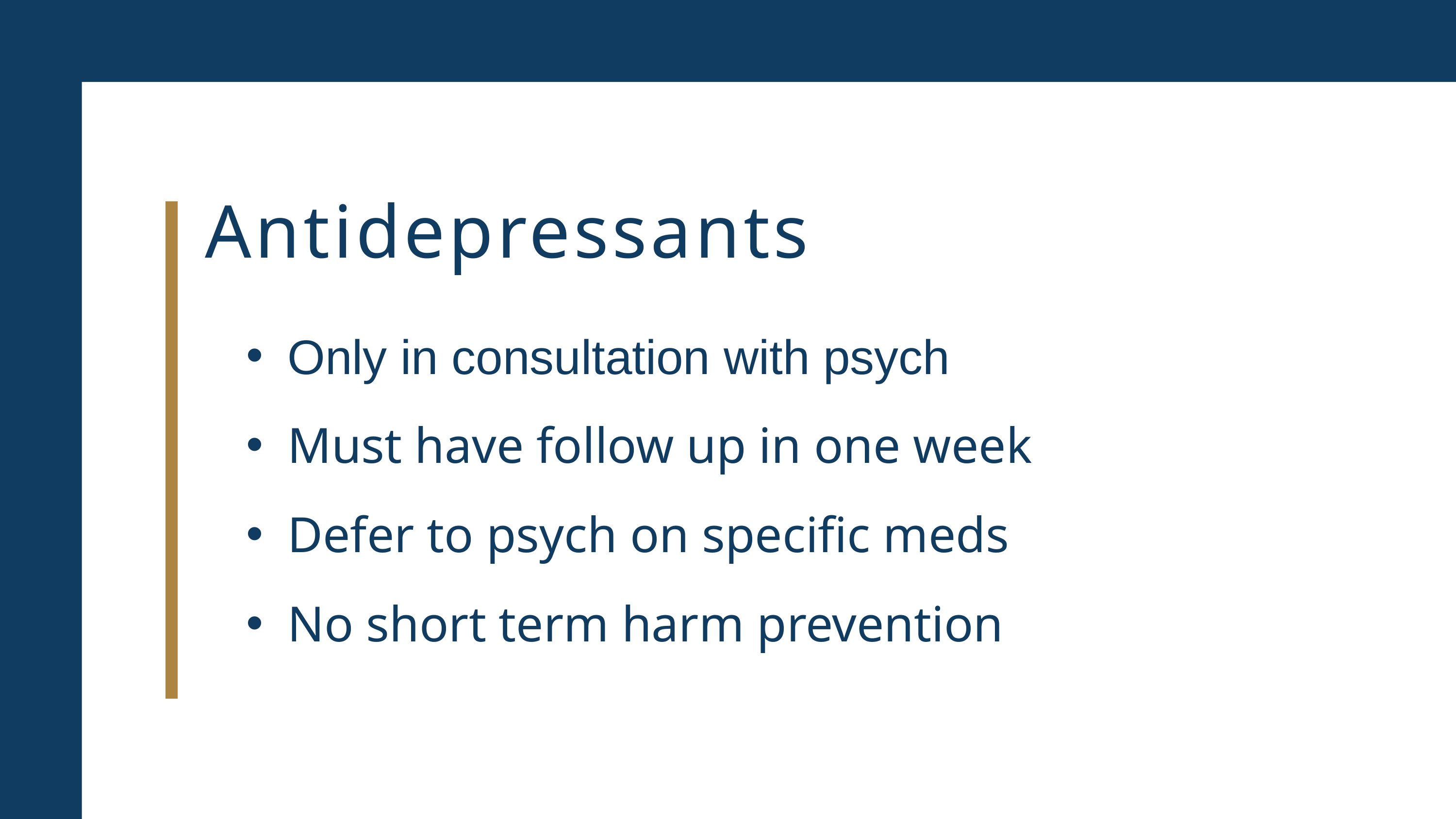

Antidepressants
Only in consultation with psych
Must have follow up in one week
Defer to psych on specific meds
No short term harm prevention
